# Randomized Phase I: Safety, Immunogenicity and Mucosal Antiviral Activity in Young Healthy Women Vaccinated with HIV-1 Gp41 P1 Peptide on Virosomes

**DOI:** 10.1371/journal.pone.0055438

**Published:** 2013-02-20

**Authors:** Geert Leroux-Roels, Cathy Maes, Frédéric Clement, Frank van Engelenburg, Marieke van den Dobbelsteen, Michael Adler, Mario Amacker, Lucia Lopalco, Morgane Bomsel, Anick Chalifour, Sylvain Fleury

**Affiliations:** 1 Center for Vaccinology (CEVAC), Ghent University Hospital, Ghent, Belgium; 2 Kinesis Pharma B.V., Breda, The Netherlands; 3 Chimera Biotec GmbH, Dortmund, Germany; 4 Pevion Biotech AG, Ittigen/Bern, Switzerland; 5 Division of Immunology, Transplantation and Infectious Diseases, San Raffaele Scientific Institute, Milan, Italy; 6 Mucosal Entry of HIV-1 and Mucosal Immunity, Cell Biology and Host Pathogen Interactions Department, Cochin Institute, Université Paris Descartes, Paris, France; 7 CNRS UMR8104, Paris, France; 8 INSERM U1016, Paris, France; 9 Mymetics Corporation, Epalinges, Switzerland; Istituto Superiore di Sanità, Italy

## Abstract

Mucosal antibodies harboring various antiviral activities may best protect mucosal surfaces against early HIV-1 entry at mucosal sites and they should be ideally induced by prophylactic HIV-1 vaccines for optimal prevention of sexually transmitted HIV-1. A phase I, double-blind, randomized, placebo-controlled trial was conducted in twenty-four healthy HIV-uninfected young women. The study objectives were to assess the safety, tolerability and immunogenicity of virosomes harboring surface HIV-1 gp41-derived P1 lipidated peptides (MYM-V101). Participants received placebo or MYM-V101 vaccine at 10 μg/dose or 50 μg/dose intramuscularly at week 0 and 8, and intranasally at week 16 and 24. MYM-V101 was safe and well-tolerated at both doses administered by the intramuscular and intranasal routes, with the majority of subjects remaining free of local and general symptoms. P1-specific serum IgGs and IgAs were induced in all high dose recipients after the first injection. After the last vaccination, vaginal and rectal P1-specific IgGs could be detected in all high dose recipients. Approximately 63% and 43% of the low and high dose recipients were respectively tested positive for vaginal P1-IgAs, while 29% of the subjects from the high dose group tested positive for rectal IgAs. Serum samples had total specific IgG and IgA antibody concentrations ≥0.4 μg/mL, while mucosal samples were usually below 0.01 μg/mL. Vaginal secretions from MYM-V101 vaccinated subjects were inhibiting HIV-1 transcytosis but had no detectable neutralizing activity. P1-specific Th1 responses could not be detected on PBMC. This study demonstrates the excellent safety and tolerability of MYM-V101, eliciting systemic and mucosal antibodies in the majority of subjects. Vaccine-induced mucosal anti-gp41 antibodies toward conserved gp41 motifs were harboring HIV-1 transcytosis inhibition activity and may contribute to reduce sexually-transmitted HIV-1.

**Trial Registration:**

ClinicalTrials.gov NCT01084343

## Introduction

The human immunodeficiency virus type 1 (HIV-1) is mainly transmitted through sexual contact [1]. To infect its host, HIV-1 employs its viral membrane surface trimeric envelope glycoprotein, composed of the receptor binding domain gp120 and the membrane anchored fusion protein subunit gp41 [2], [3]. Pathogen surface proteins are initially detected by the immune system, as they are easily accessible to the antibodies [4]. This feature explains why HIV-1 vaccine developers have traditionally considered the HIV-1 surface gp120/gp41 (gp160) proteins as good vaccine targets [5].

Since HIV-1 discovery in 1983 [6], more than 150 trials have tested different HIV-1 vaccine candidates [7], [8]. These trials have almost exclusively focused on systemic responses and were conducted over 3 chronologically distinct waves of vaccine research to elicit: 1) Neutralizing antibodies [5], [9]–[13]; 2) T cell-mediated immune responses [14]–[20]; 3) Combined neutralizing antibodies and T cell-mediated immunity [21]. Only the RV144 phase III Thailand trial has provided new hope, providing 31% efficacy through the induction of non-neutralizing antibodies and a moderate T cell response [22], [23] measured from blood, while the mucosal immune responses were not investigated during vaccination. In fact, very few human trials have looked at the mucosal immune responses following prophylactic HIV-1 vaccination [24], [25] and vaccine-induced mucosal antibodies were generally not detected. Despite the growing interest for better comprehension of mucosal immunity in the HIV vaccine field, it remains challenging and in its infancy.

The focus on the blood immune responses in the past was likely driven by the following main thoughts: i) The complexity of studying mucosal immunity due to the difficulty of collecting mucosal samples that are generally very limited; ii) Mucosal immunity is too short lived to be monitored; iii) The observed blood immune responses (humoral and cellular immunity) reflect what is happening at the mucosal levels. However, for the latter it was already reported that patterns from paired samples (serum versus vaginal secretion toward the same antigen) were found to be different for antibody specificity [26], and antibody function differences may also exist between blood and mucosa [27], [28]. All these observations are pointing out that both blood and mucosal compartments should ideally be investigated and compared for more accuracy.

HIV-1 rapidly crosses the vaginal or anal mucosa within hours to establish infection. During that period, HIV-1 appears to be susceptible to immune interference [29] and mucosal immunoglobulins may represent an efficient front line defense against sexually transmitted HIV-1 [30]-[32]. An alternative could be the development of prophylactic HIV-1 vaccines capable of eliciting not only circulatory antibodies but also mucosal immune responses for blocking HIV-1 entry at mucosal sites, before primary infection takes place locally in the *lamina propria*. While IgGs may operate in tissues underlying mucosal epithelium and numerous organs throughout the body, mucosal IgAs with compartmentalized distribution and repertoire, combined with their efficient translocation in various mucosal tissues and secretions may best protect mucosal surfaces [30].

The first protective role of antibodies was demonstrated through passive transfer in non-human primates (NHP), using neutralizing IgG antibodies against gp41 and gp120. Early studies have suggested that high serum neutralizing titers were typically required for protection against high-dose SHIV mucosal challenge [5], [12], [33], [34]. More recently, it has been reported that lower serum neutralizing antibody titers were also protecting against repeated low-dose mucosal SHIV challenge [11], [35].

Human evidence supporting mucosal antibodies as protective mechanism came from HIV-1 highly exposed persistently seronegative (HEPS) subjects [36]–[41]. IgAs purified from HEPS were able to neutralize infection of peripheral blood mononuclear cells (PBMC) by HIV-1 isolates [36], [38], [42], [43] and to inhibit HIV-1 transcytosis across mucosal epithelium *in vitro* [37], [38]. These inhibitory mucosal antibodies were shown to be specific to gp41 and the QARILAV motif present on the N-helix was one of the targeted epitopes [44]. A recent study on HEPS women in an HIV-1 serodiscordant relationship has suggested that exposure to an HIV-infected partner with low plasma viral load favors the induction of cervicovaginal IgAs with antiviral activity, which may contribute to reduce HIV-1 acquisition [45]. The membrane proximal external region (MPER) of gp41 is also targeted by the broadly neutralizing IgG monoclonal antibodies 2F5 and 4E10 and more recently by the 10E8 that binds the conserved residues Trp680 and Lys/Arg683 [46]. Although complete *in vivo* protection and sterilizing immunity in NHP were only recently reported for 2F5 and 4E10 [47], these MPER specific antibodies were already known for their ability to block HIV transcytosis and cell infection *in vitro* [3], [48]–[52], as also observed with mucosal IgAs from HEPS individuals [53]. All these observations suggest that gp41 might be an attractive antigen to be included in prophylactic HIV-1 vaccines.

Gp41 is more conserved than gp120 and mediates the fusion process with the target cell membrane [54]. It also contains the conserved mucosal receptor binding motif used by HIV-1 for binding to the galactosyl-ceramide present on epithelial and dendritic cells, which corresponds to the P1 peptide originally defined by the gp41 sequence 650–685 [55]. In a previous study [27], the P1 and a truncated trimeric recombinant gp41 protein (rgp41) were modified for allowing lipidation and surface anchorage on virosome, also called immunopotentiating reconstituted influenza virosome (IRIV). Virosome has a dual function [56], [57]: i) Lipid carrier for antigen delivery, mimicking the native viral membrane environment [58], which may be important for gp41 antigens [59]; and ii) A safe human adjuvant. In contrast to what was observed with many viral vectors, pre-existing antibodies against the influenza hemagglutinin (HA) on virosomes may help to deliver the antigens/virosomes to antigen presenting cells [60], [61] and they are not preventing vaccination with virosomes [57], [62], [63].

The HIV-1 candidate vaccine constituted of virosome-P1 and virosome-rgp41 led to the “Proof of Concept” that vaccine-induced mucosal antibodies protect NHP against vaginal heterologous virus challenges [27]. All animals immunized by the combined intramuscular (priming) and intranasal routes (boost) were fully protected, as compared to 50% protection for the animal group that received only intramuscular vaccinations. Protection was correlated with the presence of gp41-specific vaginal secretions exhibiting transcytosis inhibition and antibody-dependent cell-mediated cytotoxicity (ADCC), while serums had no detectable antiviral activities *in vitro.* These results have clearly challenged the paradigm that mucosal protection against sexually transmitted HIV-1 requires the presence of serum IgGs with virus neutralizing capacity.

Today, it is broadly accepted that antibodies with various antiviral functions and from different immune compartments could play complementary roles for optimal protection [28]. The next step following encouraging NHP studies was to demonstrate that similar blood and mucosal antibodies in women could be induced with the virosome-gp41 approach. It was strategically decided to focus on the P1 antigen before evaluating in clinic the combined P1 and rgp41 formulation.

Heterosexual contact is the primary mode of HIV-1 infection worldwide and it is a rare event [64]. Depending on clinical studies, HIV transmission probability per unprotected coital act may range from 1 in 200–2000 for male-to-female transmission, 1 in 200–10,000 for female-to-male transmission and 1 in 10–1600 for male-to-male transmission. About 60% of newly HIV-infected persons are women and young girls [65], which may also lead to mother-to-child HIV-1 transmission if not treated. It was decided to explore first the safety and immunogenicity of the vaccine in the dominant target population, while testing in men is planned for subsequent trials.

Here we present an exploratory Phase I “Proof of Principle” conducted in healthy young women with the primary objective to determine the safety and tolerability of virosome-P1 (MYM-V101). The second objective was to evaluate its immunogenicity in the serum, while monitoring specific antibodies at the vaginal and rectal levels and conducting limited functional assays were part of the ancillary objectives.

## Methods

### Study design and ethics

This monocenter double-blind, randomized, placebo-controlled Phase I study was registered at www.clinicaltrials.gov (registration NCT01084343) and conducted at the Center for Vaccinology (CEVAC), Ghent University Hospital (Belgium). The protocol for this trial and supporting CONSORT checklist are available as supporting information; see Protocol S1 and Checklist S1 and. Trial protocol, substantial amendments, signed written subject information/Informed Consent Forms (ICFs) and subject diaries were reviewed by the Independent Ethics Committee (IEC) that gave on October 8^th^ 2009 the written approval for this study conducted in accordance with the Declaration of Helsinki and Good Clinical Practice. The approbation and authorization to conduct the clinical trial was also received from the Federal Agency for Medicines and Health Products (FAGG) in Belgium. Due to the exploratory character of the study, no formal power calculation was performed.

The primary objective of the trial was to evaluate the safety and tolerability of two doses of a candidate prophylactic HIV-1 vaccine administered by the intramuscular and intranasal routes. The secondary objective of this Phase I was to verify the immunogenicity of the vaccine candidate by the quantification of P1-specific IgGs and IgAs in the serum. Ancillary studies were also conducted with vaginal and rectal secretions to assess the presence of mucosal P1-specific IgGs and IgAs and their capacity to block *in vitro* HIV-1 infection and transcytosis. PBMC were also isolated to measure the cellular immune responses.

Double-blinding of volunteers' randomization, data management, and descriptive statistics for the analysis of safety, tolerability and immunogenicity data were conducted by Kinesis Pharma B.V. (Netherlands) and M.A.R.C.O. GmbH & Co (Germany). Type of randomisation was 2 strata of 12 subjects each. In each stratum a weight of 2:1 (active treatment:placebo) was used, no blocks were used within a stratum.

**Figure 1:**
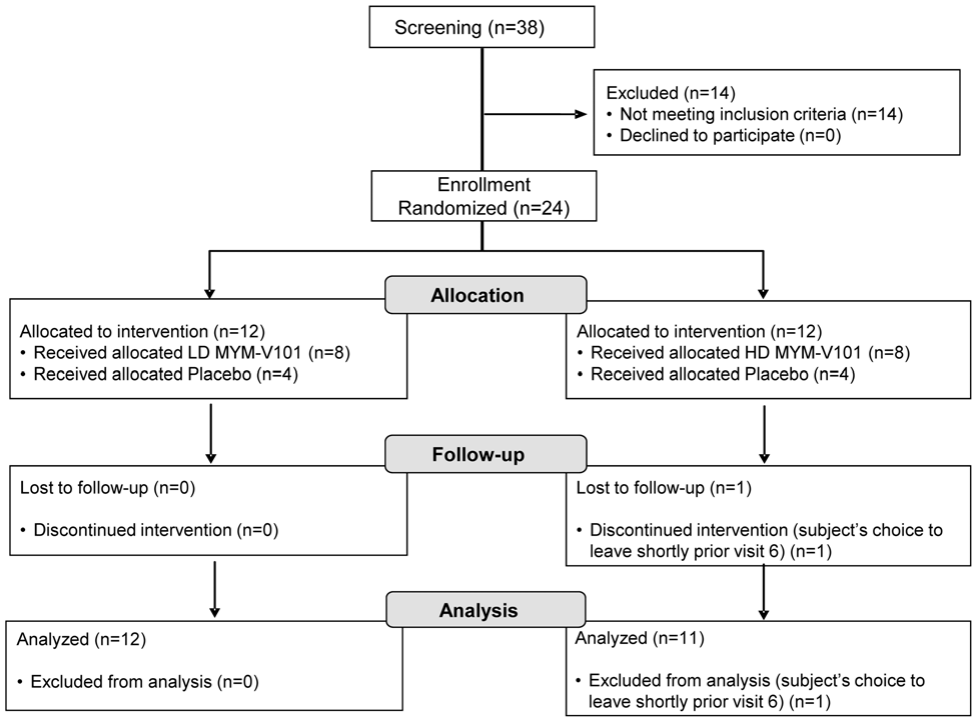
Study flow chart. All subjects screened, enrolled and randomized are indicated in the chart. A total of 24 subjects divided over 2 panels of 12 subjects, which was deemed sufficient to meet the objectives of this trial. The study encompassed 12 medical visits, supplemented by physical examination, recording of vital signs and body temperature, as well as collection of blood samples for safety evaluation. Interim safety reviews have been done prior to the second vaccine administration for each route and prior to start of the HD regimen. Serum (week −3, **0**, 1, 4, **8**, 9, **16**, 17, **24**, 25 and 29), vaginal (week −3, 17, 25 and 29) and rectal samples (week −3 and 25) were taken for secondary endpoint and ancillary studies. To evaluate the cellular immune response, PBMC were isolated on weeks −3, 1, 9, 17 and 25. One subject from HD withdrew consent after Visit 5/week 9, which includes the first two intramuscular injections and performed the early withdrawal visit at the time point of week 16. Consequently, safety and immunogenicity analyses were done only from visit 1 to 5. Rectal samples of 2 LD subjects at Baseline were discarded by mistake during sample work-up. Without baseline data, induction of rectal specific antibodies above baseline could not be estimated. LD: Low dose; HD: High dose. Allocation to a panel was done in consecutive order; Panel 1 (LD: number 101 to 112) was filled first followed by Panel 2 (HD: number 201 to 212). In each panel, 8 subjects received active treatment with MYM-V101 and 4 subjects received placebo MYM-IRIVm or MYM-IRIVn in a double-blind way.

Twenty four (24) Caucasian women were included and randomized in 2 groups (Figure 1 showing the Flow Chart) and enrolled by CEVAC, who has been responsible for the sample work up of the collected materials: A low dose (LD) group (10 µg/dose) and a high dose (HD) group (50 µg/dose). In each group, 8 subjects received the active treatment with MYM-V101 and 4 subjects received the placebo (MYM-IRIV). Four vaccine doses were administered; two intramuscular injections at week 0 and 8, and two intranasal administrations at week 16 and 24. Day of vaccination was to match the woman's cycle (see figure addendum S2 in Protocol S1), with vaccine administration between Days 1 to 5 after ovulation, allowing medical visits to take place 6 to 8 days later to monitor antibody levels. This time frame corresponds to a period of the menstrual cycle with lower risk of antibody contamination originating from menstrual bleeding and lower mucus antibody trapping that could reduce the recovery yield of antibodies. There were no deviations for the intramuscular injections, all were administered according to the protocol, while a single minor deviation for one subject during the 4^th^ administration (second intranasal administration) was reported and it had no impact on the data. The study encompassed 12 medical visits: Screening/week −5, Baseline/week −3, Visit 1/week 0, Visit 2/week 1, Visit 3/week 4, Visit 4/week 8, Visit 5/week 9, Visit 6/week 16, Visit 7/week 17, Visit 8/week 24, Visit 9/week 25 and Visit 10/week 29. Data of subjects receiving placebo were pooled for comparison with the low and high dose groups.

### Study population

Participants were negative for serological markers of HIV-1, HIV-2, HAV, HBV and HCV infections, aged between 18 and 45 years with a body mass index (BMI) between 18.0–30.0 kg/m^2^ (Table 1) and were not suffering or had not suffered from recurrent vaginal infections or sexually transmitted diseases within one year prior to vaccination. Women had regular menstrual cycles (24 to 30 days) and were healthy, based on a medical evaluation revealing no clinically relevant abnormality after physical and gynecological examinations, medical history, electrocardiogram, vital signs, blood biochemistry and hematology tests, urinalysis. Subjects with childbearing potential agreed to use adequate oral contraception or physical barrier contraceptives. Descriptive statistics (n, arithmetic mean, standard deviation, median, minimum, and maximum) were generated at all visits by treatment group.

**Table 1:** Characteristics of the study population.

	Placebo n = 8	MYM-V101 (Low Dose) n = 8	MYM-V101 (High Dose) n = 8
**Age (year)**			
Mean (SD)	23.3 (3.0)	24.1 (3.5)	23.6 (3.2)
Median	22.0	23.0	23.0
Range	20–28	19-29	19–29
**Weight (kg)**			
Mean (SD)	66.5 (7.4)	66.4 (12.7)	58.7 (7.6)
Median	66.2	66.6	61.0
Range	57–79	51–82	47–69
**Height (cm)**			
Mean (SD)	169.9 (4.6)	169.0 (7.9)	166.8 (6.3)
Median	171.0	166.0	167.0
Range	162–176	159-182	158–176
**BMI (kg/m2)**			
Mean (SD)	23.1 (3.3)	23.1 (3.6)	21.1 (2.1)
Median	22.4	22.3	20.8
Range	19–30	19–30	18–24
**Ethnicity (n)**			
Caucasian	8	8	8

### Study vaccines

The original published P1 peptide sequence [55] was modified (SQTQQEKNEQELLELDKWASLWNWFDITNWLWYIKLSC) for allowing lipidation of the peptide for anchorage into the virosome membrane and to accommodate industrial up-scaling. GMP manufacturing was according to ICH Q7 (Bachem AG, Switzerland). Vaccine formulations were manufactured by Pevion Biotech AG, as previously described [27], [66]. Vaccine MYM-V101 **m** for intramuscular injections (0.5 mL, 23G x 1 inch needles) were at 20 µg/mL (LD) or 100 µg/mL (HD) of specific P1 content, while MYM-V101**n** for intranasal administration by BD Accuspray™ (0.1 mL/nostril) were at 50 µg/mL (LD) or 250 µg/mL (HD), all containing about 10 µg of influenza hemagglutinin per dose unit. Placebo consisted of influenza-virosome without P1: MYM-IRIVm or MYM-IRIVn. The vaccines were packaged and labeled in double-blinded manner. A minimal shelf-life of nine months was demonstrated. Modification of P1 by oxidation and de-amidation over time was observed by LC-MS. For safety concerns, dose adjustments consisting in a reduction of 40–50% of lipidated P1 for the last 3 injections were made in the HD group.

### Safety and reactogenicity evaluation

The primary endpoints within one week after each vaccination were: 1) Solicited local symptoms (redness, swelling, pain in case of intramuscular vaccination and nasal congestion, runny nose, impaired smelling and headache in case of intranasal vaccination); 2) Solicited general symptoms (body temperature, tiredness, gastro-intestinal complaints, malaise, muscle pain). Serum C-reactive protein (CRP) values were used as secondary endpoint for the safety analysis. Descriptive statistics (n, arithmetic mean, standard deviation, median, minimum, and maximum) were computed for each symptom and each time point (days 0 to 6 after vaccinations) by treatment group. Spontaneous adverse events (AEs) that might have occurred from Weeks 2–8 after each vaccination were also recorded. The study encompasses a Baseline/week −3 visit and 10 medical visits for monitoring occurrence of hematological and biochemical abnormalities from blood. Safety data of subjects receiving placebo were pooled for comparison with the low and high dose groups.

### Collecting serum and mucosal samples

Blood samples were drawn by venipuncture, using vacuum collection tubes. Vaginal samples at Baseline were obtained by four sequential vaginal harvestings in different segments at 1 minute interval, using pre-wetted Weck-Cel® sponges (Eyetec Ophthalmic product, Altomed Ltd., UK) with 50 μL of sterile PBS that were placed gently in the vagina and allowed to passively adsorb secretions for approximately 30 seconds, as previously described [67], [68]. For post-vaccination visits at week 17, 25 and 29, only two sequential harvestings were performed. Rectal samples were obtained at Baseline and week 25, following two sequential harvestings at 5 minutes interval with Weck-Cel®. Each sponge was macroscopically checked for blood traces and weighed prior being placed in the upper part of a sterile Spin-X centrifuge tube filter. Vaginal extraction from the sponge was performed as follow: Each sponge was equilibrated with 300 μL of extraction buffer for 30 minutes at 4°C, as previously described: PBS, 0,25 M NaCl, supplemented with protease inhibitor cocktail diluted 1/100 from the freshly made master mix, 100 IU/mL penicillin, 100 µg/mL streptomycin. Protease inhibitor cocktail (master mix 100x) was containing 100 µg/mL aprotinine, 500 µg/mL leupeptine, hemisulfate salt, 100 µg/mL bestatine hydrochloride, 50 µg/mL AEBSF (all from Sigma). Following centrifugation at 12,000 g for 20 minutes at 4°C, a second extraction was performed with the same volume and the extracted vaginal secretions of the same subject were pooled, distributed in aliquots and stored at −80°C. All pre-specified laboratory assessments were conducted in a blinded manner.

### Immunogenicity and statistical evaluation

Pre-immune and immune samples were analyzed for total and specific antibody-response on the Immuno-PCR Imperacer® platform [69]-[72], which combines ELISA and PCR technologies, using detecting antibodies conjugated to DNA sequences for improved sensitivity. For analysis of total antibody-response, anti-human IgG or IgA antibodies were used as surface immobilized capture-reagent. For detecting vaccine-induced P1 specific antibodies, surface immobilized P1 peptide was used to which serum or mucosal samples were added, followed by anti-human IgG or IgA antibodies.

The Imperacer®-reader provides Ct value (raw data), the calculated cycle time: Ct  =  the number of PCR cycles needed to reach a uniform threshold of antibody-DNA conjugates (dRn signal), which is the measured fluorescence increase. Ct value is allowing quantitative analysis of analyte against a calibration-curve. As Ct is inversely proportional to the antigen concentration, it was converted to delta Ct (ΔCT) value, which is directly proportional to initial antigen concentration: ΔCt  =  Ct_max_ – Ct value, Ct_max_ corresponding to the maximum number of cycles by PCR.

The precision of the methods was determined in 6 replicates (n = 6) during validation. A cut-off ΔΔCT value was used to define a sample positive for P1-specific antibodies. The ΔΔCT value was calculated as follow: i) The highest standard deviation (SD) value of the assay (0.917) was used as SD cut-off value, which was calculated from the intermediate precision expressed as standard deviation (n = 6) of the assay derived from a negative control sample (mean ΔCT  = 15); ii) This standard deviation was multiplied by the factor 2.177 proposed when n = 6 to calculate the cut-off for 95% confidence level [73] (SD cut-off value  = 0.917 [SD; n = 6; mean ΔCT  = 15] ×2.177 [95% CL; n = 6]  = 1.996); iii) The mean ΔCT value from baseline (pre-immune) was subtracted from the mean ΔCT value from immune sample, leading to ΔΔCT value. Any sample with a ΔΔCT value >1.996 was considered as positive for the presence of P1 specific antibodies. Based on assumptions and internal standard curves, ΔCT values from Imperacer® were converted to approximate antibody concentrations (ng/mL) by analysis against a reference curve. Converting data from arbitrary unit to antibody concentration is an approximate approach and dependent on the technique used. Therefore, the estimated antibody concentrations should be considered only as indicative values.

In this study, immunological parameters were expected to show skewed non-normal distributions. In that case, log transformation of the skewed data was considered. In case of normally distributed log transformed data, these were used in a paired t-test analysis. When the log transformed data were not normally distributed, the Wilcoxon signed rank-sum test was used to compare pre- and post-vaccination results. Because of the explorative nature of this study, there were no adjustment of statistical p values for multiple time point assessments for immunological analysis of both serum and mucosal samples. Significance of statistical results was interpreted only for the time points of primary interest. Statistical testings for other time points were only interpreted as a descriptive tool to help quantify the differences from baseline over time.

### Th1 responses

Cellular Th1 immune responses were measured, as previously described [74]. Briefly, PBMC with viability >95% were cultured *in vitro* in the presence of co-stimulatory anti-CD28 and anti-CD49d monoclonal antibodies (1 µg/mL each; BDIS) with/without P1 peptides (1.25 and 5 µg/mL). Staphylococcal enterotoxin B (SEB; 1 µg/mL; Sigma-Aldrich) was used as a positive control for cell activation. Intracellular cytokine staining was performed to detect IFN*γ*, TNFα and IL-2 in CD3^+^/CD4^+^, CD3^+^/CD8^+^, CD3^+^/CD56^+^ and CD3^−^/CD56^+^ cells.

### Functional *in vitro* assays

Immunoglobulins from some samples were concentrated by ammonium sulfate precipitation [75], [76] for reaching a higher antibody concentration before being tested in neutralization assays. Enriched mucosal antibodies were tested in two neutralization assays (U87.CD4.CCR5 and TZM/bl), using three different HIV-1 strains (JRFL-140WT, QH0692.42 and SF162). 2F5 IgG (5 µg/mL for IC_50_ and 50 µg/mL for IC_90_) was used as positive control. Samples with low antibody concentration were pooled and those with acceptable antibody concentration were tested individually. Neutralization assays were performed as described previously [77], [78]. For inhibition of HIV-1 transcytosis assays [79]–[81], clade B (JR-CSF) HIV-1 infected CCR5-CEM cells were used to inoculate HEC-1 endometrial cell lines cultured in a polarized manner in a two-chamber system. 2F5 IgG was used as positive control (10 µg/mL >90% HIV-1 transcytosis inhibition, data not shown). Percentage of transcytosis inhibition was determined in three independent experiments. Samples were defined as positive when the transcytosis inhibitions were above 50% and reproduced in at least 2 experiments. The 2F5 IgG monoclonal antibody was used as positive control: Transcytosis blockade efficiency  = 100 – Transcytosis efficiency (Transcytosis in presence of vaginal samples from week 25 or week 29/Transcytosis in presence of pre-immune sample) ×100. A qualitative approach (presence of absence of antiviral activity) was used for monitoring the presence of transcytosis inhibition activity. Due to the limited number of samples and assay variability, statistical analyses according to clinical standards were not conducted.

## Results

### Study population

Thirty eight women were screened for eligibility and twenty four were enrolled in the study (Figure 1). The trial was conducted according to the planned protocol: Screening for Low dose and High dose was from November 4^th^ 2009 to January 12^th^ 2010, the treatment/vaccination period was from December 8^th^ 2009 to August 2^nd^ 2010 and the follow up period (post vaccination) was from June 2^nd^ 2010 to September 28^th^ 2010, the latter one corresponding to trial completion after the final medical visit of the last vaccinated subject. Prior to vaccination, medical examinations had shown that all subjects were in good health. The mean age of the Caucasian female subjects of this study was 23.7 years, ranging from 19 to 29 years (Table 1).

### Safety analyses

The majority of subjects remained free of local and general symptoms after vaccination (Table 2 and Table 3). Grade 1 pain at the injection site was reported after the first and second intramuscular dose by two subjects from both LD and placebo groups. In the HD group, Grade 1 pain was reported by four and one persons after the first and second injection, respectively (Table 2). Grade 1 redness was reported only once by a HD recipient after the second injection. No swelling was reported. After the first intranasal administration, one LD and two HD and placebo recipients reported nasal congestion. After the second intranasal dose, two HD and two placebo recipients complained of nasal congestion. Runny nose (Grade 1) or headache (Grade 1 to 3) were reported by only few subjects and smelling impairment by none. CRP values (data not shown) and the incidences of local and general symptoms (including body temperature) appeared to be similar between the different groups, and most of these resolved within 48 h.

**Table 2:** Volunteers experiencing solicited local and general symptoms after intramuscular vaccination.

Group	n	No. of Symptoms	Redness Grade				Swelling Grade				Pain Grade				Body Temp. (^o^C) min-max
			0	1	2	3	0	1	2	3	0	1	2	3	
Placebo															
Injection #1	8	2	8	0	0	0	8	0	0	0	6	2	0	0	35.1–37.5
Injection #2	8	2	8	0	0	0	8	0	0	0	6	2	0	0	35.2–37.3
Low Dose															
Injection #1	8	2	8	0	0	0	8	0	0	0	6	2	0	0	35.6–37.3
Injection #2	8	2	8	0	0	0	8	0	0	0	6	2	0	0	35.3–37.3
High Dose															
Injection #1	8	4	8	0	0	0	8	0	0	0	4	4	0	0	35.3–37.4
Injection #2	8	2	7	1	0	0	8	0	0	0	7	1	0	0	35.9–37.8

**Table 3:** Volunteers experiencing solicited local and general symptoms after intranasal vaccination.

Group	n	No. of Symptoms	Nasal Congestion Grade				Runny Nose Grade				Impaired Smelling Grade				Headache Grade				Body Temp. (^o^C) min-max
			0	1	2	3	0	1	2	3	0	1	2	3	0	1	2	3	
Placebo																			
Injection #3	8	5	6	2	0	0	8	0	0	0	8	0	0	0	5	1/2	0	0	35.3–37.2
Injection #4	8	7	6	1/1	0	0	4	2/2	0	0	8	0	0	0	7	0	1	0	35.4–37.2
Low Dose																			
Injection #3	8	2	7	1	0	0	8	0	0	0	8	0	0	0	7	0	1	0	35.3–37.2
Injection #4	8	2	8	0	0	0	8	0	0	0	8	0	0	0	6	2	0	0	35.9–37.3
High Dose																			
Injection #3	7	7	4	2	1	0	5	2	0	0	7	0	0	0	5	1	1	0	35.4–37.3
Injection #4	7	4	5	2	0	0	6	1	0	0	7	0	0	0	6	0	0	1	35.7–37.2

**Table 2** is showing AEs following intramuscular vaccinations and **Table 3** for intranasal administrations. Local and general symptoms were assessed from Day 0 to Day 6 after each vaccination and spontaneous AEs that might have occurred after each vaccination were also recorded. The study encompasses a Baseline/week −3 visit and 10 medical visits to monitor occurrence of hematological and biochemical abnormalities in blood. Each symptom was graded for severity and assigned causality relative to the study vaccine. Severity was graded as either absent/none (Grade 0), mild (Grade 1, easily tolerated), moderate (Grade 2, interfere with activity of daily living), or severe (Grade 3, prevented activities of daily living). Redness and swelling at the injection site were graded as follow: 0 = <5 mm, 1 = 5–20 mm, 2 = 20–50 mm, 3 = >50 mm. Numbers in bold are vaccine-related symptoms.

Two subjects in LD and one subject in HD reported spontaneous vaccine-related AEs. In LD, one subject had an ecchymosis at the injection site on the day of the first intramuscular vaccination, and she also reported dizziness after the first intramuscular vaccination that resolved on the same day. The other LD subject had an ecchymosis at the injection site following the second intramuscular vaccination. The HD subject reported tickling in the right nostril one minute after the first intranasal vaccination, which resolved on the same day and did not occur after the second intranasal vaccination. All four vaccine-related AEs were of mild intensity and resolved spontaneously. None of the subjects in the placebo group reported a vaccine-related AE. Incidences of non-related AEs did not differ between groups. No AE led to trial discontinuation and no serious AEs were reported. All other safety parameters assessed did not reveal any clinically significant findings.

### Immunogenicity analyses

**Figure 2:**
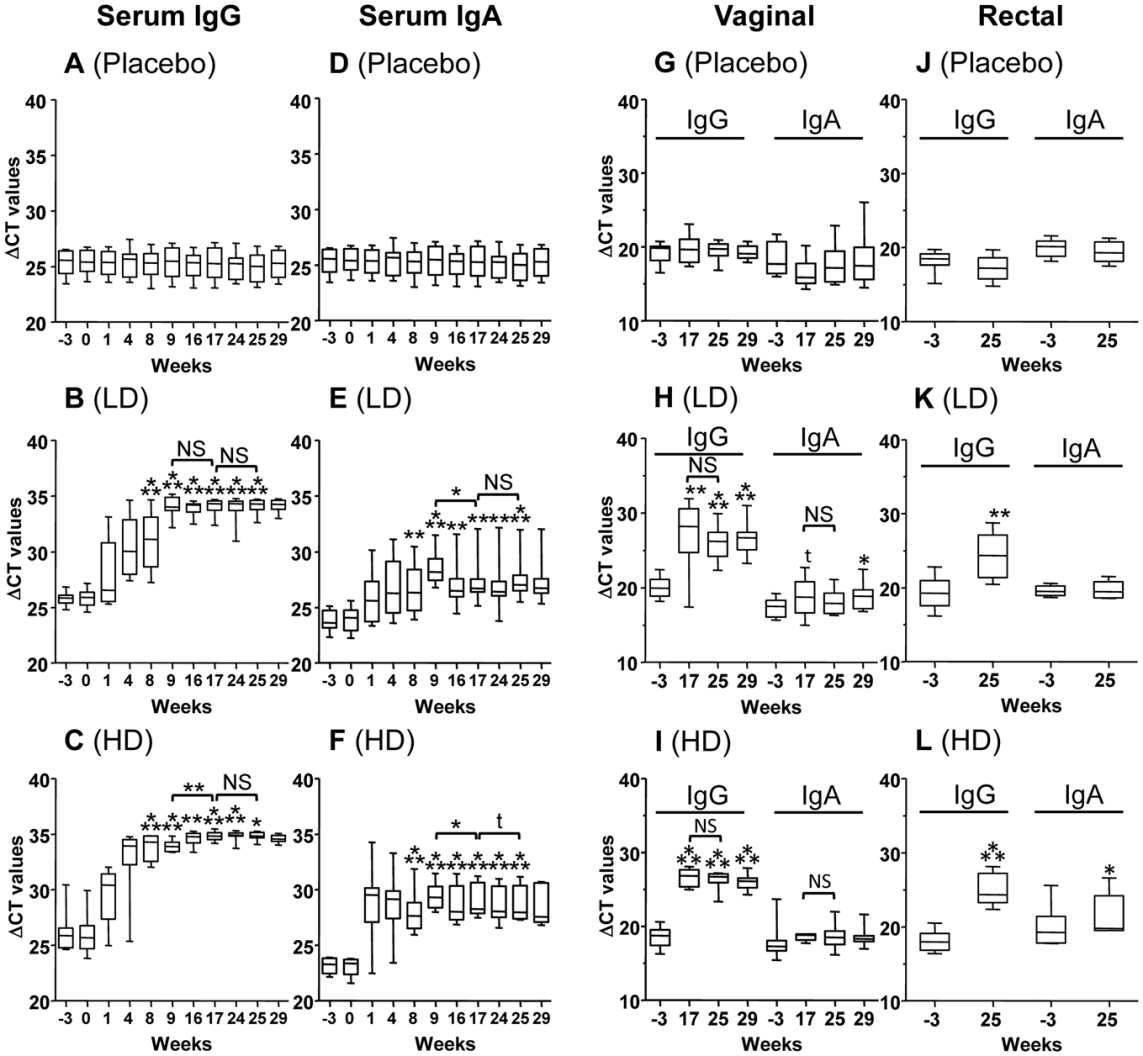
Serum and mucosal anti-P1 specific antibody responses. The presence of P1-specific IgGs and IgAs was measured by Imperacer® and data are presented as the mean ΔCT value from pre-immune (Baseline/week −3) and immune samples (week 0 to week 29). Panels A, D, G and J are for the placebo, Panels B, E, H and K are for the LD, Panels C, F, I and L are for the HD. To determine if ΔCT values of immune samples were significantly increased, respective to pre-immune samples (week −3), p values for serum samples were calculated for weeks 8, 9, 16, 17, 24, 25, and for vaginal samples weeks 17, 25 and 29. For determining if the injected vaccine had a boost effect, respective to the previous vaccination, p values were also estimated between weeks 9–17 (2^nd^ versus 3^rd^ injection) or 17–25 (3^rd^ versus 4^th^ injection): *0.01<p<0.05, **0.001<p<0.01, ***p<0.001. NS (not significant), t (trend), LD (low dose), HD (high dose). Whisker 10–90% percentile with minimum, maximum and median. Normally distributed immunological data were tested by paired t-test whereas for non-normal distributed data, the Wilcoxon signed rank-sum test was used to compare pre- and post-vaccination results.

Based on Imperacer® ΔCT values shown in Figure 2, a clear increase of P1-specific IgGs and IgAs in serum was observed within one month (week 4) after the first vaccination (week 0) in both LD and HD vaccinated recipients, respective to pre-immune baseline (week −3). This increase was further boosted by the second intramuscular vaccination, as shown at week 9. In LD recipients, the third injection given intranasally at week 16 had no significant boosting effect on the serum P1-specific IgGs and IgAs. In HD vaccinees, the third injection had a clear boosting effect only on the serum P1-specific IgGs (p = 0.004). The fourth and last vaccination at week 24 had no significant impact on serum P1-specific IgG and IgA increase for both groups.

The mucosal responses in both LD (Figure 2H, p = 0.001) and HD (Figure 2I, p<0.001) vaccinated subjects showed a clear increase of P1-specific IgGs in vaginal samples at week 17, as compared to pre-immune vaginal samples (Baseline/week –3), while the fourth injection did not elicit a significant increase of the antibody levels. Except for week 29 in the LD group, vaginal P1-specific IgAs for both groups did not reach statistical significance. Rectal P1-specific IgGs were also detected in both LD (Figure 2K, p = 0.003) and HD (Figure 2L, p<0.001) vaccine recipients at week 25, while P1-specific IgAs could be measured only in HD vaccinees (Figure 2L, p = 0.034).

To avoid missing important mucosal observations either due to group analyses, high background values from pre-immune samples and/or IgA level fluctuations within the same person, responders were evaluated individually, using the 1.996 cut-off ΔΔCT value for weeks 17, 25 and 29, respective to baseline (Table 4 and Table 5). Regarding vaginal P1-specific IgGs in LD and HD groups, all subjects were positive 1 week after the fourth injection. In the placebo group, 1 out of 8 subjects tested positive at week 17 and another 1 out of 8 subjects at week 25. As opposed to constant detections of IgGs, detections of vaginal P1-specific IgAs were varying within subjects of the LD and HD, depending on the time points.

**Table 4:** Percentage of subjects with measured mucosal anti-P1 specific antibodies at each visit.

	Vaginal Antibodies						Rectal Antibodies	
	Week 17 (Visit 7)		Week 25 (Visit 9)		Week 29 (Visit 10)		Week 25 (Visit 9)	
	IgG	IgA	IgG	IgA	IgG	IgA	IgG	IgA
**Placebo**	12.5% (1/8)	0% (0/8)	12.5% (1/8)	0% (0/8)	0% (0/8)	12.5% (1/8)	12.5% (1/8)	12.5% (1/8)
**Low Dose**	87.5% (7/8)	50% (4/8)	100% (8/8)	12.5% (1/8)	100% (8/8)	37.5% (3/8)	83.3% (5/6)	16.7% (1/6)
**High Dose**	100% (7/7)	28.6% (2/7)	100% (7/7)	42.9% (3/7)	100% (7/7)	14.3% (1/7)	100% (7/7)	28.6% (2/7)

**Table 5:** Number of positive responses out of 3 visits.

	Vaginal Antibodies								
	Positive response in 1 out of 3 visits		Positive response in 2 out of 3 visits		Positive response in 3 out of 3 visits		Positive response in at least 1 out of 3 visits		
	IgG	IgA	IgG	IgA	IgG	IgA	IgG	IgA	
**Placebo**	25% (2/8)	12.5% (1/8)	0% (0/8)	0% (0/8)	0% (0/8)	0% (0/8)	25% (2/8)	12.5% (1/8)	
**Low Dose**	0% (0/8)	37.5% (3/8)	12.5% (1/8)	12.5% (1/8)	87.5% (7/8)	12.5% (1/8)	100% (8/8)	62.5% (5/8)	
**High Dose**	0% (0/7)	14.3% (1/7)	0% (0/7)	14.3% (1/7)	100% (7/7)	14.3% (1/7)	100% (7/7)	42.9% (3/7)	

**Table 4** is showing the number of positive subjects with detectable P1-specific antibodies for each scheduled visit, based on ΔΔCT values (ΔCT from immune-ΔCT baseline  =  ΔΔCT value; Positive if >1.996). **Table** **5** is indicating the number of positive P1-specific responses out of the three scheduled medical visits. Number of subjects per treatment group that has completed the study is indicated in parenthesis and the corresponding percentage value is indicated. Immune vaginal samples were collected at week 17 (1 week after 3^rd^ injection), week 25 (1 week after 4^th^ injection) and week 29 (5 weeks after 4^th^ injection). Immune rectal samples were collected at week 25. From visit to visit, the same subject can be positive or negative for the detection of mucosal antibodies, which renders difficult the data interpretation, as opposed to serum antibodies that remain stable over time.

The change from baseline was also calculated per visit (Table 5). Among the 15 subjects of the LD and HD, only two subjects (one per group) had vaginal samples positive for P1-specific IgAs in 3 out of 3 visits (week 17, 24 and 29), while the other subjects had 1 or 2 positive samples out of 3 visits. In LD, 5 out 8 subjects (62.5%) had at least one vaginal sample containing P1-specific IgAs at either week 17, 25 or 29. In HD, 3 out 7 subjects (42.9%) had at least one positive vaginal sample.

In rectal samples specific-IgGs were present in 83% and 100% of LD and HD subjects, respectively (Table 4). Rectal specific-IgAs were detected in 29% of HD subjects. The mean serum (Table 6) and mucosal (Table 7) P1-specific IgG and IgA concentrations are shown as indicative values. For serum, the mean P1-specific antibody concentrations are ranging from 380 to 605 ng/mL for IgGs and 30 to 53 ng/mL for IgAs, respective to high and low dose. Mucosal P1-specific IgG antibody concentrations at the end of the trial (Visit 10/week 29) are ranging from 1.73 to 5.96 ng/mL, while for IgA values are 0.07 to 0.22 ng/mL.

**Table 6:** Approximate serum P1-specific antibody concentrations.

IgG (ng/mL)								
	Baseline	V4/w8	V5/w9	V6/w16	V7/w17	V8/w24	V9/w25	V10/w29
Placebo (n)	8	8	8	8	8	8	8	8
Mean	1.3	1.2	1.4	1.2	1.5	1.2	1.1	1.3
SD	0.7	0.9	1.0	0.9	1.3	1.1	1.0	0.9
Median	1.1	0.9	1.0	0.9	0.9	0.9	0.8	0.9
Low Dose (n)	8	8	8	8	8	8	8	8
Mean	1.5	138	1443	466	564	574	617	605
SD	0.6	249	3233	221	297	405	499	459
Median	1.4	31	250	490	547	553	548	548
High Dose (n)	8	8	8	8	7	7	7	7
Mean	3.0	267	238	456	421	433	435	380
SD	5.2	193	142	207	115	157	158	148
Median	1.4	248	180	402	356	397	396	350

IgA (ng/mL)								
	Baseline	V4/w8	V5/w9	V6/w16	V7/w17	V8/w24	V9/w25	V10/w29
Placebo (n)	8	8	8	8	8	8	8	8
Mean	5.6	4.3	4.5	4.4	4.5	3.7	2.4	3.2
SD	12.6	8.6	9.1	9.2	9.2	7.7	4.0	6.1
Median	1.1	0.9	1.1	1.2	1.1	0.9	1	1.2
Low Dose (n)	8	8	8	8	8	8	8	8
Mean	2.2	20	40	37	53	57	51	53
SD	1.2	31	61	77	121	137	110	119
Median	1.9	7.3	17	10	11	9.4	13	11
High Dose (n)	8	8	8	8	7	7	7	7
Mean	0.9	32	48	38	38	32	36	30
SD	0.5	58	54	52	45	38	44	36
Median	1	10	27	13	14	12	12	9.3

**Table 7:** Approximate vaginal and rectal P1-specific antibody concentrations.

	Vaginal Antibodies (ng/mL)									Rectal Antibodies (ng/mL)			
	P1-specific IgGs				P1-specific IgAs				P1-specific IgGs			P1-specific IgAs	
	Baseline	V7 (w17)	V9 (w25)	V10 (w29)	Baseline	V7 (w17)	V9 (w25)	V10 (w29)	Baseline		V9 (w25)	Baseline	V9 (w25)
**Placebo (n)**	8	8	8	8	8	8	8	8	8		8	8	8
Mean	0.03	0.06	0.04	0.03	0.12	0.03	0.12	0.65	0.02		0.01	0.26	0.15
SD	0.02	0.08	0.03	0.03	0.17	0.06	0.24	1.67	0.02		0.01	0.22	0.09
Median	0.03	0.04	0.04	0.03	0.05	0	0.03	0.04	0.01		0	0.2	0.13
**Low Dose (n)**	8	8	8	8	8	8	8	8	6		8	6	8
Mean	0.08	15.66	3.67	5.96	0.06	0.29	0.13	0.22	0.07		1.58	0.19	3.22
SD	0.07	20.30	4.76	9.86	0.06	0.35	0.14	0.28	0.09		2.45	0.09	7.74
Median	0.06	6.12	2.17	2.81	0.05	0.14	0.08	0.13	0.03		0.55	0.15	0.26
**High Dose (n)**	8	7	7	7	8	7	7	7	8		7	8	7
Mean	0.01	2.65	2.06	1.73	0.14	0.05	0.09	0.07	0.01		1.30	0.49	1.18
SD	0.02	1.74	1.24	1.05	0.34	0.02	0.12	0.10	0.02		1.48	1.07	2.05
Median	0	1.96	2.02	1.71	0.01	0.05	0.03	0.07	0		0.72	0.11	0.19

### Cellular immune responses

Although the MYM-V101 vaccine formulation was primarily designed to trigger antibodies, it was estimated that it could potentially elicit also a Th1 response. Therefore PBMC were stimulated with P1 peptides and intracellular cytokine induction (IFN*γ*, TNFα?IL-2) was analyzed by FACS. No P1-specific induction of cellular immune response in CD4^+^ or CD8^+^ T cells and NK cells could be revealed (data not shown).

### Evaluation of anti-viral activities

Due to limited mucosal material, not all subjects could be tested. Based on the presence of P1-specific IgG and/or IgA antibodies and the available specimen volume, selected samples were tested for *in vitro* antiviral activities. The genetically engineered TZM-bl cell line was tested with the clade B laboratory strain SF162 and the primary strain QHO692.42, while the U87.CD4.CCR5 cell line was tested with the JFRL-140WT. No neutralization of HIV-1 was found for all samples tested (data not shown).

**Figure 3:**
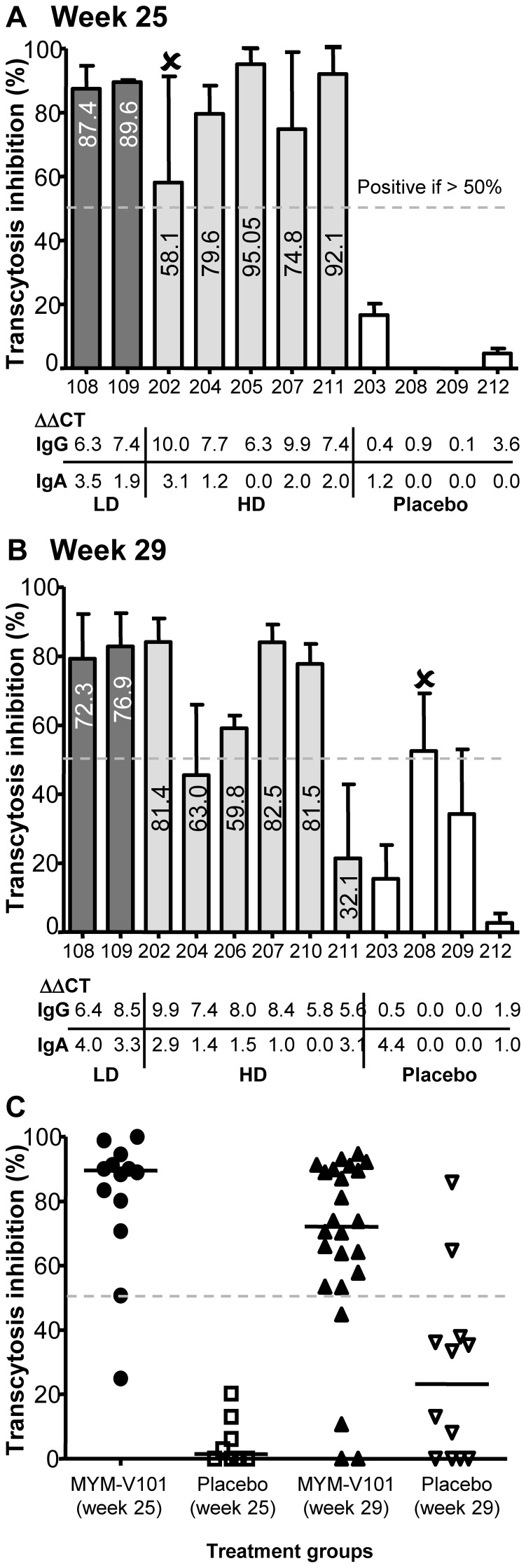
*In vitro* HIV-1 transcytosis inhibition by vaginal antibodies. Vaginal samples were collected from placebo and MYM-V101 vaccinated groups for exploratory work on transcytosis. Panel A (Visit 9/week 25) and B (Visit 10/week 29) are showing the measured mean values of transcytosis inhibition with standard deviations (histogram bars), with the indicated median values for LD and HD subjects within the bars. Percentages of transcytosis inhibitions were determined in three independent experiments. Samples were defined as positive when the transcytosis inhibitions were above 50% and reproduced in at least 2 experiments. Samples indicated by the (x) symbol means that they are negative for transcytosis, as they had only 1 measurement above 50% but the mean of the different experiments has generated a mean value above 50% (see method section). For each subject, the corresponding ΔΔCT value for P1-specific IgGs and IgAs is indicated: Samples with values above 1.996 are positive for the presence of specific antibodies. Low Dose (LD) dark grey bars, High Dose (HD) light grey bars and placebo with white bars. Panel C is showing all individual transcytosis data of each placebo and MYM-V101 subject from each representative experiment at week 25 and 29, allowing an overall qualitative comparison of the presence of transcytosis inhibition activity in vaginal samples. Placebo week 25 (opened square) compared with MYM-V101 week 25 (black circle), and placebo week 29 (opened triangle), as compared to MYM-V101 week 29 (black triangle).

Analyses on vaginal samples from the same subset of subjects were tested for their capacity to block HIV-1 transcytosis *in vitro* (Figure 3), with the mean percentage inhibition provided only as indicative value for a qualitative assessment. Vaginal samples of two out of two subjects of LD displayed 79–90% transcytosis inhibition at weeks 25 and 29. For HD at week 25, four out of five subjects were found positive (75–96%), while at week 29, four out of six subjects were positive (59–84%). Vaginal samples from the placebo group had no significant transcytosis inhibition. Some samples had no significant levels of IgA antibodies (ΔΔCT <1.996 cut-off), despite high transcytosis inhibition. However, if both P1-specific vaginal IgGs and IgAs are considered, the antiviral activity is consistently observed in 12 out of 15 samples (LD and HD).

## Discussion

Neutralizing antibodies and cytotoxic T cells play certainly an important role in eradicating or containing viral infection but they represent the “tip of the iceberg” of the arsenal potential of the immune system. Because HIV-1 is mainly acquired at mucosal sites, mucosal immune responses that interfere with HIV-1 attachment and migration across the mucosa, and promote viral clearance may contribute also to prevent sexual HIV-1 transmission. Protective mucosal antibodies could be elicited in the female genital tract by vaginal vaccination but this administration route poses several challenges and may require a mucosal adjuvant in order to induce an optimal immune response [82]–[85].

We have designed a prophylactic HIV-1 vaccine candidate that is potentially capable of protecting the initial sites of viral entry, especially the female genitals and the rectum, by inducing a mucosal humoral immune response without the need of local vaccination. The proposed MYM-V101 candidate HIV-1 vaccine was tested in a Phase I “Proof of Principle” to evaluate its safety and tolerability, when administered via intramuscular and intranasal routes in healthy young women.

The rationale for this immunization regimen was based on the postulate that intranasal priming may not be very efficient unless a potent mucosal adjuvant is added to the formulation. However, vaccination by intranasal route without adjuvant could work more efficiently if applied as a boost, following adequate priming via the intramuscular route. Although such immunization regimen may be less practical for medical care providers, it may minimize safety and regulatory concerns if mucosal adjuvant is absent from the vaccine. MYM-V101 can be considered as a safe vaccine that is well tolerated, when administered by the intramuscular (Table 2) and intranasal (Table 3) routes at the tested concentrations. No significant difference could be observed between the safety results of vaccinated groups and placebo recipients.

Very few HIV trials have tried without success to monitor mucosal antibodies during prophylactic vaccination [24], [25]. We may postulate that the detection assays employed at that time were not sensitive and robust enough for quantifying very low levels of specific mucosal antibodies. For our trial, we have developed the Imperacer®, a fast, robust and ultrasensitive detection assay [69]-[72] already used by pharmaceutical industries for other applications, which is suitable for detecting specific low mucosal antibody concentrations in the range of pg to ng/mL. The assay is fundamentally based on ELISA but the antibody-enzyme conjugates are replaced by antibody-DNA conjugates, which can be amplified by PCR [69], [70]. This technique was validated during clinical development and it allows reliable detection of antibodies in serum and mucosal samples, offering also the main advantage of consuming few μL.

The vaccine MYM-V101 has induced P1-specific IgG and IgA responses in serum already within 4 weeks after the first vaccination (Figure 2). There is a clear benefit of the first two intramuscular injections for triggering the systemic humoral response. The third vaccine dose given intranasally elicited only a significant antibody boost of serum IgGs in the HD group. No clear effect of the third dose was seen on serum IgA levels and no booster effect of the fourth dose could be demonstrated. Currently, it cannot be excluded that the third and fourth vaccine doses may have a beneficial effect by improving the affinity and/or the antiviral capacity of the elicited antibodies, as well as improving the memory response. However, this needs to be investigated further.

One month after the last vaccination, vaginal and rectal P1-specific IgGs were detected in almost all vaccinees. The P1-specific IgGs found in mucosal samples may be strictly derived from local production but can also partially have a circulatory origin. Due to unexpected high pre-immune vaginal reactivity toward the P1 antigen (high pre-immune ΔCT value) observed in some subjects, the analysis per group did not reveal a significant increase in mucosal P1-specific IgAs in immune samples. Unspecific binding is always possible but the observed reactivity could also be due to specific binding detected by the ultrasensitive Imperacer® technique. We may postulate that pre-immune sample reactivity could be related to: i) The presence of auto-antibodies against self proteins that cross-react with HIV-1 gp41 motifs, since HIV-1 is known to share several human protein homologies [86]–[88]; or ii) Subjects were exposed to HIV-infected individuals prior to this study and have developed specific mucosal anti-gp41 antibodies, while remaining seronegative, as reported in HIV exposed-seronegative (HESN) individuals [45]. The latter hypothesis is very unlikely, considering the “profile” of our volunteers but we cannot exclude this possibility. More investigations would be needed to further explore these possibilities but it was not the objective of this exploratory study conducted on limited number of subjects.

When ΔCT values of immune samples were individually considered and compared to their corresponding pre-immune ΔCT values, many subjects taken separately had a ΔΔCT value (ΔCT immune – ΔCT pre-immune) above the 1.966 cut-off, suggesting a net increase of mucosal P1-specific IgAs after vaccination with MYM-V101 (see Tables 5). P1-specific IgAs were detected in vaginal samples of 63% (LD) and 43% (HD) of the subjects with at least one positive mucosal sample out of three. We have noticed that detection of vaginal P1-specific IgAs seems to pose a big challenge due to the variation that occurs over time within a single individual. This may be explained in part by the hormonal and menstrual cycle fluctuations already reported [83], [84], which could influence the amount of IgAs already low in the women vagina, while IgAs in the endocervix are more abundant and easier to detect. The low level of IgAs, respective to IgGs was expected in the vaginal secretion but the low frequency of positive samples may also point towards a technical problem, as IgA is the dominant antibody isotype in the lower intestinal tract. The current antibody detection methodology and rectal harvesting technique will require further optimization for optimal IgA detection.

To alleviate the study burden for the participants, the collection of mucosal samples on week 9 (one week after the second intramuscular injection) was abandoned. Therefore, the benefit of the third injection (first intranasal administration) on mucosal antibodies is difficult to appreciate at this stage. Meanwhile, it is known that intranasal vaccination may solicit distant mucosal and systemic immune responses [82], [89], depending on the induction of specific sets of homing receptors during the interaction on T and B cells with mucosal dendritic cells [90], [91]. However, activation of the mucosal responses at the vaginal and intestinal levels by the intramuscular route, using non-replicative vectors like virosomes might be more an exception than the rule. In an upcoming clinical trial, additional mucosal samples will be collected to clarify the respective contribution of the intramuscular and intranasal route for the induction of mucosal immunity. The available data allows us to conclude that MYM-V101 has successfully induced specific mucosal antibodies.

At the end of the study (week 29, Tables 6 and 7), the mean total P1-specific antibody concentration in vaginal samples (LD: 6.18 ng/mL and HD: 1.6 ng/mL) is at least 100-fold lower than serum samples (LD: 658 ng/mL and HD: 410 ng/mL). These low mucosal antibody concentrations preclude their successful testing in current *in vitr*o neutralization assays, which were initially developed for serum samples and generally require at least 200 ng/well of neutralizing specific antibodies [78]. Currently, we cannot exclude the presence of low levels of neutralizing antibodies in vaginal or rectal fluids and antibody purification might be necessary for detecting them. Meanwhile, adding purification steps for specific IgGs and IgAs could introduce risks of losing or affecting the quality of samples and this approach might not be easily applicable to clinical trials evaluating thousands of samples. It might be more realistic to improve the *in vitro* neutralization assay sensitivity by at least 100-fold, ideally requiring less than 1 ng/well of specific antibodies for reliable testing of low amount of unpurified mucosal samples.

Although few ng/mL of specific mucosal antibodies might be perceived as a very low antibody concentration in the vaccine field, respective to serums that generally contain μg/mL of specific antibodies, it still represents billions of antibodies per mL of vaginal secretion. Furthermore, it is likely an underestimation, considering the contribution of the mucosal antibodies also trapped in the mucus or located in the *lamina propria* underneath the mucosal tissue that could not be estimated from the collected secretion samples of this study.

Transcytosis inhibition was investigated only for vaginal samples, as our previous studies had shown that circulatory antibodies could not inhibit HIV-1 transcytosis [27] and rectal samples were too limited. Although the transcytosis assay has been standardized to some extent in various laboratories, variations are inherent to *in vitro* cell-based assays and very often, experiments are conducted at least twice for confirming the *in vitro* observations. Transcytosis assay was developed to obtain robust data (qualitative observation) but was not formally qualified, as it is done for bio-analysis of the active pharmaceutical ingredient. Acting as a candidate biomarker for induction of functional mucosal immunity, transcytosis assay may provide reliable data for detecting antiviral activities, such as antibodies interfering with HIV-1 passage across *in vitro* cell monolayer, mimicking the mucosal epithelium found in the endocervix or intestinal tract. Eighty percent of the vaginal samples were inhibiting HIV-1 transcytosis (Figure 3), as opposed to placebo samples that had no activity. For some mucosal samples, discrepancies were found between the observed transcytosis inhibition that was shown to be mainly dependent on IgAs [27] and the absence of mucosal IgAs detection. We are postulating that the current antibody detection assay may not detect the full IgA antibody repertoire and further optimization is required. IgGs could also act in synergy with IgAs for optimal antiviral activities for blocking early steps of HIV transfer and infection at the mucosal sites [92]. We postulate that a prophylactic HIV-1 vaccine as MYM-V101 that elicits mucosal anti-gp41 antibodies in the range of 5–10 ng/mL or more (at least 10^10^ molecules of antibodies/mL of secretion) could efficiently block mucosal HIV-1 acquisition from semen of HIV-1 acutely infected men, which generally contains only 10^2^ to 10^5^ cell-free infectious particles or thousands of HIV-infected cells [64], [93], [94].

The mucosal immune system of the male reproductive tract in human [95], [96] and macaque [97] has been studied only recently. These studies have revealed the presence of antibodies but also of HIV-1 target cells like dendritic cells, Langerhans cells and T lymphocytes, explaining why the penile foreskin, inner foreskin mucosal epithelium, glans and urethra, are potential sites of HIV-1 acquisition in men. These observations are suggesting that vaccines eliciting mucosal immunity reaching the male reproductive tract could protect men from acquiring HIV. In subsequent human trials, the vaccine safety and immunogenicity will also be tested in men, in parallel to women for monitoring vaccine-induced antibodies in the genital tract of both genders.

Virus-like particles (VLPs) and enveloped VLPs such as virosomes harbour antigens at their surface, which are seen as repetitive motifs that are efficiently recognized by B cell surface antibodies, leading to their activation. Most VLP-based vaccines are employing adjuvant such as alum salts and Toll-like receptor agonists, while influenza-virosomes as enveloped VLPs are used as stand-alone products. Therefore, comparing the immune responses induced by both adjuvanted VLP and virosome is difficult, especially if pre-existing immunity impact the vaccine-induce immune response. Most people have natural pre-existing immunity against influenza, comprizing both humoral and cellular immunity (CD4^+^ and CD8^+^ T cells), which can be beneficial to vaccination with influenza-virosomes [57]. Influenza-specific antibodies were shown to bind to virosomes and facilitate their delivery inside the endosomes of antigen presenting cells, favouring a Th2 response characterized by a robust antibody production, as observed in our Phase I study.

Antigen cross-presentation [98] leading to CTL induction is also possible with virosomes. However, the immunodominant human CTL epitopes ERYLKDQQL and CSGKLIC in the HXB2 gp41 ectodomain (HIV gp41 CTL epitope data base) are absent from our P1 peptide used for vaccination, which renders unlikely the induction of CD8^+^ T cell response. Meanwhile, it was important to verify this aspect and *in vitro* stimulation of PBMC with P1 peptide followed by intracellular cytokine staining was unable to reveal P1-specific CD4^+^ or CD8^+^ Th1 response, although the threshold sensitivity of this method might be too weak for detecting low level of cell-mediated response.

Both IgG or IgA antibodies may bind to their respective Fc receptor [99] and trigger various viral clearance mechanisms through Fc-mediated effector functions: Complement activation [100], antibody-mediated phagocytosis [101], [102], or engagement of antibodies with NK cells, neutrophils or macrophages that leads to ADCC [103]–[107]. The potential protective role of ADCC induced by prophylactic vaccination was already reported in the vaginal tract of vaccinated NHP with virosome-gp41 [27] and serum of vaccinated subjects with ALVAC and AIDSVAX that developed antibodies toward the gp120 [22]. Presence of HIV specific mucosal antibodies capable of ADCC activity were also shown to reduce risk of vertical transmission to breastfed infants of HIV-1 positive women [108]. Therefore, ADCC activity may represent an important arm of the immune defense against HIV-1. Due to insufficient material, such binding non-neutralizing antibodies with ADCC activity could not be tested in this Phase I study but they will be part of future clinical investigations.

The idea of inducing IgAs as important and complementary players with IgGs in mucosal and systemic protection represents a new avenue in the HIV-1 vaccine field. It will be interesting to determine in the future if blood and mucosal antibodies toward the same antigen have different or similar epitope specificities, antibody isotype and how this may impact the antiviral functions in the systemic and mucosal immune compartments.

This Phase I represents the “Proof of Principle” that it is possible to elicit both specific IgGs and IgAs in circulation, as well as in vaginal and intestinal mucosal tissues, using virosome-based vaccines. The current study did not allow a broad coverage of potential antiviral activities. With recent *in vitro* immune assay developments, a spectrum of innovative immuno-monitoring investigations will be explored and future clinical trials will benefit from cutting edge ultrasensitive antibody detection assays combined with new functional antibody assays for evaluating various antiviral activities. These investigations will provide new insights regarding protective immune mechanisms from blood and/or mucosal compartments, as well as new potential surrogate markers of protection. In this study, the presence of vaccine-induced mucosal anti-gp41 antibodies with antiviral activities has confirmed the previous results in NHP [27], which further supports the approach of gp41-virosome as a promising vaccine strategy to induce mucosal antibodies for reducing sexually-transmitted HIV-1.

## Acknowledgments

We thank all the volunteers for their participation to the study. Special thanks to our main shareholders and Prof. Stanley Plotkin and Prof. Marc Girard for their precious long-term support and advice. We thank Mark Spengler and Jan Detmers (Chimera Biotec GmbH) for their strong involvement in the Imperacer® assay development. We are also grateful to Fabienne Anjuère and Selma Bekri (INSERM UMR 634, Faculté de Médecine Pasteur, Nice, France) who have developed and validated a B-cell ELISPOT assay which gave us important highlights on the adequate time window for mucosal sampling which would be necessary to further access gp41-derived antigen specific response in the future. We also thank the team of Lucia Lopalco for performance of neutralization assays, as well as Anne-Sophie Drillet and Danila Tudor from the laboratory of Morgane Bomsel for conducting transcytosis assays. We also thank Donald Forthal for preliminary testing of some samples in ADCVI assays. We thank also all the other persons involved in the clinical development, data management and reviewing process that were not mentioned.

## Supporting Information

Checklist S1 **CONSORT Checklist.**
(PDF)

Protocol S1 **Trial Protocol.**
(PDF)
