# Supplementary material for: Randomized Phase I: Safety, Immunogenicity and Mucosal Antiviral Activity in Young Healthy Women Vaccinated with HIV-1 Gp41 P1 Peptide on Virosomes
Source: PLoS One. 2013 Feb 20;8(2):e55438. doi: 10.1371/journal.pone.0055438 (PMC3577797; doi:10.1371/journal.pone.0055438)
Supplement: Protocol S1 — Trial Protocol. (PDF) [file pone.0055438.s002.pdf]

Clinical Trial Protocol Amendment 02

	Clinical R&D	**Nonproprietary name:**	MYM-V101
**Version:**	1.0	**Date:** 29-Sep-2009	**Status**: Final
**Title:**	A dose escalating Phase I study, double-blind, randomized, placebo-controlled for examining the safety and tolerability to a prophylactic HIV-1 vaccine called MYM-V101, administered intra-muscularly in combination with intra-nasal administrations to healthy female subjects.		
**Clinical Trial No:**<br>**Amendment Code:**	MYM-V101-CT08-101<br>MYM-V101-CT08-101_CTP-AM02	**Clinical Phase:**	I<br>“Proof of Principle”
**EudraCT No:**	2008-007306-10		

Summary:

This is a first-in-man, dose escalating Phase I study, single center, randomized, double-blind, placebo-controlled to examine the safety of and tolerability to a virosomes-based vaccine containing P1 peptides (product MYM-V101) derived from the gp41 protein of HIV-1 (Human Immunodeficiency Virus type 1), administered intra-muscularly (i.m.) in combination with intra-nasal (i.n.) administrations. The proposed virosomes-based vaccine has been designed for elicitating the antibody response against the HIV-1 gp41 protein for potentially preventing HIV infection.

The study population will consist of 24 healthy adult female subjects, equally distributed over 2 Panels (Panels 1 and 2). In a blinded mode for each Panel, 8 subjects will receive the active vaccine MYM-V101 and 4 subjects will receive the placebo product MYM-IRIV, which are virosomes alone without P1 peptides. Randomization will be done per Panel. Treatment in the panels will be administered in a dose escalation design as follows:

Panel 1. Subjects will receive 2 i.m. injections of MYM-V101.1 (10 µg/0.5 mL), administered at Weeks 0 and 8, followed by 2 i.n. administrations of MYM-V101.4 (10 µg/0.2 mL), administered at Weeks 16 and 24. The subjects will receive the second i.m. administration (Visit 4) once all subjects have completed Visit 2, and only if the first i.m. vaccination was found safe and tolerable after a safety review on the data of the first week after vaccination. The second i.n. administration (Visit 8) will only be given when all subjects have completed Visit 7, and after a safety review on the data of the first week after i.n. administration. No more than 5 subjects will start vaccination at the same day during Visit 1. Subject monitoring will end 5 weeks after the last vaccination.

	**Panel 2**. Subjects will receive 2 i.m. injections of MYM-V101.2 (50 µg/0.5 mL), administered at Weeks 0 and 8, followed by 2 i.n. administrations of MYM-V101.3 (50 µg/0.2 mL), administered at Weeks 16 and 24. Panel 2 will start treatment after all subjects in Panel 1 have completed Visit 2, and only if the 10 µg i.m. dose was found to be safe and tolerable. Subjects in Panel 2 will receive the second i.m. administration (Visit 4) once all subjects in Panel 2 have completed Visit 2, and only if the first high dose i.m. vaccination was found safe and tolerable after a safety review on the data of the first week after i.m. vaccination. Subjects in Panel 2 will receive the first i.n. administration (Visit 6) once all subjects in Panel 1 have completed Visit 7 (10 µg i.n.) and all subjects in Panel 2 have completed Visit 2 (50 µg i.m.), and only if the aforementioned vaccinations were found to be safe and tolerable. Subjects in Panel 2 will receive the second i.n. administration (Visit 8) once all subjects in Panel 2 have completed Visit 7, and only if the first high dose i.n. vaccination was found safe and tolerable after a safety review on the data of the first week after i.n. vaccination. No more than 5 subjects will start vaccination at the same day during Visit 1. Subject monitoring will end 5 weeks after the last vaccination.<br><br>Furthermore, the stability of the IMPs will be monitored in real time and cumulative P1 antigen associated degraded mass will be calculated throughout the study. If necessary, dose adjustments may follow.<br><br>Occurrence of local and systemic adverse events (AEs) will be assessed until 2 hours after each vaccination. Subjects will be asked to daily record solicited AEs in diary cards from Day 1 to Day 7 after each vaccination, which will be reviewed by the study personnel on the next visit to the clinical unit. Other diary cards will be used from Week 2 to Week 8 after each vaccination to record AEs, if applicable. Occurrence of clinically significant hematological and biochemical abnormalities will be followed throughout the trial. Safety and tolerability evaluations will be continuously recorded.<br><br>For the secondary objective of the study, serums will be taken to assess for the presence of blood anti-P1 antibodies (anti-HIV-1 gp41) at several time points throughout the trial. Ancillary study will also be conducted with vaginal and rectal secretions to assess for the presence of mucosal IgA and IgG anti-P1 antibodies. Peripheral blood lymphocytes (PBL) samples are also required for studying the mucosal B cell phenotype evolution during vaccination. Mucosal and blood antibodies induced by the MYM-V101 vaccine might be assessed for their capacity to block HIV-1 infection and transcytosis *in vitro*.
**Treatment:**	4 vaccinations every 8 weeks (excl. Screening and Follow-up)

Contact Details

**Sponsor**	
Sponsor<br>Scientific writing/review of the protocol	Dr. Sylvain Fleury<br>Mymetics Corporation<br>4, route de la Corniche<br>1066 Epalinges, Switzerland<br>Phone: +41-21.653.24.71<br>Mobile: +41-76.560.85.60<br>Fax: +41-21.653.24.73<br>E-mail : sylvain.fleury@mymetics.com
Scientific writing/review of the protocol	Dr. Anick Chalifour<br>Mymetics Corporation<br>4, route de la Corniche<br>1066 Epalinges, Switzerland<br>Phone: +41-.21.653.24.72<br>E-mail: anick.chalifour@mymetics.com<br><br>Dr. Morgane Bomsel<br>Institut Cochin<br>Entrée muqueuse du VIH et Immunité muqueuse<br>Département de Biologie Cellulaire<br>22, rue Méchain<br>75014 Paris, France<br>Phone: +33-1.40.51.64.97<br>Fax: +33-1.40.51.64.54<br>E-mail: morgane.bomsel@inserm.fr
**Contract Research Organisation**	
Contract Research Organisation (CRO)<br>Sponsor's legal representative in the EU	Kinesis Pharma BV<br>Lage Mosten 29<br>4822 NK Breda, The Netherlands<br>Phone: +31-76.54.80.666<br>Fax: +31-76.54.21.777
Trial manager,<br>author of the protocol	Dr. Marieke van den Dobbelsteen<br>Kinesis Pharma BV<br>Lage Mosten 29<br>4822 NK, Breda, The Netherlands<br>Phone: +31-76.54.80.628<br>Fax: +31-76.54.21.777<br>E-mail: Marieke.vandenDobbelsteen@Kinesis-Pharma.com

**Clinical study site**	
Clinical study site	CEVAC<br>De Pintelaan 185<br>9000 Gent<br>Belgium<br>Phone: +32-93.32.20.68<br>Fax: +32-93.32.22.85
Principal Investigator,<br>Head of clinical site	Prof. Dr. Geert Leroux-Roels<br>Faculty of Medicine of the University of Gent<br>De Pintelaan 185<br>9000 Gent<br>Belgium<br>Phone: +32-93.32.20.68<br>Fax: +32-93.32.22.85<br>E-mail: Geert.LerouxRoels@ugent.be
Investigator	Cathy Maes<br>Julie Willekens<br>CEVAC<br>De Pintelaan 185<br>9000 Gent<br>Belgium<br>Phone: +32-9-332 20 68<br>Fax: +32-9-332 22 85<br>E-mail: Cathy.Maes@uzgent.be<br>Julie.Willekens@ugent.be

An up-to-date version of the contact details of sponsor, laboratories and other third parties will be available in the investigator site file (ISF) and trial master file (TMF).

Table of Contents

1 Glossary 9
2 Flowchart Panels 1 and 2 11
3 Introduction 14
3.1 HIV-1 Vaccine Overview 14
3.2 New HIV-1 Vaccine Approach: Mucosal Protection 15
3.3 HIV-1 Gp41: The Vaccine Antigen 16
3.4 Virosomes: A Safe Vaccine Delivery System 17
3.4.1 Pre-clinical Studies with Gp41 Antigens 18
3.4.2 Toxicology Studies with MYM-V101 21
4 Objectives 23
4.1 Primary Objective 23
4.2 Secondary Objective 23
4.3 Ancillary Objectives 23
5 Methods 23
5.1 Trial Design 23
5.1.1 Overview of Trial 23
5.1.2 Discussion of Trial Design and Selection of Dose(s) in the Trial . 25
5.2 Trial Population 27
5.2.1 Sample Size 27
5.2.2 Inclusion Criteria 27
5.2.3 Exclusion Criteria 28
5.2.4 Prohibitions and Restrictions 29
5.2.5 Removal of Subjects from Therapy or Assessment 29
5.2.6 Data Review Meetings 30
5.2.7 Definition of Dose Limiting Toxicity and Maximum Tolerated Dose 31
5.2.8 Trial Termination Criteria 32
5.3 Treatment 32
5.3.1 Identity of Investigational Products 32
5.3.2 Packaging and Labeling 33
5.3.3 Randomization 33
5.3.4 Dosage and Administration 33
5.3.5 Blinding and Unblinding 35
5.3.6 Drug Accountability 35
5.3.7 Storage 35
5.3.8 Compliance 36
5.3.9 Prior and Concomitant Therapy 36
5.4 Assessments 37
5.4.1 Timing of Assessments 37
5.4.1.1 Initial Subject Characteristics 37
5.4.1.2 Treatment Period 38
5.4.1.3 Follow Up 40
5.4.1.4 Early Withdrawal 40
5.4.2 Efficacy Evaluations 41
5.4.2.1 Immunological Monitoring 41

5.4.2.1.1 Immunological Monitoring on Serum....41
5.4.2.1.2 Immunological Monitoring on Secretions....42
5.4.2.2 Sample Collection and Handling....43
5.5.2.2.1 Secretion Samplings....43
5.4.3 Safety Evaluations....44
5.4.3.1 General....44
5.4.3.1.1 Adverse Event Definitions....44
5.4.3.1.2 Classification of Intensity of Adverse Events....45
5.4.3.1.3 Classification of Causality of AEs to the Investigational Product 45
5.4.3.1.4 Reporting of AEs....46
5.4.3.1.5 Checks for AEs....47
5.4.3.2 Risks Related to the Participation in the Clinical Trial....47
5.4.3.2.1 Procedure Related Risks....47
5.4.3.2.2 Risks Related to the Investigational Vaccine....48
5.4.3.3 Specific Toxicities....48
5.4.3.3.1 Occurrence of Local and Systemic AEs....48
5.4.3.3.2 Occurrence of Clinically Significant Hematological and Biochemical Abnormalities....49
5.4.3.4 Clinical Laboratory Tests....50
5.4.3.4.1 Haematology....50
5.4.3.4.2 Biochemistry....50
5.4.3.4.3 Additional Assessments....50
5.4.3.4.4 Urinalysis....50
5.4.3.5 Cardiovascular Safety....51
5.4.3.5.1 Vital Signs....51
5.4.3.5.2 ECG....51
5.4.3.6 Physical Examination....51
5.5 Statistical Methods Planned and Determination of the Sample Size....51
5.5.1 Determination of Sample Size....51
5.5.2 Statistical Analyses....52
5.5.2.1 Initial Subject and Disease Characteristics....52
5.5.2.2 Efficacy Analyses....52
5.5.2.2.1 Immunological Monitoring....52
5.5.2.3 Safety....52
5.5.2.3.1 Occurrence of Local and Systemic AEs....52
5.5.2.3.2 Adverse Events....52
5.5.2.3.3 Clinical Laboratory Tests....52
5.5.2.3.4 Cardiovascular Safety....53
5.5.2.3.5 Other Safety Parameters....53
6 Trial Closure Considerations....53
7 Trial Materials....53
7.1 Investigational Products....53
7.2 Trial Documents....54
7.3 Participation Cards....54
7.4 Source Data....54

7.5 Case Report Forms 55
7.6 Subject Identification Code List & Subject Screening and Enrolment Log, and CRF Log 56
7.7 Archiving 56
8 Confidentiality 56
9 Reporting and Publication 57
9.1 Reporting 57
9.2 Publication 57
10 Authorities/Ethics 57
10.1 Regulatory Authorities 57
10.2 Independent Ethics Committee 57
10.3 ICH-GCP Guidelines 58
10.4 Subject Information and Informed Consent 58
10.5 Privacy of Personal Data 58
11 Financing and Insurance 59
11.1 Financial Disclosure 59
11.2 Indemnification 59
11.3 Insurance 59
12 Data Quality Control/Assurance 59
13 References 60
14 Addenda 62
14.1 Addendum 1: Cardiovascular Safety – Abnormalities 62
14.2 Addendum S2: Vaccination Proposition during Menstrual Cycle. 63

1 Glossary

AE	Adverse Event
AIDS	Acquired Immune Deficiency Syndrome
ALT	Alanine Aminotransferase
ASC	Antibody-Secreting Cells
ARV	Anti Retroviral
ASR	Annual Safety Report
AST	Aspartate Aminotransferase
BMI	Body Mass Index
CD	Cellulair Determinant
CRF	Case Report Form
CTL	Cytotoxic T Lymphocyte
CVL	Cervico Vaginal Lavage
DBP	Diastolic Blood Pressure
DCF	Data Correction Form
DLT	Dose Limiting Toxicity
ECG	Electrocardiogram
ELISA	Enzyme-Linked ImmunoSorbent Assay
FU	Follow-up
GCP	Good Clinical Practice
GLP	Good Laboratory Practice
GMP	Good Manufacturing Practice
GP	Glycoprotein
HA	Hemagglutinin
HAART	Highly Active Anti-Retroviral Therapy
HDL	High-Density Lipoprotein
HIV	Human Immunodeficiency Virus (can be type 1 or 2: HIV-1 or HIV-2)
HLT	Heat Labile Toxine
ICF	Informed Consent Form
ICH	International Conference on Harmonization
IEC	Independent Ethics Committee
Ig	Immunoglobulin
ILAS	Institute of Laboratory Animal Science and Chinese Academy of Medical Science
i.m.	Intra-muscular(ly)
i.n.	Intra-nasal(ly)
IRIV	Immunopotentiating Reconstituted Influenza Virosomes
ISF	Investigator Site File

LDL	Low-Density Lipoprotein
MCH	Mean Corpuscular Haemoglobin
MCHC	Mean Corpuscular Haemoglobin Concentration
MCV	Mean Corpuscular Volume
MedDRA	Medical Dictionary for Regulatory Activities
MPER	Membrane Proximal Ectodomain Region of the HIV-1 gp41
MTD	Maximum Tolerated Dose
MYM-V101	Clinical batch of the HIV-1 virosome-based vaccine containing the P1 peptides
MYM-IRIV	Placebo vaccine containing only IRIV (influenza-based virosomes without P1 peptides)
NA	Neuramidase
NIAID	National Institute of Allergy and Infectious Diseases (one department of the NIH)
NIH	National Institutes of Health
OTC	Over the Counter
P1	Peptide derived from the HIV gp41 sequence 649-684 of HXB2 clade B
PBL	Peripheral Blood Lymphocytes
pcMYM-V101	Pre-clinical batch of the MYM-V101 (virosomes-P1)
PR	Pulse Rate
QA	Quality Assurance
QC	Quality Control
QTcB	QT interval corrected for heart rate according to Bazett
rgp41	Recombinant gp41
RBC	Red Blood Cell
RT	Room Temperature
RT-PCR	Reverse Transcriptase Polymerase Chain Reaction
SAE	Serious Adverse Event
SBP	Systolic Blood Pressure
SHIV	Simian Human Immunodeficiency Virus
SIV	Simian Immunodeficiency Virus
ULN	Upper Limit of Laboratory Normal Range
UNAIDS	Joint United Nations Program on HIV and AIDS
WBC	White Blood Cell
WHO	World Health Organization

2 Flowchart Panels 1 and 2

	Screening Visit	Baseline Visit	Treatment period								Post-treatment Follow-up (FU) Visits		In case of Dropout
**Time of Visit**	≤ Week -5	Week -3[a]	Week 0		Week 4	Week 8		Week 16		Week 24	Week 25/ FU Visit (Week 1)	Week 29/ FU and Close Out Visit (Week 5)	Early With-drawal[b]
			**Visit 1**	**Visit 2**	**Visit 3**	**Visit 4**	**Visit 5**	**Visit 6**	**Visit 7**	**Visit 8**	**Visit 9**	**Visit 10**	
			Day 0[c]	Day +7[d]		Day 0[c]	Day +7[d]	Day 0[c]	Day +7[d]	Day 0[c]	Day +7[d]		
**Informed Consent Form (ICF) signature**	X												
**Demographic data**	X												
**Medical and surgical history & concomitant disease(s)**	X												
**Review of smoking habits, drug abuse and alcohol consumption**	X												
**Inclusion/exclusion criteria**	X	X											
**Physical examination , vital signs, incl. body temperature**	X	X	X	X	X	X	X	X	X	X	X	X	X
**Gynaecological examination incl vaginal swab**	X												
**Height, weight**	X											X	X
**ECG**	X	X									X	X	X
**Pregnancy test[e]**	X		X			X		X		X		X	X
**HIV-1 and -2 tests**	X		X			X						X	X
**Hepatitis A, B and C tests**	X											X	X

Flowchart continues on next page

FLOWCHART, CONT'D

	Screening Visit	Baseline Visit	Treatment period								Post-treatment Follow-up (FU) Visits		In case of Dropout
**Time of Visit**	≤ Week -5	Week -3[a]	Week 0		Week 4	Week 8		Week 16		Week 24	Week 25/ FU Visit (Week 1)	Week 29/ FU and Close Out Visit (Week 5)	Early With-drawal[b]
			Visit 1	Visit 2	Visit 3	Visit 4	Visit 5	Visit 6	Visit 7	Visit 8	Visit 9	Visit 10	
			Day 0[c]	Day +7[d]		Day 0[c]	Day +7[d]	Day 0[c]	Day +7[d]	Day 0[c]	Day +7[d]		
**Primary Objective (safety)**													
Moment of Council, Review of restrictions and precautions[f]	X	X	X	X	X	X	X	X	X	X	X	X	X
Concomitant therapies	X	X	X	X	X	X	X	X	X	X	X	X	X
AEs review and check		X	X	X	X	X	X	X	X	X	X	X	X
Diary cards			X	X	X	X	X	X	X	X	X	X	X
Laboratory safety assessments: blood biochemistry, hematology	X	X	X	X	X	X	X	X	X	X	X	X	X
Urinalysis	X	X	X		X	X		X		X	X	X	X
Vaccine administration[c]			X			X		X		X			
**Secondary Objective**													
Immune monitoring on serum		X	X[g]	X	X	X[g]	X	X[g]	X	X[g]	X	X	X
**Ancillary study**													
Immune monitoring on vaginal secretions[h]		X							X		X	X	
Immune monitoring on rectal secretions[h]		X									X		
Immune monitoring on PBL		X		X			X		X		X		

[a] Baseline pre-immune serum and secretion samples must be taken on Days 21 to 26 of the cycle prior to the cycle of first vaccination, but no later than 2 days prior to menses. In case of a short cycle, it should be taken at the latest two days before start of menses. This time window allows harvesting samples with minimal risk of antibody contamination originating from blood (coming from menses), and it avoids the mucus peak.

[b] At the time of discontinuation or as soon as possible within one week after discontinuation.

[c] Day of vaccination must match the woman's cycle between Days 1 to 5 after ovulation or mid-cycle. During this time window of the cycle, women are in a good immunocompetent state.

[d] Mucosal sampling shall take place on Days 21-26 of the menstrual cycle, but no later than 2 days prior to menses. Visit after vaccination must be done between Days 6 to 8 after vaccination. During this 3-day time window, specific B cells induced by vaccination can be detected in the blood and levels of specific mucosal antibodies should be detectable. Sampling before or after this time window may lead to poor result.

[e] Serum pregnancy test will be done at Screening Visit. For other time points, a urine pregnancy test will be done. These tests are only for women of childbearing potential.

[f] Reminding and questioning of subjects with regard to restrictions and precautions.

[g] Take blood samples before performing the vaccination.

[h] For each vaginal and rectal secretion sampling, two –four sequential harvestings (1 minute interval) with Weck-Cel® sponges is required for obtaining at least 60 µg of total mucosal antibodies. At Baseline, 2-4 sequential vaginal harvestings with Weck-Cel® sponges will be performed, while post-vaccination only 2 sequential vaginal harvestings will be performed. All rectal samplings will be performed in duplo, i.e. 2 Weck-Cel® sponges will be used, for details on rectal samplings, see section 5.4.2.2.1.

For vaginal secretions, it is IMPORTANT to perform the two - four samplings into two different regions:

- The first Weck-Cel® sponge(s) must be introduced into the upper part of the vaginal (avoid touching the exocervix);
- The second sampling(s) must be done in the lower part of the vagina. Because the first sampling may potentially cause light bleeding, it is important that the second sampling is done distant from the first sampling site to avoid blood contamination. Any contamination due to bleeding would render the material useless because mucosal antibodies would be contaminated by antibodies originating from the blood.

3 Introduction

UNAIDS (Joint United Nations Programme on HIV and AIDS (Acquired Immune Deficiency Syndrome) and the WHO (World Health Organisation) estimate that AIDS has killed more than 25 million people since it was first recognized in 1981, making it one of the most destructive pandemics in recorded history. Globally, between 33 and 46 million people currently live with HIV-1. In 2005, between 3 and 6 million people were newly infected and between 2 and 3 million people with AIDS died, of which more than half a million (570 000) were children. HIV-1 continues to disproportionately affect certain geographic regions (i.e., sub-Saharan Africa and the Caribbean) and subpopulations (i.e., women in sub-Saharan Africa, men who have sex with men, injection-drug users and sex workers).

Current drug treatments for HIV-1 focus on slowing or impeding the progress of the virus once it has infected the body's host cells. Treatments for HIV-1 infection consist of highly active anti-retroviral therapy (HAART). This has been highly beneficial to many HIV-1-infected individuals since its introduction in 1996. HAART neither cures the patient nor does it uniformly remove all symptoms. Despite this, many HIV-1-infected individuals have experienced remarkable improvements in their general health and quality of life, which has led to a large reduction in HIV-1-associated morbidity and mortality in the developed world. However, HAART achieves far less than optimal results, in some circumstances being effective in less than fifty percent of patients. This is due to a variety of reasons such as medication intolerance/side effects, prior ineffective anti-retroviral therapy (ART) and infection with a drug-resistant strain of HIV-1. However, non-adherence and non-persistence with ART is the major reason why most individuals fail to benefit from HAART. Anti-retroviral drugs are expensive and the majority of the world's infected individuals do not have access to medications and treatments for HIV and AIDS. Furthermore, HAART does not prevent the spreading of HIV-1 through people with undiagnosed HIV-1 infections. Research is still ongoing to improve current treatments.

3.1 HIV-1 Vaccine Overview

Only vaccines are thought to be able to halt or slow down the pandemic. Vaccines have less side effects, no daily treatments are required, and would be much cheaper, thus being affordable for developing countries. However, developing an HIV-1 vaccine is not an easy task because HIV-1 is a virus that constantly mutates and the few known potential HIV-1 vaccine antigens are heavily glycosylated or poorly immunogenic.

Over the last twenty years of research in HIV-1 vaccine field, hundreds of vaccine candidates have been investigated. During that period, scientists have pursued vaccines that induce an immune protection against infection events that take place only after HIV-1 transmission, meaning once the HIV-1 has crossed the mucosal tissues and has already infected cells. These vaccines were developed for triggering blood IgG antibodies or cytotoxic T cells (CTL), two important defense mechanisms. All these tested vaccines failed from inducing protective immunity for various reasons. Today, it is believed that blood IgG and CTL protections act on too late infection events, explaining in part why past vaccines could not protect. However, the failures of the past twenty years provide also a greater understanding of this complex disease, which has improved our knowledge in various research fields.

The scientific community is now seeking for vaccines that might target earlier transmission and infection events that take place during the first minutes or hours following exposure to HIV-1, therefore improving the chance of blocking or slowing down HIV-1 transmission. To develop an effective prophylactic vaccine against HIV-1, it is important to understand the biology of HIV-1 transmission. Viral infections occur through contact with contaminated blood or during unprotected vaginal or anal intercourse. Meanwhile, about 85% of HIV-1 infections are transmitted sexually, following mucosal tissue exposure to the virus. Indeed, semen and cervico-vaginal secretions may potentially transmit

HIV-1 to the gastrointestinal, anorectal and genitourinary tracts because they contain cell-free HIV-1 particles and numerous HIV-infected cells.

Once HIV-1 is in contact with the mucosal surface, the virus penetrates as fast as possible into the body by crossing through various mechanisms the superficial epithelium layer of mucosal tissues that act as a natural barrier. This passage across mucosal epithelium is considered as an early event that takes place before HIV-1 infection. Once inside the organism, HIV-1 has the possibility to infect various cells from the immune system such as CD4+ T lymphocytes, macrophages and dendritic cells, leading to massive infection and virus dissemination.

3.2 New HIV-1 Vaccine Approach: Mucosal Protection

As most of HIV-1 infections occur during sexual intercourse, protecting the main entry doors (genital and rectal compartments) against HIV-1 entrance might be best achieved by mucosal antibodies. These mucosal antibodies would act as the first line of defense against HIV-1 entry and infection events that take place over the first minutes or hours following mucosa exposure to the virus. Blood circulating IgG antibodies are also important as they might represent the second line of defense against viruses that have succeeded to enter the body, such as those reaching lymph nodes.

It is also important to remind that mucosal IgA and circulating IgG may have different inhibition potentials, another reason to induce both upon vaccination. Furthermore, antibody-secreting cells (ASCs) of IgG or IgA isotype may have a different niche distribution, likely due to the expression of different homing and chemokine receptors. For example, IgG ASCs from peripheral blood or gut-associated lymph nodes are mostly trafficking to lymphoid tissues, bone marrow and inflamed tissues. They poorly migrate to intestinal mucosal tissues due to the absence of chemokine receptor CCR9 or low expression of CCR10 and integrin $\alpha_4\beta_7$ (homing receptors). Vaccine designed for triggering the right homing and chemokine receptors pattern for mucosal migration might be more successful for preventing HIV-1 transmission.

To support the relevance of mucosal protection against HIV-1, it is interesting to look to subjects who remain seronegative, despite repeated exposure to HIV-1. Few studies have been done on these subjects. To explain the natural resistance to HIV-1 (excluding genetic mutations), the immune system of these subjects was carefully investigated. Overall, these subjects have maintained good levels of CD8+ T cells. But, the most astonishing aspect was the presence of mucosal IgA antibodies that specifically recognize the HIV-1 gp41 protein. More importantly, these antibodies were capable of neutralizing and inhibiting HIV-1 infection and transcytosis *in vitro*. From these observations, it has been postulated that mucosal anti-gp41 antibodies might represent an efficient defense mechanism for preventing HIV-1 transmission/infection.

Mymetics Corporation and its collaborators have launched in 2004 its HIV-1 vaccine program, trying to engineer an HIV-1 vaccine that could reproduce the mucosal immune responses developed by Mother Nature. This approach has been ignored or neglected for many years, likely due in part to a poor understanding of the mucosal immune system, which represents the biggest immune organ of the body. The vaccine strategy initiated by Mymetics might open the door to new prophylactic HIV-1 vaccines, which differs from the classical blood IgG protection strategy. Vaccines that aim at preventing HIV-1 entry at the primary entry sites across the mucosa, such as the reproductive-genital tracts and intestine/rectal mucosal tissue are representing new innovative approaches for efficiently fighting against this disease.

Because none of the previously tested vaccines were specifically designed for inducing mucosal antibodies as an efficient first line of defense, the interest for finding such an HIV-1 vaccine still resonates strongly among small biotech companies and academic laboratories, whose often overlooked

innovative mucosal approaches may now take center stage, as the search for a viable HIV-1 vaccine continues.

The ideal vaccine should offer a universal protection, but this represents a difficult task because HIV is not a single virus. There are two known HIV strains: HIV-1 and HIV-2. HIV-1 is more virulent, relatively easily transmitted and is the cause of the majority of HIV infections globally. HIV-2 is less transmittable and is largely confined to West-Africa. HIV-1 represents a family of different viruses which can be divided into three main groups. In the main group, the M-group, there are 9 different geographic clades recognized. Clade B is found in industrialized countries and represents about 12% of the world HIV-1-infected subjects. Clade C is found in Africa, India and China and represents about 60% of the world HIV-1-infected subjects. Vaccines should ideally protect against the most representative HIV-1 clades.

Although cell-mediated immunity is crucial for controlling and eradicating infection by many viruses, antibodies are pivotal in preventing and/or modulating the infection. Indeed, the main mode of protection that is induced by most marketed antiviral vaccines seems to be mediated by neutralizing antibodies.

3.3 HIV-1 Gp41: The Vaccine Antigen

The main viral envelope protein of HIV-1 is the mushroom-shaped glycoprotein 160 (gp160). Gp160 is composed of two parts: gp120 and gp41. Gp120 is the more exposed region and gp41 is the region that is anchored into the viral membrane (lipid envelope). The virus uses these proteins to attach to and infect cells. The only known antibodies capable of preventing HIV-1 infection are those recognizing gp120 or gp41. Gp41 is the smaller protein and shows relatively little variation across different HIV-1 subtypes, making this protein a suitable vaccine antigen for inducing a broader antibody protection.

Such broad protection might be achieved if the vaccine induces antibodies that recognize conserved sequences and/or three-dimensional structures (epitopes) on viral proteins that are shared among the different virus subgroups. Such potential viral targets do exist on the HIV-1 viral membrane proteins, i.e. 2F5 and 4E10 epitopes on HIV-1 gp41 protein. Only a few epitopes located on the HIV-1 envelop glycoproteins, gp120 and gp41, can induce neutralizing antibodies in natural infection and mediate protection against infection in passive immunization studies[1].

Therefore, identifying key epitopes as target for antibodies represent an important aspect of the vaccine solution. It is also crucial to determine if the vaccine antigen harboring key epitopes should be elaborated as a linear or tridimensional structure for optimal mimicry of the native pathogen'protein. The proposed HIV-1 vaccine of this study contains gp41 peptides with a very specific sequence derived from the HXB2, a clade B virus (X4 tropism). This sequence, once integrated into a lipid membrane is thought to adopt a conformation closely resembling the one present on the native gp41 protein. The reasons that led to the selection of this vaccine antigen are described below.

The membrane protein gp41 can be divided in 4 different ectodomain regions, each with subregions able to induce protective and unprotective immune responses. The membrane proximal ectodomain region (MPER) of the gp41 has attracted much attention in the field of HIV-1 vaccinology due to the following features: i) it is the target of two of the few broadly neutralizing IgG monoclonal antibodies against HIV-1, namely 4E10 and 2F5, ii) it has been shown to be important in the fusion process of the virus with the host cells and therefore in viral entry, iii) it has a highly conserved linear region among all HIV-1 clades[2], thereby providing a potential protein region that might elicit antibodies with a broad range of protection, and iv) it is also known for being recognized by mucosal IgA antibodies present in the vaginal secretions of some HIV-1-exposed but IgG seronegative women.

Although the MPER region is poorly immunogenic *in vivo,* likely due to the presence of non-immunodominant epitopes in this sequence, it has been suggested that adding to this antigen some lipid components that mimic HIV-1 membrane might improve its immunogenicity and the neutralization

capacity of antibodies toward this region. Therefore, vaccine strategies that combine the MPER derived antigens to lipid membrane, such as the virosomes (lipidic vesicle; see below) proposed in the present vaccine, may increase the chance to induce broadly neutralizing blood and mucosal antibodies.

The peptide 649-684 of the gp41 HXB2 clade B, the so-called P1 peptide, contains the MPER region. The sequence of this P1 peptide has been slightly modified to accommodate the industrial manufacturing process compatible with the virosome technology. The P1 peptide has been lipidated before getting incorporated into the virosome membrane, leading to the vaccine product MYM-V101 that will be investigated in this study. Using this antigen and vaccine engineering, the immune system is forced to recognize this protective region of the gp41. It is hypothesized that this vaccine might elicit broadly neutralizing blood (mostly IgG) and mucosal (mostly IgA) antibodies against the gp41 capable of recognizing different HIV-1 clades.

3.4 Virosomes: A Safe Vaccine Delivery System

Adjuvants (immune potentiators or immunomodulators) have been used for decades to improve the immune response to vaccine antigens. The incorporation of adjuvants into vaccine formulations is aimed at enhancing, accelerating and prolonging the specific immune response towards the desired response to vaccine antigens. Advantages of adjuvants include the enhancement of the immunogenicity of antigens, modification of the nature of the immune response, the reduction of the antigen amount needed for a successful immunisation, the reduction of the frequency of booster immunisations needed and an improved immune response in elderly and immunocompromised vaccinees.

There are six major categories of adjuvants, which can be made from mineral salts (ex. aluminium hydroxide or “alum”), oil emulsions (ex. MF59), particulates (ex. Virosomes, ISCOMS), microbial derivatives (ex. monophosphoryl lipid A “MPL”, CpG motif), plant derivatives (ex. saponin: QS21) or endogeneous immunostimulant molecules (ex. GM-CSF cytokine). However, very few adjuvants are approved for use in human vaccines. The most common are the mineral salt adjuvant called aluminium hydroxide and the particulate adjuvant called virosome based on the influenza membrane (from Berna/Crucell, Pevion).

Mymetics Corporation has formulated the HIV-1 vaccine adjuvanted with influenza-derived virosomes that act as synthetic carrier systems for the gp41 antigen delivery. Essentially, virosomes are virus-like particles and represent reconstituted empty influenza virus envelopes, lacking the genetic material of the native virus. Virosomes are stable round-shaped capsules made mostly of phospholipids and having an approximate diameter of 160 nm (0.00016 mm). These virosomes look on the outside like a virus; they mimic HIV-1 membrane and can efficiently deliver immunologically active substances, such as peptides and proteins, into the body to stimulate the immune system. The proposed virosomes-based HIV-1 vaccine does not contain genetic material or any lived or attenuated pathogens, it is strictly a non-replicative vaccine carrier mostly constituted of lipids, HIV-1 gp41 peptides and influenza membrane proteins called neuramidase (NA) and hemagglutinin (HA). Therefore, vaccination with virosomes-based P1 peptides (MYM-V101) shall not lead to HIV-1 and influenza infections.

In contrast to liposomes, virosomes contain functional influenza virus HA and NA intercalated in the phospholipid bilayer membrane. The unique properties of virosomes partially relate to the presence of biologically active influenza HA in their membrane. This viral protein not only confers structural stability and homogeneity to virosomes-based formulations, but it significantly contributes to the immunological properties of virosomes, which are clearly distinct from other liposomal and proteoliposomal carrier systems.

Properly reconstituted virosomes retain the cell binding and membrane fusion properties of the native influenza virus, mediated by the viral envelope glycoprotein HA. These functional characteristics of virosomes form the basis for their enhanced immunogenicity. First, the repetitive arrangement of HA

molecules on the virosomal surface mediates a cooperative interaction of the antigen with Ig receptors on B lymphocytes, stimulating strong antibody responses. In addition, virosomes interact efficiently with any cells or antigen-presenting cells, such as dendritic cells, and specifically deliver the antigens into the proper antigen processing compartments for optimal immune response.

Generally, antigens grafted at the surface of virosomes will be directed to the major histocompatibility class II pathways to favour antibody response, while encapsulated antigens will be delivered to the class I pathways to favour cytotoxic T lymphocytes. Finally, virosomes represent an excellent platform for inclusion of lipophilic adjuvants for further stimulation of vaccine immunogenicity. By virtue of these characteristics, virosomes represent a promising novel class of adjuvant for vaccines, which not only induce high virus-neutralizing antibody titers, but also prime the cellular arm of the immune system[3,4].

Virosomes are well-known and well-investigated carriers for vaccines. Virosomes-based vaccines are already available for vaccination against hepatitis A (Epaxal® marketed since 1994) and influenza (Inflexal® V marketed since 1997). These products are registered for human use in more than 42 countries and they were already administered to more than 30 million individuals.

Safety data on more than 2800 subjects vaccinated with the influenza vaccine Invivac®, which uses the same virosome technology as Inflexal®, were published[5]. It was concluded that the virosomal influenza vaccine is safe and has a favorable reaction profile. The most frequently reported local AEs from clinical studies were pain and redness at the site of injection and the most frequently reported systemic AEs were headache and fatigue. The symptoms were mostly mild in nature and resolved within 1-3 days after vaccination. The overall tolerability of the virosomal influenza vaccine is very high, with 97% of vaccinated subjects reporting none or only mild inconveniences following vaccination. Furthermore, these reported AEs are mostly observed during the first and second injections, while AEs are almost absent at the third injection. Safety results from Epaxal® vaccinated subjects also revealed that the safety profile of the virosomes-based vaccine was better than the conventional hepatitis A vaccines. Local reactions were mostly pain and redness at the site of injection and most frequently reported systemic reactions were fatigue, headache and arthralgia[6,7,8].

A phase I study with a virosomes-based malaria vaccine under development (Mymetics/Pevion Biotech), revealed that i.m. administration of the vaccine to 46 subjects resulted only in pain as a local AE in 24% of the subjects, which was of mild intensity in 62.5% of the cases and moderate in 37.5%, the AEs resolved completely within 3 days after injection[9]. Systemic AEs were reported by 67% of the participants, of which most AEs (> 83%) were classified as unrelated or unlikely related to the treatment administered. Possibly related AEs (N=22, 17% of all reported AEs) were reported by 15 subjects, of which headache (N=6) and rhinitis (N=3) were the most frequently reported possibly related AEs. Control virosomes resulted in not related systemic AEs (see section 5.4.3.1.2 for definition). In addition, no clinically significant changes in hematological and biochemical abnormalities were observed.

The nature and characteristics of the symptoms described for influenza virosomes-based vaccines are generally in line or even better when compared with observations made for inactivated virus protein-based vaccines that are currently on the market.

3.4.1 Pre-clinical Studies with Gp41 Antigens

As virosome is a very flexible technology, it could be easily adapted to HIV-1 vaccine. Mymetics has conducted its first immunogenicity and tolerance study on non-human primates (monkey species *Macaca mulatta*) in 2005-200610. This study was conducted by the Institute of Laboratory Animal Science & Chinese Academy of Medical Science (ILAS) in Beijing. ILAS, a globally recognized analytical facility, have and continue to be used by leading US scientists and major governmental entities. The aim of that study was to determine if female macaques could produce mucosal antibodies into their genital compartment upon vaccination with pre-clinical batch similar to MYM-V101 (pcMYM-V101). A total of

16 female macaques having between 5-8 years old were vaccinated. Animals were subdivided into 3 groups for investigating the optimal immunization route; all animals have received 4 administrations of 40 µg of pcMYM-V101 over a 6 months period (Weeks 0, 4, 12 and 24). Group 1 (N=5) was vaccinated by i.m. injections, Group 2 (N=5) received the vaccine by i.n. administrations, while Group 3 (N=6) started with 2 i.n. administrations (Weeks 0 and 4) followed by 2 i.m. administrations (Weeks 12 and 24).

More than 90% of the vaccinated animals had specific IgG as well as IgA antibodies against P1 peptide in their vaginal secretions. Functionality was tested in an *in vitro* transcytosis assay, a technique that evaluates the capacity of mucosal antibodies to prevent HIV-1 transfer across a mucosal epithelium. It was shown that antibodies contained in the vaginal secretions were efficiently blocking HIV-1 transcytosis (50-90% inhibition) from clade B and C viruses. Considering that secretions were diluted about 20-fold in the assays, *in vivo* these secretions would be more concentrated and likely more potent. Due to limited material and low amount of antibodies in cervico-lavages, total IgA antibodies from few animals could be purified and tested in neutralization assays, a technique that evaluates the potential of antibodies to block cell infection by primary HIV-1 strains. Neutralisation assays showed that at least one animal had IgA with significant neutralizing activity against a primary HIV-1 clade B *in vitro*. Results from rectal lavages showed that all macaques had specific IgA antibodies against the P1 peptide, although specific IgG could almost not be detected, which is normal for this type of secretions, as mentioned before. The antibody content of rectal lavages could also block HIV-1 transcytosis *in vitro*. In overall, mucosal antibodies induced in monkeys were very potent to inhibit HIV-1 entry *in vitro* (transcytosis blocked), while neutralizing activity was more difficult to evaluate due to low mucosal antibody concentration, as it has been observed with human mucosal secretion from HIV-1 resistant subjects. These pre-clinical results represent the “animal **proof of concept”** that mucosal antibodies can be induced by pcMYM-V101 vaccination.

Most sera from immunized animals contained high levels of specific anti-P1 IgG antibodies, but they had no significant inhibitory capacity when tested in transcytosis and neutralizing *in vitro* assays. These results suggest that blood IgG and mucosal IgA have different inhibitory potential, despite recognizing the same antigen. In this animal study, immunizations with pcMYM-V101 have induced low amounts of mucosal antibodies exhibiting strong inhibitory potential for preventing *in vitro* HIV-1 transcytosis (entry across mucosal tissue), while neutralizing activity is difficult to measure, based on secretion samples. Furthermore, we observed that the antibody content in secretions was varying from sample lavage to sample lavage, underlying the importance of multiple/sequential samplings in this kind of study. It is difficult to know if low levels of specific mucosal antibodies would be protective, as no animal challenges were performed on this exploratory study. Meanwhile, it is believed that what really matter is to have the B lymphocytes anti-P1 locally present in the genital compartment, ready to produce huge amount of mucosal antibodies when locally exposed to the antigen.

A second study on non-human primate *Macaca mulatta* was launched in Fall 2007 for evaluating the *in vivo* protection level induced by vaccination with an improved vaccine formulation, based on gp41-derived antigens11. This study differs from the previous one, regarding the vaccine composition. In addition to the optimized virosomes-P1 (MYM-V101), a second type of virosome containing the recombinant gp41 (rgp41) protein produced in bacteria *E. coli* was formulated. Therefore, the tested vaccine was made of two different virosomes: pcMYM-V101 and virosomes-rgp41, both mixed in equal protein quantity. It was postulated that the P1 peptides would favour the induction of protective mucosal antibodies, as observed in Nature, while the rgp41 would favour protective blood antibodies. The antibody repertoire induced by P1 and rgp41 might lead to optimal protection.

A total of 18 female macaques, subdivided in 3 groups of 6 animals, received four injections over a six month period (October 2007 to April 2008): Week 0, 7, 15 and 24. Each vaccine dose was containing 40

µg of P1 peptides and 40 µg of rgp41 (combined together). Group 1 corresponds to the placebo group that received i.m. injections of virosomes alone (IRIV, no HIV-1 antigens). Group 2 has received i.m. the vaccine virosomes-P1 combined with virosomes-rgp41, while group 3 has received the same formulation but two i.m. injections at Weeks 0 and 7, followed by two i.n. administrations at Weeks 15 and 23. Unfortunately, one animal from group 3 died at the beginning of the study. After autopsy, the animal death was unrelated to the vaccine and the death cause could not be identified. It is believed that i.m. injection might target immune compartments that overlap or differ from those targeted by the i.n. administration: systemic versus mucosal compartment. Combining both administration routes may lead to a broader coverage of the various immune compartments, i.e. saliva (mouth), upper aero-digestive tract (lungs), genital and rectal compartments, as opposed to a single injection route that may lead to a more restrictive coverage.

More than 50% of the vaccinated animals had specific IgA antibodies against P1 peptide in their vaginal secretions, using ELISA tests. Unfortunately in this study, mucosal antibodies in rectal and some vaginal secretions could not be measured due to too high background levels of pre-immune samples. The functionality of these mucosal IgA antibodies will be investigated; i.e. their capacity to: 1) prevent HIV-1 transfer across a mucosal epithelium (*in vitro* transcytosis assay); and to 2) block cell infection by primary HIV-1 strains (*in vitro* neutralization assay). Considering the low amount of IgA antibodies in vaginal lavages, total IgA antibodies will be purified and if the material is in sufficient quantity, than functional assays could be performed. Supplementary analysis will also provide important information (qualitative and quantitative) regarding specific anti-P1 antibody responses present in the serum of these animals.

One month after vaccination, meaning early May 2008, all animals were intra-vaginally challenged thirteen times with SHIVSF162P3 received from the NIAID (National Institute of Allergy and Infectious Diseases), NIH (National Institutes of Health) (Bethesda, USA). This chimeric SHIV virus (Simian Human Immunodeficiency Virus) has the gp160 (gp120 + gp41), Tat, rev and vpu of HIV-1 clade B and all the remaining genes/proteins are from the SIV (Simian Immunodeficiency Virus). Intra-vaginal challenges were done every 4-7 days with 30 TCID50 for mimicking the natural HIV transmission in human. Blood samples were drawn every 4 days for measuring the plasma viral load (viremia) in monkeys, using the Qiagen QuantiTect SYBR Green RT-PCR kit with specific SIV gag probes (sensitivity 1000 copies/mL blood).

All animals from the placebo group (IRIV) were infected, the majority (4 out of 6 monkeys) becoming positive between Day 24 and 38 after the first challenge (Day 0). The two remaining animals had a detectable viremia starting at Day 51 and 86. These results are not unusual; the HIV-1 infection rate for human (man to women) is about 1 chance out of 200 for each sexual intercourse, so several exposures to the virus is required before having 100% individual infection. The most important aspect of this study is that Group 3 remains with undetectable SHIV viremia over five months. Only 1 animal out of 5 became positive and the virus detection in this infected animal was possible only for Day 114 post first challenge, before or after Day 114 the animal has no detectable viruses, suggesting a transient viremia. Furthermore, the viral load of this animal is at least one log inferior to the viral loads retrieved in the placebo control group. All the remaining 4 animals had no detectable viremia during five months of follow-up.

As previously reported for SHIV and HIV-1 infection, there is no correlate of protection yet identified. Our monkey results have generated similar observations, as animals having undetectable, low or intermediate mucosal antibody levels were similarly protected during repeated intra-vaginal low-dose challenges with SHIVSF162P3. This suggests that antibodies were likely produced locally at the time of the challenge but their detection by ELISA is limited by the detection threshold. Furthermore, it can't be

exclude the possibility that these animals from Group 3 are infected with low virus load that is undetectable due to detection limit. Meanwhile, there is at least a 2-log difference between the control and Group 3, which might have a significantly impact on the HIV-1 transmission rate and disease evolution, if such results had to be observed on humans.

Meanwhile, it is difficult to establish if the protection was more related to pcMYM-V101 or to virosomes-rgp41. For improving our knowledge on the defense mechanisms behind this protection , over the coming years other vaccination experiments on non-human primates will be conducted for testing each antigen alone or in combination, measuring the innate responses as well as mucosal and blood antibodies. Specific anti-P1 and anti-rgp41 antibodies will be purified, characterized and depleted before performing neutralization and transcytosis assays.

These breakthrough monkey results represent the **"proof of concept"** that virosomes-based vaccine containing HIV-1 gp41-derived antigens might offer a significant protection against HIV-1 or control the virus replication. Such results are extremely encouraging for the continuation of the vaccine development.

3.4.2 Toxicology Studies with MYM-V101

Having shown that pcMYM-V101 vaccine could successfully induce mucosal antibodies in an animal species closed to humans, Mymetics has further continued the vaccine development. From April to October 2008, a set of studies has been performed under GLP (Good Laboratory Practice) compliance for investigating dose toxicity of the pre-clinical MYM-V101 formulations in order to test its safety. Four different formulations with the P1 antigens were done:

For rat studies:

- **MYM-V101.a** corresponding to low dose group at 10 μg dose/100 μL for i.m. injection;
- **MYM-V101.b** corresponding to 30 μg dose/40 μL for i.n. administration;
- **MYM-V101.c** corresponding to high dose group at 50 μg dose/100 μL for i.m. injection;
- **MYM-IRIVm** for the i.m. placebo group;
- **MYM-IRIVn** for the i.n. placebo group;

For rabbit studies:

- **MYM-V101.d** corresponding to high dose group at 50 μg dose/500 μL for i.m. injection;
- **MYM-IRIVm** for the i.m. placebo group.

In the first study, the vaccine was tested in an 8-week repeated dose study with 3 different treatment Groups[12]. Group 1 (N=40) was the placebo control Group receiving MYM-IRIV. Animals from Group 2 (N=20) received the low dose vaccine (MYM-V101.a: 10 μg/injection), while animals from Group 3 (N=40) received the high dose vaccine (MYM-V101.c: 50 μg/injection). Animals of Group 1 and 3 were separated in a subgroup of 20 animals for performing the main study; these animals were sacrificed 2 days after the last vaccination. The second subgroup of 20 animals from Groups 1 and 3 were part of the recovery study; these animals were sacrificed 4 weeks after the last vaccination.

Each Group consisted of male and female rats in an equal distribution. All animals were vaccinated every other weeks for 8 weeks, so in total on 5 occasions. Vaccinations were performed by i.m. injections into the right hind leg. During performance of the study, the following assessments were carried out: any sign of illness or reaction to treatments, neurological examinations, mortality, body weight and food consumption. Prior to dissection, 2 days after the last vaccination of the main study Groups, blood was taken for biochemistry, haematology and antibody determinations. Urine was collected for urinanalysis. Vaginal and rectal washes were performed prior to and after the vaccination for assessment of mucosal

antibodies. I.n. washes were performed only after sacrificing animals. Two days after the last vaccination, the main study animals were dissected (Day 58). Necropsy of all animals allocated to the recovery period was performed 4 weeks after the last vaccination. Macroscopic examination was performed by observing the appearance of the tissues *in situ*. Subsequently, part or totality of the organs was collected for pathology and histopathology of the tissues.

In a second study, a repeated dose toxicity study covering a ten-week period, the vaccine was administered three consecutive times to male and female Wistar rats via **i.m.** injection on Days 0, 14 and 28, followed by three consecutive **i.n.** administrations on Days 42 and 56 and 70[13]. Animals were dosed with MYM-V101.c (50 µg) via the i.m. route and with MYM-V101.b (30 µg) via the i.n. route, while animals in the control group received IRIV virosomes. Recovery animals were included in both study groups, which were observed for another 4 weeks after the last treatment. All observations as described for the 8-week toxicity study were also performed for this study.

In summary, no toxicological findings were observed in either of these rat repeated dose toxicity studies. The local effects at the injection sites were of minimal severity and seen in a low number of treated animals only. Repeated i.n. administration of the test item was well tolerated in the nasal cavity and nasopharynx.

In both studies, some effects in the popliteal lymph node showed that an immunological response had occurred, as was demonstrated by the presence of anti-P1 immune responses. This is considered to be related to the immunogenic potency of hemagglutinin.

A third study was conducted in parallel for investigating the local tolerance to repeated doses of the vaccine. Administration were done 8 times into the hind leg of female New Zealand White rabbits via **i.m.** injection in the right hind leg on Days 0, 7, 14, 21, 28, 35, 42 and 49[14]. Five animals were dosed with MYM-V101.d (50 µg/500µL), while animals in the control group (N=5) received MYM-IRIV virosomes. Mortality, clinical signs and body weight were observed throughout the study. The injection site was carefully examined for the occurrence of erythema or oedema at 1, 24 and 48 hours after each administration. At necropsy (2 days after the last administration), a macroscopic examination was performed and histopathological examinations were on a reduced set of organs and organ systems.

In summary, no clinical findings related to the administrations were observed in any animals and no erythema or oedema were observed, neither in the control nor in the MYM-V101-treated groups. Some effects in the popliteal lymph node showed that an immunological response had occurred, which was again considered to be likely related to the immunogenic potency of hemagglutinin present at the surface of virosomes.

In conclusion, non-clinical data clearly demonstrated that pre-clinical MYM-V101 shows an acceptable safety/efficacy ratio in animals.

Having conducted successfully all the pre-clinical steps on the MYM-V101, Mymetics Corporation feels confident that this virosomes-based product will be safe and well tolerated in humans. Furthermore, three other virosomes-based vaccines against malaria, breast cancer and hepatitis C are under investigation or have already been evaluated in human Phase I trials, and so far all these virosomes-based vaccine candidates have been well tolerated. Vaccine strategies for developing mucosal protection against HIV-1 remain to be investigated. Despite that the MYM-V101 vaccine could successfully trigger mucosal antibodies in animals, its immunogenicity in human mucosal compartments is unknown.

Meanwhile, the study by Glück *et al.*[15] has compared the immunogenicity of the influenza-virosome administered i.m. or i.n., and it appeared that i.n. vaccination resulted in a much better mucosal immune response, as compared to i.m. injection. This study has evaluated only mucosal IgA in the saliva,

and the best mucosal response was obtained with Heat labile toxins (HLT)-adjuvanted virosomes. Systemic immune responses, measured as influenza virus-specific IgG antibodies in blood, were comparable between i.n. and i.m. administrations. Other studies have looked for IgA in lung lavages or in serum but to our best knowledge, mucosal antibodies in the genital and rectal compartments never have been evaluated upon vaccination with influenza virosomes-based vaccines. All nasal and i.m. vaccinations were well tolerated in the various clinical trials. It is hypothesized that the local stimulation and priming of the nasal mucosal associated immune system might result in a generalized stimulation of the entire mucosal immune system, as well as the systemic immune system.

The present study was designed to investigate the safety and tolerability of the newly developed MYM-V101 vaccine in healthy female subjects. I.m. administrations will be combined with i.n. administrations to gain a better mucosal immune response in vagina and rectum, the main entrance doors of HIV-1. As opposed to previous studies investigating either the i.n. or the i.m. administration routes, this proposed study will test both routes with only virosomes as adjuvant (no HLT or others). Furthermore, this study is the first developed to investigate both the local and systemic immune responses in vaginal and rectal secretions, and in blood. Furthermore, functionality of the specific antibodies will also be assessed.

4 Objectives

4.1 Primary Objective

The primary objective is to demonstrate the safety and tolerability of MYM-V101 vaccine after two i.m. injections in combination with two i.n. administrations.

4.2 Secondary Objective

The secondary objective is to evaluate the blood humoral immune response against the MYM-V101 vaccine.

4.3 Ancillary Objectives

The ancillary study has been designed for investigating the mucosal immune responses. Depending on the biological material availability and quality, additional assays might be conducted for quantifying mucosal antibodies and their functional activities. Study of the B cell phenotype evolution during vaccination with MYM-V101 will also be investigated.

5 Methods

Details on the timing of the treatments and assessments are given in the flowchart (see section 2).

5.1 Trial Design

5.1.1 Overview of trial

This is a first-in-man, dose escalating Phase I study, single center, randomized, double-blind, placebo-controlled to examine the safety of and tolerability to a virosomes-based vaccine called MYM-V101 containing P1 peptides derived from the gp41 protein of HIV-1, administered i.m. in combination with i.n. administrations. The proposed virosomes-based vaccine has been designed for elicitating the

antibody response against the HIV-1 gp41 protein for potentially preventing HIV-1 transmission/infection.

The study population will consist of 24 healthy adult female subjects, equally distributed over 2 Panels (Panels 1 and 2). In a blinded mode for each Panel, 8 subjects will receive the active vaccine MYM-V101 and 4 subjects will receive the placebo (MYM-IRIV). Randomization will be done per Panel. Treatment in the Panels will be administered in a dose escalation design as follows:

Panel 1. Subjects will receive 2 i.m. injections of MYM-V101.1 (10 µg/0.5 mL), administered at Weeks 0 (Visit 1) and 8 (Visit 4), followed by 2 i.n. administrations of MYM-V101.4 (10 µg/0.2 mL), administered at Weeks 16 (Visit 6) and 24 (Visit 8). The subjects will receive the second i.m. administration (Visit 4) once all subjects have completed Visit 2, and only if the first i.m. vaccination was found safe and tolerable after a safety review on the data of the first week after vaccination. The second i.n. administration (Visit 8) will only be given when all subjects have completed Visit 7, and after a safety review on the data of the first week after i.n. administration. No more than 5 subjects will start vaccination at the same day during Visit 1. Subject monitoring will end 5 weeks after the last vaccination.

Panel 2. Subjects will receive 2 i.m. injections of MYM-V101.2 (50 µg/0.5 mL), administered at Weeks 0 (Visit 1) and 8 (Visit 4), followed by 2 i.n. administrations of MYM-V101.3 (50 µg/0.2 mL), administered at Weeks 16 (Visit 6) and 24 (Visit 8). Panel 2 will start treatment after all subjects in Panel 1 have completed Visit 2, and only if the 10 µg i.m. dose was found to be safe and tolerable. Subjects in Panel 2 will receive the second i.m. administration (Visit 4) once all subjects in Panel 2 have completed Visit 2, and only if the first high dose i.m. vaccination was found safe and tolerable after a safety review on the data of the first week after i.m. vaccination. Subjects in Panel 2 will receive the first i.n. administration (Visit 6) once all subjects in Panel 1 have completed Visit 7 (10 µg i.n.) and all subjects in Panel 2 have completed Visit 2 (50 µg i.m.), and only if the aforementioned vaccinations were found to be safe and tolerable. Subjects in Panel 2 will receive the second i.n. administration (Visit 8) once all subjects in Panel 2 have completed Visit 7, and only if the first high dose i.n. vaccination was found safe and tolerable after a safety review on the data of the first week after i.n. vaccination. No more than 5 subjects will start vaccination at the same day during Visit 1. Subject monitoring will end 5 weeks after the last vaccination.

Furthermore, the stability of the IMPs will be monitored in real time and cumulative P1 antigen associated degraded mass will be calculated throughout the study. If necessary, dose adjustments may follow.

Occurrence of local and systemic AEs will be assessed until 2 hours after each vaccination. Subjects will be asked to daily record solicited AEs on diary cards from Day 1 to Day 7 after each vaccination, which will be reviewed by the study personnel during the next visit to the clinical unit. Other diary cards will be used from Week 2 to Week 8 after each vaccination to record AEs, if applicable. Occurrence of clinically significant hematological and biochemical abnormalities will be followed throughout the trial. Safety and tolerability evaluations will be continuously recorded.

For the secondary objective of the study, serums will be taken to assess for the presence of blood anti-P1 antibodies (anti-HIV-1 gp41) at several time points throughout the trial. Ancillary study will also be conducted with vaginal and rectal secretions to assess for the presence of mucosal IgA and IgG anti-P1 antibodies. Peripheral blood lymphocyte samples are also required for studying the mucosal B cell phenotype evolution during vaccination. Mucosal and blood antibodies induced by the MYM-V101 vaccine might be assessed for their capacity to block HIV-1 infection and transcytosis *in vitro*.

A schematic overview of the trial is provided in Figure S1.

Panel 1
2 i.m. injections of 10 µg MYM-V101 at
Weeks 0 and 8 followed by
2 i.n. administrations of 10 µg MYM-V101 at
Weeks 16 and 24 (N=8)

OR

2 i.m. injections of MYM-IRIVm at
Weeks 0 and 8 followed by
2 i.n. administrations of MYM-IRIVn at Weeks 16
and 24 (N=4)

Interim Safety Review
(after completion of Visit 2 and Visit 7 of Panel 1)

Panel 2
2 i.m. injections of 50 µg MYM-V101 at
Weeks 0 and 8 followed by
2 i.n. administrations of 50 µg MYM-V101 at
Weeks 16 and 24 (N=8)

OR

2 i.m. injections of MYM-IRIVm at
Weeks 0 and 8 followed by
2 i.n. administrations of MYM-IRIVn at Weeks 16
and 24 (N=4)

Interim Safety Review
(after completion of Visit 2 and Visit 7 of Panel 2)

Figure 1S: Schematic Overview of the Trial.

5.1.2 Discussion of Trial Design and Selection of Dose(s) in the Trial

Today, there is no efficient prophylactic vaccine against HIV-1. All past vaccines did not protect and current vaccines under investigations are inducing immune responses likely unable to prevent infection. Meanwhile, these vaccines were designed for triggering immune responses that might likely control the virus replication, potentially leading to subjects with a low viremia and consequently, poorly infectious for others. Until now, this type of vaccines has not provided yet such results. Furthermore, none of the previously HIV-1 designed vaccines could specifically induce an efficient mucosal antibody response at the main entrance doors of HIV-1; the genital and rectal compartments.

Mymetics Corporation understood many years ago the importance of triggering mucosal protection as a potential efficient first line of defense against HIV-1 transmission/infection. Immunogenicity results on animals are very convincing and suggest that virosomes could target the mucosal compartments. Virosomes, which are round-shaped capsules mostly made of phospholipids and influenza derived proteins HA and NA, represent an innovative antigen delivery system, which has already proven its suitability to elicit protective immune responses against vaccine components in humans. The aim of this trial is to prove the principle that virosomes are a suitable delivery system for P1 peptides derived from HIV-1 gp41 protein, which require lipid-membrane anchorage for optimal epitope presentation.

The doses and dosing regimens of MYM-V101 selected will be administered based on pre-clinical data and most importantly, based on previous experiences with virosomes developed for malaria antigens CSP (PEV302) and AMA-1 (PEV301) tested in Phase I (2003-2005) and in Phase II (2005-2007) on healthy adult subjects[10]. Furthermore, for biological compounds no clear dose response can be found when the administered dose is doubled, so the five-fold increase in dose is based on previous clinical trials with virosomes-based vaccines in doses of 10, 40 and 50 µg of antigen. The safety data of these studies showed that the dose increases were safe and well tolerated. Toxicology data with MYM-V101 in doses of 10, 30 and 50 µg also showed that the doses were safe and well-tolerated in rats and rabbits. I.m. injection should be performed in the anterolateral site of the leg, close to the inguinal lymph nodes that may drain antigens from the genital areas. Due to this proximity, it should favor higher local antibody production for optimized targeting of the vaginal and rectal mucosa. The day of vaccination must match the woman's cycle corresponding to Day 1 to Day 5 after ovulation, which is about the mid-cycle time point. The time window for mucosal samplings (Day 21-26 of the woman's cycle) likely corresponds to a cycle period with detectable mucosal antibodies.

As mentioned before, i.n. administrations may likely target different immune compartments with a different efficacy, respective to i.m. injections. It is hypothesized that vaccinations that combine i.m. injections followed by i.n. administrations may induce a broader immune response, such as both systemic and mucosal antibodies. Therefore, the i.n. administrations are investigated to explore the potential synergy between the two routes of administration, since it has been described that i.n. vaccination results in a better mucosal immune response.

To explore the safety and tolerability of MYM-V101, subjects will be monitored for local and systemic adverse reactions up to 2 hours after each vaccination. Subjects must daily complete diary cards to record adverse reactions over the whole treatment period. Diary cards will be evaluated by the study personnel at each visit to the clinical unit. Eight weeks between each vaccination is necessary to guarantee an effective immune response. In Panels 1 and 2, an interim safety review will take place 7 days after all subjects have completed Visit 2 and Visit 7, and the safety data of the first week after first i.m. or i.n. immunization have been reviewed.

Subjects in Panel 1 will receive the second i.m. administration (Visit 4) once all subjects have completed Visit 2, and only if the first i.m. vaccination was found safe and tolerable. The second i.n. administration (Visit 8) will only be given when all subjects have completed Visit 7, and after a safety review on the data of the first week after i.n. administration.

Vaccination in Panel 2 will start after all subjects in Panel 1 have completed Visit 2, and only if the 10 µg i.m. dose was found to be safe and tolerable. Subjects in Panel 2 will receive the second i.m. administration (Visit 4) once all subjects in Panel 2 have completed Visit 2, and only if the first high dose i.m. vaccination was found safe and tolerable. Subjects in Panel 2 will receive the first i.n. administration (Visit 6) once all subjects in Panel 1 have completed Visit 7 (10 µg i.n.) and all subjects in Panel 2 have completed Visit 2 (50 µg i.m.), and only if the aforementioned vaccinations were found to be safe and tolerable. Subjects in Panel 2 will receive the second i.n. administration (Visit 8) once all subjects in Panel 2 have completed Visit 7, and only if the first high dose i.n. vaccination was found to be safe and tolerable.

Furthermore, after the stability issues found with the first batches of the IMPs, the IMPs will now be produced in two small batches that will be used within approximate 6 months after production. By using the IMPs relatively rapidly after manufacturing, the amount of P1 antigen degradation products administered to subjects can be limited and kept below a safety threshold of 30 µg of cumulative P1 antigen associated degraded mass as calculated from the pre-clinical studies. The stability of the IMPs will be monitored in real time and cumulative P1 antigen associated degraded mass will be calculated throughout the study. If necessary, dose adjustments may follow.

Local immune responses are measured in vaginal and rectal secretions. A sensitive immuno-assay has been developed for the detection of specific IgG and IgA antibodies against the P1 peptide derived from the HIV-1 gp41 protein. Setting up this sensitive technology was required due to the low mucosal antibody quantity available. For comparison, serum may easily contain > 1mg/mL of antibodies, while mucosal secretions have about 1-30 µg of total antibodies per sampling, which is about 100-fold lower. This new technique has a sensitivity of about 100 to 1000-fold higher than standard ELISA tests, with a detection limit of about 0.1 ng/mL. This offers the advantage of using very limited amount of working material (like 1-5 µl of secretion), leaving more material for conducting other functional assays (transcytosis and HIV-1 neutralization assays).

5.2 Trial Population

Screening for eligible subjects will be performed within 3-5 weeks prior to the first vaccination.

5.2.1 Sample Size

A total of 24 non-institutionalized healthy female subjects will be selected.

Sample size rationale is shown in section 5.5.1: Determination of Sample Size.

If randomized subjects discontinue the trial before receiving their first vaccination, additional subjects may be recruited to have 24 subjects starting vaccination. No additional subjects will be recruited if a subject is prematurely withdrawn from the trial after starting vaccination.

5.2.2 Inclusion Criteria

Subjects must meet **all** of the following inclusion criteria:

1. Female, aged between 18 and 45 years, extremes included;
2. Having regular menstrual cycles (24 to 30 days). Women that got sterilized by bilateral tubal ligation are allowed, as long as they have regular cycles;
3. Non-smoking or smoking (no more than 10 cigarettes or 2 cigars or 2 pipes per day, for at least 3 months prior to selection);
4. Body Mass Index (BMI) of 18.0 to 30.0 kg/m^2, extremes included. BMI is calculated as the weight (in kg) divided by the square of height (in m);
5. Informed Consent Form (ICF) signed voluntarily before first trial-related activity;
6. Able to comply with all protocol requirements;
7. Healthy on the basis of a medical evaluation that reveals the absence of any clinically relevant abnormality and includes a physical examination, a gynecological examination, medical history, electrocardiogram, vital signs and the results of blood biochemistry and hematology tests and a urinalysis carried out at screening;

8. If the subject is of childbearing potential, agrees to use adequate contraception (oral contraceptives or double barrier method, as described in section 5.2.4, point 7) and not become pregnant for the duration of the study.

5.2.3 Exclusion Criteria

Subjects must **not** have any of the following characteristics:

1. Male;
2. Female subject without regular menstrual cycle (24-30 days);
3. History of total hysterectomy;
4. Female subject of childbearing potential without use of effective birth control method(s), or not willing to continue practicing these birth control method(s) for the duration of the trial;
5. Spermicides or other chemicals as used in contraceptive barrier methods (i.e., a male or female condom, diaphragm, cervical cap or intra-uterine device);
6. A positive pregnancy test or breast feeding at screening;
7. A positive HIV-1 or HIV-2 test at trial screening;
8. Having vaginitis as observed by local inspection and vaginal swab;
9. Recurrent vaginal infections or sexually transmitted diseases within one year prior to vaccination;
10. A positive test for Hepatitis A (confirmed by hepatitis A antibody IgM), hepatitis B (confirmed by hepatitis B surface antigen), or hepatitis C (confirmed by hepatitis C virus antibody) infection at trial screening;
11. History or evidence of current use of alcohol, barbiturate, amphetamine, recreational or narcotic drugs-which in the investigator’s opinion would compromise subject safety or compliance with trial procedures;
12. Currently active or underlying diabetes, gastrointestinal, cardiovascular, neurological, psychiatric, metabolic, renal, hepatic, respiratory, inflammatory, auto-immune disease(s), inheritary immune deficiencie(s), infectious disease(s), nasal disorders (i.e. rhinitis, chronic nose bleeds, chronic sinusitis, polyps, chronic cold sores), dental or mouth infections, or rectal problems (fistals, hemorrhoids);
13. History of allergic disease (i.e. egg, milk, dairy products) or reaction likely to be exacerbated by any component of the vaccine to be administered in this trial, and severe allergic disease(s);
14. Contraindication to i.m. injections, history of bleeding disorder or use of anticoagulant therapy within 4 weeks prior to the first study vaccination;
15. Having received any of the following substances:
    - Nasal or inhalation corticosteroids within 2 weeks prior to first vaccination;
    - Vaginal and/or rectal treatment within 3 days prior to first mucosal sampling;
    - HIV vaccine in a prior clinical trial;
    - Immunosuppressive medications within 6 months prior to first vaccination;
    - Blood products within 120 days prior to the first vaccination;
    - Immunoglobulin within 60 days prior to the first vaccination;
    - Any investigational or non-registered drug or vaccine within 30 days preceding the first vaccination;
    - Any planned vaccination during the study period and safety follow-up;

- Allergy treatment with antigen injections, within 14 days prior to the first study vaccination;
- Current anti-tuberculosis preventive therapy or treatment;

16. Serious adverse reactions to vaccines such as, but not limited to, anaphylaxis, urticaria (hives), respiratory difficulty, angioedema, or abdominal pain or a history of anaphylactic reactions;
17. Donation of blood or plasma within the 30 days preceding the first vaccination;
18. Acute disease at the time of enrolment.

5.2.4 Prohibitions and Restrictions

1. Subjects should avoid intercourse from 3 days prior to each mucosal sampling onwards until the samples are taken;
2. Subjects should not use vaginal wash, vaginal creams, lubricants or rectal treatment from 3 days prior to each mucosal sampling onwards until the samples are taken;
3. If a subject has had an acute disease before each study vaccination, the administration of vaccine should be postponed until the body temperature is normal (≤ 37.5°C) for at least 24 hours. The vaccination may be postponed once by 1 month, synchronized with the woman's cycle. If acute diseases occur more than once, the vaccination schedule will be followed as depicted in the flowchart so vaccination will only be postponed once;
4. For the duration of the trial, smoking subjects should not smoke more than 10 cigarettes, 2 cigars, or 2 pipes per day;
5. Subjects will be advised not to donate blood for at least 1 year after the last administration of the study vaccine (1 year after the 4th vaccination);
6. Women of childbearing potential not under hormonal contraception should use a double-barrier method with a combination of two of the following contraceptive methods. In all cases the use of spermicides or other chemicals is not allowed:
   - male or female condom (male and female condom should not be used together due to risk of breakage or damage caused by latex friction);
   - diaphragm;
   - cervical cap;
   - intrauterine device (hormone free).

5.2.5 Removal of Subjects from Therapy or Assessment

Subjects should normally be withdrawn from the trial if a serious adverse event (SAE) occurs. However, the Principal Investigator can decide that the SAE does not influence the study objectives and that the subject does not necessarily need to be withdrawn from the study. These exceptions must be discussed with the sponsor.

Subjects **must** be withdrawn from the trial if:

1. They withdraw their consent;
2. The Principal Investigator considers it is in the best interest of the subject;
3. They experience diseases or AEs requiring treatment that occur after inclusion, which do not constitute SAEs but which, in the opinion of the Principal Investigator, would probably prevent achievement of the study objectives;
4. They do not comply with all protocol requirements;

5. They have a positive pregnancy test or are breast feeding after inclusion;
6. They have a positive HIV test after inclusion;
7. They receive any vaccine other than study vaccination during the trial;
8. If the treatment code is broken by the clinical unit.

The date and the reason for discontinuation must be noted on the Case Report Form (CRF) (if applicable).

All subjects prematurely discontinuing the trial must be seen, if consent is not withdrawn, for a visit and a final evaluation (see Flowchart) and the Trial Termination section of the CRF will be completed.

Screening failures including causes will be listed in the screening list. All data obtained within the screening will be documented in the volunteer's record. In case that a CRF is available it will be archived; data will not become part of the database and will consequently not be presented in the integrated clinical study report.

All details, causes and justifications for removal of included subjects from the study will be recorded in the study termination section of the CRF. All CRFs generated within the study have to be kept in the documentation of the study. Reasons for withdrawal of included subjects will be described in the integrated clinical study report.

5.2.6 Data Review Meetings

Subjects, investigators and sponsor will be blinded during the conduct of the Phase I trial. The treatment of each subject in each panel will be disclosed to the sponsor and the investigators after the completion of the trial. Subsequently, the investigators will inform the subject about the treatment they have received in the trial.

Importantly, the safety and tolerability data of the first 7 days after the first i.m. and the first i.n. vaccination of all subjects will be evaluated in a blinded way by the investigators and the sponsor prior to administer the next dose and/or escalating the dose level and/or prior to a change in the route of administration. This will be applicable for the dose increase from 10 µg (Panel 1) to 50 µg (Panel 2) per vaccination and also for change from i.m. to the i.n. administration (both Panels).If the overall safety and tolerability profile is considered safe, then the next vaccination, increased dose, or route of administration may be given as planned. If the safety and tolerability data are not acceptable, the sponsor in conjunction with the Principal Investigator can decide to suspend further dosing or adjust the dose planned for the next session based on the data of the review. See also section 5.2.7 for rules on dose escalation.

Subjects in Panel 1 will receive the second i.m. administration (Visit 4) once all subjects have completed Visit 2, and only if the first i.m. vaccination was found safe and tolerable. The second i.n. administration (Visit 8) will only be given when all subjects have completed Visit 7, and after a safety review on the data of the first week after i.n. administration.

Vaccination in Panel 2 will start after all subjects in Panel 1 have completed Visit 2, and only if the 10 µg i.m. dose was found to be safe and tolerable. Subjects in Panel 2 will receive the second i.m. administration (Visit 4) once all subjects in Panel 2 have completed Visit 2, and only if the first high dose i.m. vaccination was found safe and tolerable. Subjects in Panel 2 will receive the first i.n. administration (Visit 6) once all subjects in Panel 1 have completed Visit 7 (10 µg i.n.) and all subjects in Panel 2 have completed Visit 2 (50 µg i.m.), and only if the aforementioned vaccinations were found to be safe and

tolerable. Subjects in Panel 2 will receive the second i.n. administration (Visit 8) once all subjects in Panel 2 have completed Visit 7, and only if the first high dose i.n. vaccination was found to be safe and tolerable.

5.2.7 Definition of Dose Limiting Toxicity and Maximum Tolerated Dose

Dose Limiting Toxicity (DLT)

Each dose escalation or change in route of administration shall be performed if, and only if, in the judgment of the investigator and the experts of the sponsor, the results of the safety analyses of the preceding dose or preceding route of administration are satisfactory.
Within 7 days after all subjects have completed Visit 2 and Visit 7 in Panels 1 and 2, local and systemic AEs, vital signs and laboratory tests of the first 7 days after vaccination will be reviewed blinded and evaluated, and a decision to increase the dose or to continue with the next i.m. or i.n. vaccination will be made.

Consideration that will prevent further dose escalation or change in route of administration will come out if one of the following observations occurs:

1. If at least 1 subject in a panel has an AE that has been assessed as causally related by the investigator and that may seriously jeopardize her health or the severity prevents further evaluation, as judged by the investigator and sponsor;

2. If ≥ 2 subjects in a panel have a moderate AE on laboratory tests (confirmed values) that has been assessed as causally related by the investigator;
3. If ≥ 3 subjects in a panel have moderate toxicity grading changes in blood pressure that have been assessed as causally related by the investigator.

To establish if any of the above specified three criteria have been met, a blinded review of safety information is planned within seven days after all subjects have completed Visit 2 and Visit 7 of Panels 1 and 2. If input on the subject's active treatment status is necessary, an external party will confirm this status in a blinded way. The investigator and the trial team will remain blinded during this evaluation. The confidentiality of any unblinded information will be maintained until trial termination.

Furthermore, as described in section 0, the IMPs will now be produced in two small batches that will be used within approximate 6 months after production, thereby limiting the amount of P1 antigen degradation products administered to subjects. The safety threshold is defined as the cumulative P1 antigen associated degraded mass as found in the pre-clinical studies in rats. For this study, the safety threshold is set at 30 µg in total. So the total amount of degraded P1 mass in the IMPs to be administered to the subjects should not exceed this safety threshold of 30 µg.

Dose Escalation Rules and Maximum Tolerated Dose (MTD)

The following guide for dose escalation will be applied if the sponsor or the sponsor in conjunction with the Principal Investigator agrees to further continue the dosing:

- If, in a given panel, ≤ 1 DLT is observed, then the next panel may start;

- If ≥ 2 DLTs in a given panel are observed, then confirmation of the active treatment status of the subjects will be requested in a blinded way. MTD is considered exceeded when ≥ 2 DLTs in a given panel

while being on active treatment. The prior dose may be considered MTD. If the prior dose corresponding to 10 µg is not considered as the MTD, it might be proposed to administer an intermediate dose corresponding to about half of the volume, meaning that the subject could receive 25 µg instead of the 50 µg dose.

In order to calculate the safety threshold, the stability of the IMPs will be monitored in real time and cumulative P1 antigen associated degraded mass will be calculated throughout the study. If necessary, dose adjustments may follow based on the calculated cumulative amounts of P1 antigen degradation products.

5.2.8 Trial Termination Criteria

The trial is terminated once the trial objectives are completed and vaccination in Panel 2 has been completed with follow-up until 5 weeks after the last vaccination.

5.3 Treatment

5.3.1 Identity of Investigational Products

The investigational product, MYM-V101, will be manufactured according to GMP compliance and provided under the responsibility of Mymetics Corporation (Epalinges, Switzerland). The amount of antigens to be injected is determined by the quantity of specific gp41/P1 peptides present in the virosome formulation, among the other proteins derived from the influenza virus (HA and NA are viral proteins derived from the influenza virus H1N1, strain A/Singapore/6/86).

The MYM-V101 vaccine for the 2 different treatments is formulated as follows:

MYM-V101.1: at a concentration of 20 µg/mL, 0.5 mL injection resulting in a 10 µg dose. In addition to the 10 µg of P1 peptides, the vaccine will contain 10 µg of HA and 100 µg of phospholipids, prepared in PBS, pH 7.2-7.4. The vaccine is free of antibiotics, aluminium and thiomersal and contains very low concentration of ovalbumine. MYM-V101.1 will be used in Panel 1.

MYM-V101.2: at a concentration of 100 µg/mL, 0.5 mL injection resulting in a 50 µg dose. In addition to the 50 µg of P1 peptides, the vaccine will contain 10 µg of HA and 100 µg of phospholipids, prepared in PBS, pH 7.2-7.4. The vaccine is free of antibiotics, aluminium and thiomersal and contains very low concentration of ovalbumine. MYM-V101.2 will be used in Panel 2.

MYM-V101.3: at a concentration of 250 µg/mL, 0.1 mL per nostril resulting in a 50 µg total dose. In addition to the 50 µg of P1 peptides, the vaccine will contain 10 µg of HA and 100 µg of phospholipids, prepared in PBS, pH 7.2-7.4. The vaccine is free of antibiotics, aluminium and thiomersal and contains very low concentration of ovalbumine. MYM-V101.3 will be used in Panel 2.

MYM-V101.4: at a concentration of 50 µg/mL, 0.1 mL per nostril resulting in a 10 µg total dose. In addition to the 10 µg of P1 peptides, the vaccine will contain 10 µg of HA and 100 µg of phospholipids, prepared in PBS, pH 7.2-7.4. The vaccine is free of antibiotics, aluminium and thiomersal and contains very low concentration of ovalbumine. MYM-V101.4 will be used in Panel 1.

MYM-IRIV or Placebo: Influenza-virosome carrier alone without P1 peptides, formulated as a solution for i.m. injection (0.5 mL of MYM-IRIVm) or for i.n. administration (0.2 mL of MYM-IRIVn): 10 µg of HA and 100 µg of phospholipids per dose. The placebo solution is manufactured by Pevion Biotech AG, it will be packed, labelled and provided under the responsibility of Mymetics Corporation. Placebo formulations will both be used in Panels 1 and 2.

5.3.2 Packaging and Labeling

The investigational products MYM-V101.1, MYM-V101.2 and MYM-IRIVm for i.m. injections are provided in a 1.25 mL Syringe Hypak SCF, Becton Dickinson, Product Number: 100539 with a Hypak SCF, Stopper, Product Number: 31586.

The investigational product MYM-V101.3, MYM-V101.4, and MYM-IRIVn for i.n. administrations are provided in 0.5 mL BD Accuspray™, Becton Dickinson, Product Number: 47140719.

Each BD syringe and Accuspray™ will be packed and labeled per subject under the responsibility of Pevion Biotech AG.

Labels will contain at least:

- Clinical trial number;
- Treatment number;
- Batch or reference number;
- Expiry date;
- Storage statements;
- Dispensing instructions.

In addition, the vaccines will be labeled according to the local regulatory requirements.

Packaging of all the investigational products will allow blinded administration.

No vaccines can be repacked or relabeled without prior approval from the sponsor.

5.3.3 Randomization

Twelve subjects will be randomized to each Panel. Allocation to a Panel will be done in consecutive order; Panel 1 will be filled first followed by Panel 2.

- Subjects in Panel 1 will receive a treatment coded from 101 to 112;
- Subjects in Panel 2 will receive a treatment coded from 201 to 212;

In each panel, 8 subjects will receive active treatment with MYM-V101 and 4 subjects will receive placebo MYM-IRIVm or –IRIVn in a double-blind way.

For both Panels, 3 sets of spare medication will be provided, 2 active treatments and 1 placebo per Panel.

If spare medication is needed, the clinical site should contact M.A.R.C.O. to receive the right treatment allocation in double-blinded way.

One randomization list will be prepared, including the random numbers for both panels. The randomization list will be prepared by M.A.R.C.O.. Within each panel, subjects will receive treatment in consecutive numeric order, based on the order of arrival in the clinical unit at Visit 1, starting with the lowest number available.

5.3.4 Dosage and Administration

An overview of the planned treatment for each panel is provided in Table 1.

Table S1: Treatment Overview.

Panel / Treatment		Treatment (N=number of subjects)	Volume
**1**	Active	A single i.m. injection of MYM-V101.1 solution at Weeks 0 and 8, and a single i.n. puff of MYM-V101.4 solution per nostril at Weeks 16 and 24 (N=8).	0.5 mL solution at 20 µg/mL for i.m. injection of MYM-V101.1;<br>0.2 mL solution at 50 µg/mL (100 µl/nostril) for i.n. injection of MYM-V101.4
	Placebo	A single i.m. injection of MYM-IRIVm solution at Weeks 0 and 8, and a single puff of MYM-IRIVn solution per nostril at Weeks 16 and 24 (N=4).	0.5 mL solution for i.m. injection of MYM-IRIVm;<br><br>0.2 mL solution (100 µL/nostril) for i.n. administration of MYM-IRIVn
**2**	Active	A single i.m. injection of MYM-V101.2 solution at Weeks 0 and 8, and a single i.n. puff of MYM-V101.3 solution per nostril at Weeks 16 and 24 (N=8).	0.5 mL solution at 100 µg/mL for i.m. injection of MYM-V101.2;<br>0.2 mL solution at 250 µg/mL (100 µl/nostril) for i.n. injection of MYM-V101.3
	Placebo	A single i.m. injection of MYM-IRIVm solution at Weeks 0 and 8, and a single puff of MYM-IRIVn solution per nostril at Weeks 16 and 24 (N=4).	0.5 mL solution for i.m. injection of MYM-IRIVm;<br>0.2 mL solution (100 µL/nostril) for i.n. administration of MYM-IRIVn

Treatment will be administered as described in the Flowchart. In both panels, subjects will come to the clinical unit on the day of vaccination; there will be no hospitalization during this study. Administrations of the vaccine will take place in the clinical unit between 9:00 a.m. and 17:00 p.m.

I.m. injections. It should be performed in the anterolateral part of the leg, close to the inguinal lymph nodes that may drain antigens from the genital areas. Due to this proximity, it should favor higher local antibody production for optimized targeting of the vaginal and rectal mucosa. All i.m. vaccinations should be performed in the same leg.

I.n. administrations. It will be performed in both nostrils. Before administration, subjects will be asked to blow gently its nose. After carefully wiping the nose, the vaccine could be administered. It is strongly recommended not to blow the nose for at least 30 minutes following vaccine administration.

In the event that subjects have congestion prior to administration without fever (ex. environmental allergy(ies), there are two alternatives:

1. In case of a minor congestion, subjects will be asked to blow gently its nose, then rinse the two nostrils twice with a physiological solution and blow gently again. After carefully wiping the nose, the vaccine could be administered;
2. If it's a major congestion without fever, the administration can be postponed by 1-2 days and follow the procedure as above. However, if the subject suffers from an important congestion with fever, the vaccination must be postponed and synchronized with the next menses cycle. Vaccination can

only be postponed by one month once, when congestion continues after one month, the vaccination should take place according to the Flowchart in section 2.

Panel 2 will only be started when treatment in Panel 1 has proven to be safe and well tolerated. The time interval between start of Panels 1 and 2will be at least 1 week. The second i.n. vaccination will only be given when the first i.n. vaccination has been proven safe and well tolerated. Maximally five subjects may start vaccination on Visit 1, there should be at least 30 minutes between the vaccinations performed on one day.

Times of administration of vaccination will be recorded in the CRF.

5.3.5 Blinding and Unblinding

The investigator will be provided with a sealed envelope for each subject containing coded details of the treatment. The treatment code can only be broken in case of an emergency or if further treatment of the subject is dependent on the investigational treatment she has been receiving. The sponsor needs to be notified immediately when the clinical site breaks the code.

The date, time and reason for the code breaking must be recorded in the Code Breaking section in the CRF. In addition, the reason must be documented in the Adverse Event section of the CRF. The monitor should be notified immediately.

If the code is broken by the investigator or his/her staff, the subject must be withdrawn from the trial and must be appropriately followed. If the code is broken by the sponsor for safety reporting purposes, the subject may remain in the trial.

All envelopes, whether opened or sealed, will be collected at the end of the trial.

If ≥ 2 DLTs in a given panel are observed, then confirmation of the active treatment status of the subjects will be given by an external party in a blinded way. MTD is considered exceeded when ≥ 2 DLTs in a given panel while being on active treatment.

5.3.6 Drug Accountability

The investigator or his designees must maintain an adequate record of the receipt and distribution of all trial supplies using the Drug Delivery Form, the Drug Accountability Form and the Drug Return Form. These forms must be available for inspection at any time.

Supplies must be dispensed under the supervision of the Principal Investigator or investigator. Local regulations should be adhered to.

Unused treatment packages must be available at the site for drug accountability purposes and verification by the monitor. All used material must be appropriately discarded at the clinical site and unused treatment packages will be collected by the monitor and returned to the sponsor. This must be documented on the Drug Return Form. A written explanation regarding the disposition of missing products or their containers is required.

5.3.7 Storage

All trial treatments must be handled strictly in accordance with the protocol and the container labels. Storage and dispensing instructions and expiration dates are supplied with the investigational materials on delivery. Access to trial supplies should be restricted to designated trial personnel.

The trial supplies must be stored in a locked area in accordance with the storage conditions.

All supplies of MYM-V101 vaccines and MYM-IRIV placebos must be stored in the original vials delivered under the responsibility of Pevion Biotech AG and under the conditions indicated on the labels (keep cooled between 2-8°C).

The transport of vaccines from Pevion Biotech AG to the clinical unit in Belgium will be monitored with temperature loggers. The monitoring is under the responsibility of Pevion Biotech AG.

Temperatures logging at the investigational site should be performed. Should a deviation in storage conditions occur, the site must not further dispense the affected vaccines and must provide the monitor immediately with the following information:

- Date and duration of the deviation;
- Minimum temperature below the range and/or maximum temperature above the range that the product was exposed to.

Deviations in storage conditions will be evaluated by the Head of the QA department of Pevion.

The monitor will periodically check the supplies of trial treatments held by the investigator to ensure accountability (see also section 5.3.6) and appropriate storage conditions of all trial treatments used.

5.3.8 Compliance

All i.m. injections of vaccine are performed by the investigator at the unit or by the personnel dedicated to the trial. All i.n. administrations of vaccine at the unit are witnessed by the investigator or by the personnel dedicated to the trial.

If a subject's treatment is not according to the protocol, the investigator will take the necessary measures to ensure future adherence to the protocol.

5.3.9 Prior and Concomitant Therapy

Intake of medication prior to the first intended vaccination, which is listed in the exclusion criteria (see chapter 5.2.3) will prevent randomisation or will lead to exclusion from the study.

Use of concomitant medication(s) is planned as follows:

Allowed medications:

- Topical corticosteroid therapy (other than vaginal or rectal);
- Oral contraceptives;
- L-thyroxine;
- Paracetamol; the use of paracetamol may be permitted at a maximal daily dose of 3 x 500 mg and no more than 8 grams per week;
- In case of cutaneous reaction/rash and/or an allergic reaction, the use of cetirizine (Zyrtec®), levocetirizine (Xyzal®), topical corticosteroids or antipruritic agents in the recommended dosing scheme is permitted;
- In case of nausea, the use of antiemetics is permitted;
- In case of diarrhoea, the use of loperamide is permitted.

Disallowed medications:

- Nasal and inhalation corticosteroid therapy within 2 weeks prior to first administration;
- Topical (vaginal or rectal) corticosteroid therapy within 2 weeks prior to until 3 days after mucosal samplings;
- Vaginal creams and treatments, lubricants and rectal treatments within 3 days prior to mucosal samplings;

- Immuno-suppressive drugs;
- Systemic corticosteroid therapy;
- Anticoagulantia;
- Anti-tuberculosis medication;
- Progesterone and/or estrogen containing agents (except for oral contraception).

Checks for concomitant medication(s) will be performed at each visit to the clinical unit. Subjects will be asked to record intake of concomitant medication(s) in diary cards, which will be reviewed by the study personnel at each visit. All concomitant medication(s) is to be documented by brand, type (generic name, if applicable), dose, dose regimen and duration on the CRF. The Principal Investigator/investigator will then decide together with the responsible project manager of Kinesis Pharma BV whether the volunteer has to be excluded. For any concomitant therapy given as a treatment for a new condition or a worsening of an existing condition, the condition must be documented in the Adverse Event section of the CRF.

5.4 Assessments

5.4.1 Timing of Assessments

The exact timing of the assessments is presented in the Flowchart (see section 2).

Samples may be used by the sponsor for further exploratory work on immunological monitoring, protein analysis and biochemistry. No human DNA analysis will be performed.

5.4.1.1 Initial Subject Characteristics

Screening Visit

After the signing of the ICF, the investigator will check if the volunteer meets all the criteria to participate in the study. Therefore, the following assessments will be performed:

- Demographic data;
- Medical and surgical history, current medical condition(s), concomitant disease(s), any allergy(ies) or medication incompatibility(ies), and concomitant medication(s) (including OTC supplements);
- A gynaecological examination including vaginal swab to assess the presence of vaginitis;
- Smoking habits, alcohol consumption and use of recreational or narcotic drug(s);
- Physical examination, including height and weight;
- Electrocardiogram (ECG) and vital signs (body temperature, blood pressure and pulse);
- Blood samples: biochemistry, haematology, HIV (1 and 2) and hepatitis A, B and C screens, and for female volunteers of child-bearing potential a pregnancy test;
- Urinalysis;
- Explanation of restrictions.

The overall eligibility of the subject to participate in the trial will be assessed based on inclusion and exclusion criteria. The restrictions applicable during the study will be explained.

Baseline Visit

The baseline visit will be performed in the cycle prior to the cycle of first vaccination on Day 21-26 of the woman's cycle. In case of a short cycle, it should be taken at least two days prior to the start of menses. During this visit, baseline data will be collected and the following assessments will be performed:

- Physical examination;
- ECG and vital signs;
- Blood samples: serum (for antibodies), PBL (for B cell assessments), biochemistry and hematology for laboratory safety assessments (see section 5.4.3.4 for description);
- Urinanalysis;
- If subjects are eligible, mucosal secretions from rectum and vagina will be taken. For this procedure, a small sponge will be gently used to sweep the mucosa of rectum and vagina. During that procedure that lasts about 5 minutes for vaginal samplings and 12 minutes for rectal samplings, the sponge will absorb antibodies and fluid from the mucosa. The mucosa will be swept 4 times vaginally and twice rectally , at a 1 minute interval. Subsequently, this fluid will be extracted from the sponge in the laboratory and antibodies will be measured. Mucosal sampling should never be performed during the woman's menses due to the contamination of blood (for further details see section 0);
- Review of adverse events, concomitant medication and the restrictions.

Since the presence of natural mucosal antibodies varies during the menstrual cycle, see schedule in Addendum 14.2, the vaccinations should take place within 1 to 5 days after ovulation/mid cycle. Women can therefore only participate in this study if they have regular cycles lasting between 24-30 days.
As soon as all results of both the Screening and Baseline Visits are available, the investigator will review the eligibility of the subject and check the inclusion and exclusion criteria again, subsequently the investigator will inform the volunteer if she can participate in the study.

5.4.1.2 Treatment Period

Visit 1: Week 0 (1st vaccination)

After enrolment into the study, the subject needs to be at the clinical unit on the agreed day, which should match the woman's cycle between Day 1 and 5 after ovulation/mid-cycle. The following assessments will be performed prior to vaccination:

- Physical examination;
- Vital signs;
- Blood samples: serum (for antibodies), , biochemistry and hematology for laboratory safety assessments (see section 5.4.3.4 for description) and HIV (1 and 2) screen;
- Urinanalysis (including pregnancy test);
- Review of adverse events, intake of medication and the restrictions;

When the subject is without an acute disease, body temperature normal (≤ 37.5°C) for at least 24 hours prior to vaccination, the first vaccination will be performed under standardized conditions. Subjects will have to stay in the clinical unit up to 2 hours after vaccination to assess local and systemic adverse reactions. If necessary for medical reasons this in-house phase may be prolonged. If the subject is in a normal state of health, she may leave the clinical unit.

In case of an acute disease (body temperature > 37.5°C within 24 hours prior to vaccination), the vaccination may be postponed until the body temperature is normal (≤ 37.5°C) for at least 24 hours, in such a way that it still matches the woman's menstrual cycle between Day 1 to Day 5 after ovulation/mid-cycle. In case the vaccination cannot take place in accordance with the woman's cycle, the vaccination may be postponed once to match the next menstrual cycle. In case another acute disease might occur, vaccination should take place according to the Flowchart in section 2.

The use of diary cards will be explained to the subject and obligatory brought back to the clinical unit at each visit. From Day 1 to Day 7, subjects will be asked to complete diary cards daily.

Visit 2: Week 1 (7 days after the 1st vaccination)
The next visit (Visit 2) to the clinical unit is scheduled 7 days (6-8 days) after first vaccination. The following assessments will be performed:

- Physical examination;
- Vital signs;
- Blood samples: serum (for antibodies), PBL (for B cell assessments), biochemistry and hematology for laboratory safety assessments (see section 5.4.3.4 for description);
- Review of diary cards, corrections and clarifications will be added if necessary;
- Provision of a new diary card to record the occurrence of AEs and intake of concomitant medication for the 2-8 weeks after vaccination;
- Review of AEs, intake of medication and the restrictions.

The study team will review the diary cards for completeness and legibility of the data and the diary cards will be discussed with the subjects, if necessary.

Visit 3: Week 4
Visit 3 is scheduled 4 weeks (± 2 days) after first vaccination. The following assessments will be performed:

- Physical examination;
- Vital signs;
- Blood sample: serum (for antibodies) and biochemistry and hematology for laboratory safety assessments (see section 5.4.3.4 for description);
- Urinanalysis;
- Review of diary cards, providing a new diary card for the coming 4 weeks;
- Review of AEs, intake of medication and the restrictions.

Visit 4: Week 8 (2nd vaccination)
At Week 8, Visit 4 will take place for the second vaccination. It should be performed 8 weeks after Visit 1, although it is important that it matches the woman's menstrual cycle between Day 1 to Day 5 after ovulation/mid-cycle. All assessments as described for Visit 1 will take place with the exception that **no** blood samples for PBLs will be taken. In addition, diary cards will be reviewed and a new diary card for the coming week will be provided.

In case of an acute disease, the instructions as described at Visit 1 should be followed.

Visit 5: Week 9 (7 days after the 2nd vaccination)
Visit 5 will be performed 7 days (6-8 days) after the second vaccination and will be the same visit as Visit 2. All assessments as described for Visit 2 will take place.

Visit 6: Week 16 (3rd vaccination)
The third vaccination will be scheduled at Visit 6. It should be performed 16 weeks after Visit 1, although it is important that it matches the woman's menstrual cycle between Day 1 to Day 5 after ovulation/mid-cycle. All assessments as described for Visit 1 will take place, except for blood sample for PBLs and HIV (1 and 2) screen. In case of an acute disease, the instructions as described at Visit 1 should be followed.

Visit 7: Week 17 (7 days after the 3rd vaccination)
Visit 7 will be performed 7 days (6-8 days) after the third vaccination. Assessments of Visit 7 will be the same as described for Visit 2, in addition the following samples will be taken:

- Collection of mucosal fluids from vagina only.

Visit 8: Week 24 (the 4th and last vaccination)
The last vaccination will take place at Visit 8. It should be performed 24 weeks after Visit 1, although it is important that it matches the woman's menstrual cycle between Day 1 to Day 5 after ovulation/mid-cycle. All assessments as described for Visit 6 will take place at this visit. In case of an acute disease, the instructions as described at Visit 1 should be followed.

Visit 9: Week 25 (7 days after the 4th vaccination)
Visit 9 will be performed 7 days (6-8 days) after the fourth vaccination. This visit is also the first Follow-up Visit. At this visit, assessments will be the same as described for Visit 6, except for pregnancy test. In addition, the following assessment will be performed:

- Collection of mucosal fluids (from rectum and vagina);
- ECG;
- Blood samples: PBL (for B cell assessments).

5.4.1.3 Follow Up

Visit 10: Week 29 (Close Out Visit, ending the study participation).
This visit will take place 5 weeks after the 4th vaccination (on Day 21-26 of the cycle), and will be the last visit of the study. As the study reached its completion, it will be checked that the subject leaves the study in **healthy state** and that no clinically relevant changes, sign of illness or health damage are present due to the vaccination, as compared to start of the study. Assessments of Visit 10 will be performed as in Visit 1. Additional assessments will be performed:

- Collection of mucosal fluids (from vagina only);
- ECG;
- Blood sample for hepatitis tests;
- Weight;
- Collection and review of the last diary cards, which can be discussed with the subject, if necessary;
- Review of AEs and concomitant medication will be questioned and the restrictions will be checked. For Follow-up of AEs which remains unresolved at the time point of last visit, see section 5.4.3.1.5. If the subject refuses to follow the instructions of the investigator, the latter is released from responsibility.

5.4.1.4 Early Withdrawal

A visit should be performed in case a subject was exposed to any investigational study treatment. In case of dropout, except in case of withdrawal of informed consent, subjects will be advised to come to the clinical unit for a visit at the time of dropout or as soon as possible within one week after discontinuation. The following assessments will be performed:

- Physical examination;
- Vital signs;
- ECG;
- Weight;
- Blood samples: serum (for antibodies), biochemistry and hematology for laboratory safety assessments (see section 5.4.3.4 for description) and HIV (1 and 2) and Hepatitis (A, B and C) screens;
- Urinanalysis including pregnancy test (for women of childbearing potential only);

- Review of AEs, intake of medication and the restrictions;
- Collection and review of the last diary cards, which can be discussed with the subject, if necessary.

5.4.2 Efficacy Evaluations

Since the primary objectives of this study are safety assessments, the evaluation of these parameters will be described in section 5.4.3.2. The continuous assessment of AEs throughout the trial will be described in section 5.4.3.1.

5.4.2.1 Immunological Monitoring

Bioanalysis will be performed by the bioanalytical laboratories contracted by the Sponsor. Antibody concentrations, immunoglobulin isotyping and functional assays will be determined, using different GLP (Good Laboratory Practices) validated or standardized techniques. A description of the bioanalytical methods used will be included with the final report. Since the amount of collected material from secretions might be a limiting factor. Depending on the amount of available antibodies, some or all functional immunological assays might be performed on the collected materials, as part of the ancillary study. Additional analyses on immunological parameters might be performed depending on the amount of available material. Tests should be performed in the following priority order:

A. For secondary objective:

- Quantitative analysis on blood: antibody measurements.

B. For Ancillary study:

- Quantitative analysis of mucosal samples: antibody measurements;
- *In vitro* transcytosis assays with mucosal samples (if in sufficient quantity and quality);
- *B cell* phenotyping for mucosal homing with PBL samples;
- *In vitro* neutralization assays with blood and mucosal samples (if in sufficient quantity and quality).

5.4.2.1.1 Immunological Monitoring on Serum

- **Quantification of blood antibodies against P1 peptides.** It will be assessed by an improved ELISA test called Imperacer® [17] that has been developed by Chimera Biotech (Dortmund, Germany). This technology uses antibodies conjugated to small DNA fragments that can be amplified by PCR. The Imperacer® has been adapted for human blood and mucosal samples, allowing use of very small amount of the collected material, with a sensitivity of about 100 to 1000-fold superior to standard ELISA tests.

  Chimera Biotech will conduct sample analysis under GLP compliance. These assays are intended for the determination of the ratio of IgG and IgA in a given sample, either in regard of specific IgG/total IgG and specific IgA/total IgA. Capture-antibody anti-human IgG or IgA will be used for quantifying total antibodies, while the lipidated P1 peptides will be used as capture-antigen for determining the amount of specific antibodies.

- *In vitro* neutralization assays performed on blood samples will be performed as described below in section 0.

- B cell phenotyping for mucosal homing with PBL samples will be performed by Dr. Fabienne Anjuère (INSERM UMR 721, Faculty of Medicine Pasteur, Nice, France). The frequency of IgA and IgG memory and effector B cells before and following vaccination will be assessed by flow cytometry analysis

following multiparametric stainings with different fluorescent antibodies specific for IgG, IgA, CD19 (B cell marker), CD20 (B cell marker), CD27 (memory marker), CD38 (activation marker expressed by plasmablasts), α4β7 integrin (mucosal marker). Memory B cell cells are Ig+CD19+CD20+CD27+ as previously described by Crotty *et al*.[18]. Mucosal antibody secreting cells are alpha4beta7+CD38+Ig+CD19+ [Quiding-Jarbrink *et al*.[19] and personal communication from F. Anjuère and C. Czerkinsky].

5.4.2.1.2 Immunological Monitoring on Secretions

- **Quantification of mucosal antibodies from vaginal and rectal secretions against P1 peptide.** It will be assessed by the Imperacer® technology developed by Chimera Biotech, as described above in section 5.4.2.1.1.
- ***In vitro* Neutralization assays**. These assays will be performed on vaginal secretions. This technique evaluates the potential of antibodies to block HIV-1 infection. It has been set up and standardized in the laboratory of Dr. Lucia Lopalco (San Raffaelle Hospital, Milan, Italy), a world expert on mucosal antibodies in HIV-1 field. In order to evaluate the presence of neutralizing antibodies in human specimen, the neutralizing activity of IgA and IgG from biological fluids, including serum and vaginal secretions will be studied, by using a validated and standardized assay[21]. Serum and vaginal secretions will be used as source of antibodies. IgA and IgG will be purified by chromatography automatic system (DuoFlow, Biorad). The neutralizing assay takes advantage of a genetically engineered cell line (TZM-bl, JC53-bl obtained through the NIH AIDS Research and Reference Reagent Program, USA) that contains a stably embedded Tat-responsive Luc reporter gene for highly sensitive and reproducible measurements of virus entry after a single round of infection in a high throughput format. This assay may be used with molecularly cloned, Env-pseudotyped viruses for greater reagent stability and traceability. One of the more significant technical hurdles overcome by TZM-bl cells is their high permissiveness to infection by most strains of HIV-1, regardless of genetic subtype and biologic phenotype. Neutralizing antibodies will be assessed by using molecularly cloned. Env-pseudotyped viruses generated by transfection in 293T cells and titrated for infectivity in TZM-bl cells. Env-pseudotyped viruses (obtained through the NIH AIDS Research and Reference Reagent Program, USA) provide significant technical and scientific advantages, including greater reagent stability and traceability, improved assay reproducibility and an ability to analyze antibody specificities in relation to a precisely known Env sequence, compared to uncloned viral quasispecies, which can change in relative proportion and complexity upon repeated passages. Cells will be plated in 96-well plates. The samples will be serially diluted in the preferred range in complete cell media to a final volume of 120 µL. Serum and/or secretions or purified IgG and IgA from HIV-negative controls or pre-vaccinated subjects will be included as controls every time the assay will be performed to ensure the validity of the results. To the diluted samples, a mixture containing the desired virus and DEAE-dextran (to aid in infectivity) will be added and allowed to incubate. After the incubation, the sample-virus mixtures will be added to each well of plated cells and allowed to incubate; the media will be then removed and lysing buffer added to the cells. The level of infectivity is quantified by measuring luminescence activity in the lysed cells, using a specific counter system (Top Count, Packard).
- ***In vitro* Transcytosis assays.** These assays will be performed on vaginal secretions. This technique evaluates the potential of antibodies to prevent HIV-1 translocation across a monolayer of polarized cells (mimicry of the mucosal epithelium). Transcytosis assays have been standardized in the laboratory of Dr. Morgane Bomsel (Cochin Institute, Paris, France), a world expert on HIV-1 entry across mucosa. HIV-1 transcytosis will be performed as previously described[18]. Briefly, the endometrial cell line HEC-1 or intestinal cell line HT-29 clone 19[19] are each grown as a tight,

polarized monolayer for seven to ten days, respectively, on a permeable filter support (0.45 µm pore size) forming the interface between two independent chambers; the upper one bathing the apical surface of the epithelial monolayer and the lower one bathing the basolateral surface. Prior to transcytosis experiment, epithelial cells are washed and further incubated in culture medium. Secretion samples (1/10 dilution) and a positive control (a monoclonal IgG (100 ng) against the gp41 2F5 epitope (from the NIH AIDS Reagent program), are pre-incubated with HIV-infected PBMC or NDK-CEM cells in culture medium. Then, HIV-1 infected cells pre-incubated or not with antibodies are added to the apical chamber. Contact between HIV-1 infected cells and the epithelial cell monolayer results in rapid budding of the HIV-1-virions[20], followed by HIV-1 particle internalization and transcytosis from the apical to the basolateral pole of the epithelial cell monolayer. After 2 h, inhibition of transcytosis by antibodies is determined by detection of the p24 protein in the basolateral medium by commercial ELISA (Coulter, Villepinte, France). During the 2 h of infected-cell contact with epithelial cells, the barrier function of the epithelial monolayer remains intact, precluding penetration of HIV-1 infected cells in the monolayer or translocation of HIV-1 infected cells in the basolateral chamber[18]. Similarly, in the presence of IgA or IgG in the apical chamber, the transepithelial resistance which is routinely measured, is not perturbed and remains constant throughout the experiment[18].

5.4.2.2 Sample Collection and Handling

All laboratory samples will be taken according to the flowchart, and processed, handled and identified according to the laboratory manual, which will be provided before the start of the trial. Exact time points of sampling will be recorded in the appropriate section of the CRF.

5.5.2.2.1 Secretion Samplings

All vaginal and rectal secretion samplings will preferably be performed by the same physician.

Prior to mucosal samplings, the investigator should check by clinical investigation if the subject is free of infection at the site of sampling. In case of infection, the mucosal sampling can be postponed. For baseline, the visit can be postponed by 1-3 days, but should be at least 2 days prior to menses, or next month of the cycle. If the subject has already been vaccinated, <u>sampling must remain</u> as scheduled or postponed by 1-2 days, but it should be noted that this sample might be “unacceptable”.

For vaginal secretions, it is IMPORTANT to perform the two - four samplings into two different regions:

- The first Weck-Cel® sponge(s) must be introduced into the upper part of the vagina (avoid touching the exocervix);
- The second sampling(s) must be done in the lower part of the vagina. Because the first sampling may potentially cause light bleeding, it is important that the second sampling is done distant from the first sampling site to avoid blood contamination. Any contamination due to bleeding would render the material useless because mucosal antibodies would be contaminated by antibodies originating from the blood.

At Baseline, the physician should do 4 sequential vaginal harvestings at a 1 minute interval using Weck-Cel sponges®[16] in order to obtain at least 120 µg of total mucosal IgA antibodies. To collect vaginal secretions, a Weck-cel® sponge should be placed gently in the vagina and allowed to passively adsorb secretion for approximately 20-30 seconds.

For the remaining vaginal secretion samplings (Visits 7, 9 and 10), the physician should do 2 sequential harvestings at 1 minute interval, using Weck-Cel sponges®[16] in order to obtain at least 60 µg of total mucosal IgA antibodies.

For rectal samplings, women will be positioned lying on their sides on an examination table. A thin film lubricating jelly will be applied with sterile gauze around the smooth end of the applicator pipette and on the anus of the subject. The smooth end of the applicator will be gently and slowly inserted 6 cm into the rectum. Once in place, the flexible transfer pipette (and hence, the sponge) will be held in place while the applicator will be slowly pulled back 1.5 cm to expose the sponge to the rectal mucosal surface completely as described by Kozlowski[16]. After 5 minutes, the sponge should be pulled back into the applicator by slowly pulling the transfer pipette out a distance on the applicator. The applicator with sponge will be removed from the rectum. The sponge will subsequently be pulled out of the applicator and separated from the transfer pipette. The sponge will be quickly returned to its tube, firmly capped, placed on ice, and then worked up according to the laboratory manual.

For all rectal secretion samplings (Baseline and Visit 9), the physician should do 2 sequential harvestings at 1 minute interval, using Weck-Cel sponges®[16] in order to obtain at least 60 µg of total mucosal IgA antibodies.

5.4.3 Safety Evaluations

5.4.3.1 General

5.4.3.1.1 Adverse Event Definitions

Adverse Event (AE)

Any untoward medical occurrence in a patient or clinical investigation subject treated with a pharmaceutical product and which does not necessarily have a causal relationship with this treatment. An adverse event can therefore be any unfavorable and unintended sign (including an abnormal laboratory finding), symptom or disease temporally associated with the use of a medicinal (investigational) product, whether or not related to the medicinal (investigational) product (International Conference on Harmonization - ICH E6; 1.2).

Serious Adverse Event (SAE)

Any untoward medical occurrence that at any dose:

- Results in death;
- Is life threatening;
- Requires inpatient hospitalization or prolongation of existing hospitalization;
- Results in persistent or significant disability/incapacity;

or

- Is a congenital anomaly/birth defect (ICH E6; 1.50).

<u>Notes</u>

- Medical and scientific judgment should be exercised in deciding whether expedited reporting is appropriate in other situations, such as important medical events that may not be immediately life threatening or result in death or hospitalization, but may jeopardize the subject or may require intervention to prevent one of the outcomes listed in the definition above.
- Hospitalizations that were planned prior to the signing of informed consent, and where the underlying condition for which the hospitalization was planned has not worsened, will not be

considered serious adverse events. Any adverse event that results in a prolongation of the originally planned hospitalization is to be reported as a new serious adverse event.

Unexpected Adverse Event

An adverse reaction, the nature or severity of which is not consistent with the applicable product information (i.e., Investigator's Brochure for an unapproved investigational product or package insert/summary of product characteristics for an approved product).

Life Threatening

Any event in which the subject was at risk of death at the time of the event; it does not refer to an event, which hypothetically might have caused death if it were more severe.

Associated With the use of the Drug

An adverse event is considered associated with the use of the drug if the attribution is possible, probable or very likely.

5.4.3.1.2 Classification of Intensity of Adverse Events

The different categories of intensity (severity) are characterized as follows:

MILD: The AE is transient and easily tolerated by the subject.

MODERATE: The AE causes the subject discomfort and interrupts the subject's usual activities.

SEVERE: The AE causes considerable interference with the subject's usual activities.

5.4.3.1.3 Classification of Causality of AEs to the Investigational Product

Every AE experienced during the clinical trial must be evaluated for its relationship to the investigational product administered by the investigator. Causal relationships of SAEs will also be assessed by the sponsor. Causality rating by the investigator will be performed by introspection. Causality assessment by the sponsor will be performed by the Naranjo causality scale[24]. Causality can be assessed as either:

Not Related

An adverse event, which is not related to the use of the investigational product.

Unlikely

An adverse event for which an alternative explanation is more likely, i.e., concomitant drug(s) or concomitant disease(s), and/or the relationship in time suggests that a causal relationship is unlikely.

Possible

An adverse event which might be due to the use of the investigational product. An alternative explanation, i.e., concomitant drug(s) or concomitant disease(s), is inconclusive. The relationship in time is reasonable; therefore the causal relationship cannot be excluded.

Probable

An adverse event, which might be due to the use of the investigational product. The relationship in time is suggestive, i.e., confirmed by dechallenge. An alternative explanation is less likely, i.e., concomitant drug(s) or concomitant disease(s).

Very Likely

An adverse event, which is listed as a possible adverse reaction and cannot be reasonably explained by an alternative explanation, i.e., concomitant drug(s) or concomitant disease(s). The relationship in time is very suggestive (i.e., it is confirmed by dechallenge and rechallenge).

5.4.3.1.4 Reporting of AEs

AEs will be collected from the signing of ICF onwards. Occurrence of AEs will be reported in the Adverse Event section of the CRF. Subjects will record the occurrence of AEs during the study on diary cards. AEs assessed as clinically relevant by the investigator should be transferred from the diary to the CRF

All AEs still ongoing at the end of the treatment will be followed until satisfactory resolution (i.e., value back to baseline value) or stabilization (to be agreed upon in collaboration with the sponsor).

New AEs reported during the follow-up period of the trial will be followed until the AE has been resolved or a reasonable explanation for its persistence is found; in case of drug-related and clinical relevant AEs, every effort has to be made to follow-up trial subjects in order to determine the final outcome.

SAEs occurring within the clinical trial (between signing of ICF and last Follow-Up Visit) will be reported. Any SAEs with at least a possible relationship to the study medication occurring after the end of the trial must be reported, and will be handled by the sponsor.

The start date of the SAE documented on the Serious Adverse Event form should be the date the AE first fulfilled any serious criterion. If a change in severity is noted for the existing AE, it must be recorded as a new AE. If a worsened AE meets the criteria for a SAE, the start date of the SAE must be the same as the start date of the worsened AE.

The cause of death of a subject in a clinical trial, whether the event is expected or associated with the investigational agent, is a SAE.

Any SAE or pregnancy should be reported immediately by telephone +31-(0)6-305 15 101 after the investigator becoming aware of the event. These preliminary reports will be followed within 24 hours by detailed descriptions that will include a completed SAE form, copies of hospital case reports, autopsy reports, and other documents, when requested and applicable.

**Name**	**Mouton's Safety Consultancy**
**Address**	**Nieuwstraat 11A, 5371 AH Ravenstein, The Netherlands**
**Phone**	**+31-76-54 80 680**
**E-mail**	**safety@kinesis-pharma.com (PDF formats of SAE reports only)**

Standard SAE report templates can be found in the ISF. Minimal information should include:

- An identifiable subject or patient (eg. Study subject code number);
- An identifiable reporting source;
- All related adverse events;
- The suspect medicinal product.

If / when supplementary information is available, a follow-up SAE Report Form must be completed by the site and sent as PDF within 24 hours to safety@kinesis-pharma.com.

Once sent, the SAE form and accompanying documentation should be placed in the SAE section of the investigator's file. If supplementary information on a SAE has to be sent, the SAE form has to be used, marked as "follow-up report".

Pregnancies occurring during clinical trials are considered immediately reportable events. They must be reported as soon as possible using the Serious Adverse Event Form. The outcome of the pregnancy must also be reported to the Drug Safety Officer.

Kinesis will assume responsibility for appropriate reporting of SAEs to Regulatory Authorities. According to local regulations, Kinesis will also report all SAEs that are unlisted and associated with the use of the drug (SUSAR) to the investigators and Independent Ethics Committee (IEC). For reported deaths, the investigator should supply the sponsor and the IEC with any additional requested information (i.e., autopsy reports and terminal medical reports).

After termination of the clinical trial (last subject last visit in the trial), any unexpected safety issue that changes the risks benefit analysis and is likely to have an impact on the subjects who have participated in it, should be reported as soon as possible to the competent authority(ies) concerned together with proposed actions.

5.4.3.1.5 Checks for AEs

AEs will be checked at every visit and reported from signing the ICF onwards until the last trial-related visit. Severity and drug relationship of AEs towards MYM-V101 vaccine will be recorded. AEs, which are recorded on diary cards, will be reviewed by the study team on every visit and discussed with the subject if necessary.

All subjects will come for a Follow-Up Visit, scheduled 7 days after the last administration of vaccine. A second posttreatment visit is scheduled 5 weeks after the last vaccination, for a final trial-related assessment of possible AEs (see Flowchart). All AEs still ongoing at the end of the treatment will be followed until satisfactory resolution (i.e., value back to baseline value) or stabilization (to be agreed upon in collaboration with the sponsor).

Special attention will be paid to those subjects who discontinue the trial for an AE, or who experience a severe AE, or a SAE. In case of dropout, subjects will come, if consent not withdrawn, for a Follow-Up Visit at the moment of dropout or as soon as possible within one week after discontinuation.

5.4.3.2 Risks Related to the Participation in the Clinical Trial

5.4.3.2.1 Procedure Related Risks

Vein puncturing for the purpose of blood sampling may be accompanied by some mild pain. There is a small risk of bleeding, bruising or infection at the site of injection. In addition, the subject might feel dizzy during the blood sampling procedures. On 4 different days, mucosal samples will be taken from the vagina and the rectum. Due to this procedure there might be some spotting that will disappear within a few days. There is also a small risk of a genital infection following the sampling, for which appropriate treatment is available. On 4 occasions the subjects will be vaccinated. In case of i.m. injection, the following AEs were reported for other marketed vaccines: pain, redness, swelling, indurations and ecchymosis[5-9]. All these AEs were mild to moderate in intensity, transient and disappeared within 3 days after vaccination. I.n. vaccination resulted in the following adverse reactions: fever, fatigue, nausea,

rhinitis, stuffy nose and rhinopharyngitis[15]. It should be mentioned however that these adverse reactions were observed with a virosomes-based vaccine containing an additional adjuvant, which is not present in the vaccine formulation used in this study. Therefore, less adverse reactions when compared to other vaccines are expected in this study.

The total volume of blood withdrawn during the entire study will be approximately 400 mL over a time period of approximately 34 weeks, which is less than the volume of a normal blood donation on one occasion (450 mL). No safety related risk is expected from this blood withdrawal.

5.4.3.2.2 Risks Related to the Investigational Vaccine

Despite that at least four virosomes-based vaccines have provided good safety data in human, the proposed MYM-V101 has not been used in humans before. Therefore adverse events from human studies are not yet known. Meanwhile, results from pre-clinical studies on monkeys have indicated that the vaccine was well tolerated by animals (no changes in behavior or signs of pain)[10-11]. Among the 3 studies conducted from April to October 2008 (rats and rabbits) for investigating the toxicology of the MYM-V101, no serious side effects were reported upon macroscopic examination and histological analyses of the various organs[12-14]. AEs reported on the use of virosomes (the carrier only) are: headache, malaise and flu-like symptoms, muscle pain and tiredness[4]. In very rare cases a severe allergic reaction can occur (anaphylactic shock) after vaccination, for which appropriate treatment is available at the clinical unit. Vaccination in general does not influence the capacity of reaction.

5.4.3.3 Specific Toxicities

5.4.3.3.1 Occurrence of Local and Systemic AEs

Subjects will have to stay in the clinical unit up to 2 hours after each vaccination for assessment of local and systemic AEs by the study team. The site of i.m. injection will be inspected and pain, redness and swelling will be assessed. After i.n. administration, nasal congestion, running nose, impaired smelling, and headache will be assessed. The study team will record the occurrence of the local and systemic AEs in the CRF directly. Subsequently subjects will be given diary cards to register the occurrence of AEs at home. Diary cards for the first seven days after vaccination will be preprinted diary cards for recording of solicited adverse experiences following the administration of the vaccine. In addition, reaction measurement gauges (after i.m. administration) and a digital thermometer to record daily body temperatures from Day 1 to Day 7 post-vaccination and, in subsequent days, if the subject appears ill, will be distributed. Local solicited adverse experiences (pain, swelling, redness after i.m. administration and nasal congestion, running nose, impaired smelling, and headache after i.n. administration) and axillary body temperature will be recorded from Day 1 to Day 7 after each vaccination. Any adverse experiences and concomitant medications taken will be recorded from Day 1 to Day 56 after each vaccination. Diary cards will be reviewed by the study team for completeness and legibility, results from solicited AEs will be copied into the CRF, the original diary card will be become part of the source data. AEs assessed as clinically relevant by the investigator will be transferred to the CRF.

After i.m. administration, the intensity of local pain and unsolicited symptoms and after i.n. administration the intensity of nasal congestion, running nose, impaired smelling, and headache will be classified as follows:

0. Absent;
1. Mild: the pain/adverse experience is easily tolerated and impairs the functional level of the subject only slightly, if at all;

2. Moderate: the pain/adverse experience is sufficiently discomforting to interfere with daily activities;
3. Severe: the pain/adverse experience prevents normal everyday activities.

The largest diameter of redness or swelling will be scored as:

Redness or swelling with a diameter less than or equal to 5 mm is not relevant and should not be recorded,

1. From 5 to 20 mm;
2. From 20 to 50 mm;
3. Larger than 50 mm.

Following systemic symptoms should be registered on diary cards as well: tiredness, headache, gastro-intestinal complaints, malaise and muscle pain. The intensity of these symptoms should be classified as follows:

0. Absent;
1. Mild: the symptom is easily tolerated and impairs the functional level of the subject only slightly, if at all;
2. Moderate: the symptom is sufficiently discomforting to interfere with daily activities;
3. Severe: the symptom prevents normal everyday activities.

Other local and systemic symptoms should be registered in diary cards at the moment of occurrence. Intensity rating of these systemic symptoms should be classified as follows:

1. Mild: the symptom is easily tolerated and impairs the functional level of the subject only slightly, if at all;
2. Moderate: the symptom is sufficiently discomforting to interfere with daily activities;
3. Severe: the symptom prevents normal everyday activities.

The subjects will be instructed to contact the clinical unit immediately if any SAE occurs. An SAE is defined as any experience that suggests a significant hazard to the subject’s health. The relationship of adverse events to the vaccination will be assessed by the investigators according to the AE definitions as described in section 5.4.3.1.2.

5.4.3.3.2 Occurrence of Clinically Significant Hematological and Biochemical Abnormalities

Safety samples to assess the occurrence of clinically significant hematological and biochemical abnormalities will be taken according to the flowchart (see section 2). The following parameters will be measured at Baseline and at Visit 1 to Visit 9:

Hematology

Hemoglobin, hematocrit, red blood cell (RBC) count, the RBC parameters mean corpuscular hemoglobin (MCH), mean corpuscular hemoglobin concentration (MCHC), and mean corpuscular volume (MCV), white blood cell (WBC) count, WBC differential (neutrophils, lymphocytes, monocytes, eosinophils, basophils) and platelet count.

Biochemistry

Aspartate aminotransferase (AST), alanine aminotransferase (ALT), creatinine, creatinine clearance (calculated using the Cockcroft-Gault equation) and C-reactive protein.

Laboratory abnormalities assessed as clinically significant abnormalities by the investigator should be recorded as AE. The clinical significant abnormalities, recorded as AEs will be assessed regarding intensity and classification of causality. The original laboratory printouts will be reviewed, signed and dated by the investigator. The original printouts will become part of the CRF.

5.4.3.4 Clinical Laboratory Tests

Blood samples for biochemistry and hematology, and urine samples for urinalysis will be taken according to the flowchart. All laboratory samples will be handled according to the laboratory manual.

The laboratory reports must be filed with the source documents. The laboratory report must be interpreted, signed, and dated by the investigator. Any clinically relevant abnormalities occurring during the trial, from signing the ICF onwards, must be recorded in the Adverse Event section of the CRF.

5.4.3.4.1 Haematology

The following assessments will be performed at Screening, at early withdrawal and at the second Follow-Up Visit (Visit 10):

Hemoglobin, hematocrit, RBC count, the RBC parameters MCH, MCHC and MCV, WBC count, WBC differential (neutrophils, lymphocytes, monocytes, eosinophils, basophils) and platelet count.

5.4.3.4.2 Biochemistry

The following assessments will be performed at Screening, at early withdrawal and at the second Follow-Up Visit (Visit 10):

AST, ALT, creatinine, creatinine clearance (calculated using the Cockcroft-Gault equation) and C-reactive protein.

5.4.3.4.3 Additional Assessments

The following additional assessments will be performed:

- At Screening: a serum pregnancy test (women of childbearing potential only);
- At Screening: a vaginal swab (bacteria (Neisseria gonorroea, streptococci, Gardnerella) and yeasts (candida)) to assess the presence of vaginitis;
- At Screening, at Visit 10 and in case of early withdrawal: hepatitis A, B, and C test (hepatitis A antibody IgM, hepatitis B surface antigen, and hepatitis C virus antibody, respectively);
- At Screening, Visit 1, Visit 4, Visit 10 and in case of early withdrawal: an HIV-1 and -2 test;
- Prior to each vaccination, at Visit 10 and in case of early withdrawal: a urine pregnancy test (women of childbearing potential only).

5.4.3.4.4 Urinalysis

A midstream urine sample must be provided for urinalysis by dipstick for protein, glucose, and occult blood. If abnormal, microscopic examination for WBC, RBC, and casts will take place. The assessments will be performed at Screening, Baseline, Visit 1, Visit 3, Visit 4, Visit 6, Visit 8 to Visit 10 and in case of early withdrawal.

5.4.3.5 Cardiovascular Safety

Clinically relevant cardiovascular abnormalities occurring during the trial must be recorded in the Adverse Event section of the CRF.

5.4.3.5.1 Vital Signs

Systolic and diastolic blood pressure (SBP, DBP) and pulse rate (PR) (supine after at least 5 minutes rest) will be recorded according to the flowchart.

Blood pressure and pulse rate measurements will be assessed with a completely automated device consisting of an inflatable cuff and an oscillatory detection system. All values will be registered on a built-in recorder so that measurements are observed independent.

Body temperature should be measured orally according to the flowchart.

5.4.3.5.2 ECG

An ECG (supine after at least 5 minutes rest) will be recorded according to the flowchart.

Twelve-lead ECGs will be recorded at a paper speed of 25 mm/s so that the different ECG intervals (RR, P, PQ, QRS, QT) will be measured. ECGs will be judged by the investigator.

5.4.3.6 Physical Examination

To evaluate the subject’s eligibility, a physical examination including body temperature will be performed at Screening Visit. In addition, a physical examination including body temperature will be performed at the study visits throughout the trial. At Screening, a gynecological examination will be performed to assess the presence of vaginitis.

To obtain the actual body weight, subjects must be weighed lightly clothed. The height should be measured barefooted at the Screening Visit.

Any clinically relevant changes occurring during the trial must be recorded on the Adverse Event section of the CRF.

5.5 Statistical Methods Planned and Determination of the Sample Size

Efficacy statistical analysis, statistical demographic, and statistical analysis for safety and tolerability will be performed by a qualified vendor under the supervision/responsibility of Kinesis Pharma BV.

5.5.1 Determination of Sample Size

No formal power calculation is performed. A total of 24 subjects, divided over 2 panels of 12 subjects each, are deemed sufficient to meet the objectives of this trial.

In both panels, 8 subjects will be vaccinated with active MYM-V101 and 4 subjects will receive the MYM-IRIV placebo. At least 8 subjects per panel should complete treatment: 6 subjects receiving the active vaccine and 2 subjects receiving the placebo vaccine.

5.5.2 Statistical Analyses

5.5.2.1 Initial Subject and Disease Characteristics

All demographic (age, height, weight, BMI, ethnic origin) and other initial subject characteristics (smoking habits, physical examination, medical and surgical history, concomitant diseases) will be tabulated and analyzed descriptively.

5.5.2.2 Efficacy Analyses

5.5.2.2.1 Immunological Monitoring

Immunological data as described for each time point will be analyzed separately.

Descriptive statistics (minimum, maximum, median, geometric mean, arithmetic mean and quartiles) will be computed for each immunological parameter and each time point, separately for the 2 vaccination groups.

For each volunteer, the ratio of the immunological parameter to that assessed at Baseline (Week -3) will be computed. Descriptive statistics of these ratios (minimum, maximum, median, geometric mean, and quartiles) will be computed for each immunological parameter and each time point, separately for the 2 vaccination groups.

Wilcoxon test will be used.

5.5.2.3 Safety

5.5.2.3.1 Occurrence of Local and Systemic AEs

Diary card data regarding the solicited adverse experiences following the administration of the vaccine will be evaluated as follows: Descriptive statistics (minimum, maximum, median, geometric mean, arithmetic mean and quartiles) computed for each diary parameter and each time point, separately for the 2 vaccination groups.

5.5.2.3.2 Adverse Events

All events will be coded using the Medical Dictionary for Regulatory Activities (MedDRA). Type and incidence of all AEs will be tabulated overall and per treatment group. Severity and drug relatedness of all reported AEs will be tabulated.

Special attention will be paid to those subjects who have discontinued the trial for an AE, or who experienced a severe AE or a SAE.

The incidence of AEs under active treatment will be compared with the incidence of AEs under placebo.

5.5.2.3.3 Clinical Laboratory Tests

For the clinical laboratory data, descriptive statistics (actual values and changes from baseline or change over time) will be generated for all tests performed. Graphical presentation of changes in laboratory tests will be made as applicable. Laboratory abnormalities will be determined according to the normal ranges of the clinical laboratory. Laboratory abnormalities will be tabulated per panel and treatment.

5.5.2.3.4 Cardiovascular Safety

The effects on cardiovascular parameters will be evaluated by means of descriptive statistics and frequency tabulations. These tables will include changes from baseline to allow detection of relevant changes in individuals.

Abnormalities as defined in section 14.1, Addendum 1 will be tabulated.

5.5.2.3.5 Other Safety Parameters

Physical examination results including body temperatures will be tabulated and abnormalities will be listed.

6 Trial Closure Considerations

The sponsor reserves the right to close an investigational site or terminate the trial at any time for any reason. In case of an early termination of the trial or temporary halt by the sponsor, the IEC should be notified within 15 calendar days, including a detailed written explanation of the reasons for the termination/halt.

When the trial ends in a particular country, the sponsor will submit an end of trial declaration, to both the IEC and regulatory authority for that particular country by using the “Declaration of the end of trial form”. The declaration will be submitted within 90 days of the end of the trial.

The end of trial declaration will be submitted a second time to the regulatory authorities and IEC after the complete trial has ended in all participating centers, in all countries. This notification will also be submitted within 90 days of the end of the trial.

Reasons for the closure of an investigational site or termination of a trial by the sponsor may include but are not limited to:

- Successful completion of the trial at the center;
- The overall required number of subjects for the trial has been recruited;
- Failure of the investigator to comply with the protocol, ICH-GCP guidelines or local requirements;
- Safety concerns;
- Sufficient data suggesting lack of efficacy;
- Inadequate recruitment of subjects by the investigator.

7 Trial Materials

7.1 Investigational Products

The investigator acknowledges that the drug supplies are investigational and as such must be handled strictly in accordance with the protocol and the container label. Supplies must be retained in a limited access area and under the appropriate environmental conditions as specified on delivery. Supplies should be dispensed under the supervision of the Principal Investigator or investigator. Local regulations should be adhered to.

All used material will be discarded at the clinical site and unused investigational medication will be returned to the sponsor or will be passed over for destruction on site (conform local regulations), or by an authorized destruction unit after authorization by the sponsor. This will be documented on the Drug Return Form, and a destruction certificate, if applicable.

7.2 Trial Documents

The following documents must be provided to the sponsor or representatives before shipment of investigational vaccines to the clinical unit.

- A signed and dated protocol and amendment(s), if any;
- A copy of the signed and dated written IEC approval specifying the documents being approved: the protocol, amendments, Informed Consent Form, any other written information provided to the subject, and subject recruitment materials. This approval must clearly identify the trial by protocol title and trial number;
- Regulatory authority approval or notification, if required;
- Documentation on which the assessment of the Principal Investigator's qualifications was based (i.e., curriculum vitae).

The following documents must be provided to the sponsor or representatives prior to enrolment of the first subject.

- The names of the current members or composition of the IEC and their position in the health-care institution or their credentials. In case the (sub) investigator is a member of the IEC, documentation must be obtained to state that this person did not participate in the voting for the trial;
- Signed and dated trial agreement, if applicable;
- Signed and dated financial agreement;
- Documentation on which the assessment of the investigators' qualifications was based (i.e., curriculum vitae);
- Current laboratory normal ranges for all tests required by the protocol that will be performed;
- Laboratory documentation demonstrating competence and test reliability (i.e., accreditation/license), if applicable.

7.3 Participation Cards

Since the subjects are not under 24-hour supervision of the investigator or his/her staff (out-subjects), they will be provided with a Subject Participation Card indicating the name of the investigational product, the trial number, the investigator's name and a 24-hour emergency contact number. The subject will be advised to keep the participation card in her wallet at all times. Information on the HIV-1 vaccination will be explained including the consequences for standard HIV-1 screening.

7.4 Source Data

The nature and location of all source documents will be identified in the Source Document Verification Plan to ensure that all sources of original data required to complete the CRF are known and are accessible for verification by the monitor. If electronic records are maintained, the method of verification must be discussed and agreed upon between the investigational staff and the monitor.

The required source data are listed in the Source Data Verification Plan, and should include sequential notes containing at least the following information for each subject:

- Subject identification (name, date of birth, gender);
- Documentation that subject meets eligibility criteria, i.e., history, physical examination, and confirmation of diagnosis (to support inclusion and exclusion criteria);
- Participation in trial (including trial number);
- Trial discussed and date of informed consent;
- Dates of all visits;
- Documentation that protocol specific procedures were performed;
- Results of efficacy parameters, as required by the protocol;
- Start and end date (including dose regimen) of trial treatment (preferably drug dispensing and return should be documented as well);
- Record of all AEs and other safety parameters (start and end date, and preferably including causality and intensity);
- Concomitant medication (including start and end date, dose if relevant; dose changes should be motivated);
- Date of trial completion and reason for early discontinuation, if applicable.

It is recommended that the author of an entry in the source documents is identifiable. Direct access to source documentation (medical records) must be allowed for the purpose of verifying that the data recorded on the CRF are consistent with the original source data.

7.5 Case Report Forms

CRFs are provided for each subject. All forms must be filled out legibly in durable black ballpoint pen.

Data Management may make changes to the entries made by the site (self-evident corrections or global rulings such as correction of obvious spelling errors). If global rulings (self-evident corrections) are applied to the clinical database by data management during the trial, the rules will be documented in a global rulings document and provided to the site for review and sign-off prior to applying the global rulings. If required, an investigator may request a detailed list of corrections applied to the data from his/her site through the trial monitor.

All data must be entered in English.

All data relating to the trial must be recorded on CRFs prepared by a qualified vendor under the supervision/responsibility of Kinesis Pharma BV. These CRFs should always reflect the latest observations on the subjects participating in the trial. Therefore, CRFs are to be completed as soon as possible after (or during) the subject's visit. To avoid interobserver variability, every effort should be made to ensure that all efficacy evaluations are completed by the same individual who made the initial baseline determinations. The investigator must verify that all data entries on the CRFs are accurate and correct. If some assessments are not done, or if certain information is not available, not applicable, or unknown, the investigator must enter “ND”, “NAV”, “NAP”, or “UN/UNK”, respectively, in the appropriate space.

During monitoring visits, the monitor will review the CRFs and evaluate them for completeness, legibility and consistency. The CRF will be compared with the source documents to ensure that there are no discrepancies between critical data. All entries, corrections, and alterations are to be made by the responsible investigator or his/her designee. The monitor cannot write on the CRF pages. Corrections

must be made in such a way that the original entry is not obscured. Correction fluid must NOT be used. The correct data must be inserted, dated, and initialled by the person making the correction. The persons entering data on the CRFs, must be identified on the Signature Sheet. The investigational staff must not write on NCR copies of CRFs left at the site once the original is transmitted to the sponsor or representative.

A CRF must be completed and the original must be returned to the sponsor or representative. A copy must be archived by the investigator as specified in section 7.7.

In case corrections to a CRF are needed after removal of the original CRF copy from the site, a Data Correction Form (DCF) will be used. All DCFs sent to the investigator will be answered by the appropriate investigational staff and signed and dated for approval.

7.6 Subject Identification Code List & Subject Screening and Enrolment Log, and CRF Log

In order to permit easy identification of the individual subject during and after the trial, the investigator is responsible for keeping an updated Subject Identification Code List. The monitor will review this document for completeness. However, in order to ensure subject confidentiality, this document will remain at the center and no copy will be made.

A Subject Screening and Enrolment Log that report all subjects that were seen to determine eligibility for inclusion in the trial also has to be completed by the investigator.

7.7 Archiving

The investigator shall maintain the trial documents as specified in “Essential Documents for the Conduct of a Clinical Trial” (ICH E6; 8.2 - 8.4) and as required by the applicable regulatory requirement(s). The investigator should take measures to prevent accidental or premature destruction of these documents.

Essential documents should be retained until at least 2 years after the last approval of a marketing application in an ICH region, and until there are no pending or contemplated marketing applications in an ICH region, or at least 2 years have elapsed since the formal discontinuation of clinical development of the investigational product. These documents should be retained for a longer period however, if required by the applicable regulatory requirements.

It is the responsibility of the sponsor to inform the investigator/institution as to when these documents no longer need to be retained.

Under no circumstance shall the investigator relocate or dispose of any trial documents before having obtained a written approval of the sponsor.

If it becomes necessary for the sponsor or the appropriate regulatory authority to review any documentation relating to this trial, the investigator must permit access to such reports. The subject is granting access to her source data by signing the informed consent.

Any difficulty in storing original documents must be discussed with the monitor prior to the initiation of the trial.

8 Confidentiality

All information concerning the product and the sponsor’s operations (such as patent applications, formulae, manufacturing processes, basic scientific data, or formulation information supplied to the investigator by the sponsor and not previously published) is considered confidential by the sponsor and should not be disclosed by the investigator to any third party without the sponsor’s prior written

approval. The investigator agrees to use this information only in accomplishing the trial and will not use it for other purposes.

9 Reporting and Publication

9.1 Reporting

Kinesis will create an Annual Safety Report (ASR) and Line Listings in accordance with the EU Clinical Trials Directive. This study (MYM-V101-CT08-101) will be included in the first ASR produced after approval of this protocol and in all subsequent ASRs, as appropriate, until study closure.

The results of the trial will be reported in a single Clinical Research Report. A summary of the final report will be provided to the investigators, to the applicable regulatory authorities, and IECs, if required by the applicable regulatory requirements, within 1 year after end of trial.

The Principal Investigator will be appointed for signing off the final Clinical Research Report.

9.2 Publication

The Sponsor will not unreasonably withhold consent to publish the data generated in this trial. However, it is the policy of the Sponsor not to allow the investigators to publish their results or findings prior to the Sponsor's publication of the overall trial results. The investigator agrees that before he/she publishes any results of this trial, he/she shall allow at least 45 days for the Sponsor to review the prepublication manuscript prior to submission of the manuscript to the publisher, as specified in the Clinical Trial Agreement between Institution/Investigator and Sponsor. In accordance with generally recognized principles of scientific collaboration, co-authorship with any company personnel will be discussed and mutually agreed upon before submission of a manuscript to a publisher.

10 Authorities/Ethics

10.1 Regulatory Authorities

This trial will be submitted to the local regulatory authority for approval or notification whichever is applicable. The trial will only be undertaken in compliance with the local regulatory requirements.

10.2 Independent Ethics Committee

This trial can only be undertaken after full approval of the protocol, informed consent, any other written information given to subjects, and subject recruitment materials has been obtained from the IEC. This document must be dated and clearly identify the trial and the documents being approved, including the subject compensation programs.

During the trial the following documents will be sent to the IEC for their review:

- Changes to the Investigator's Brochure;
- Reports of AEs that are serious, unlisted and associated with the investigational drug;
- ASR and line listings.

Substantial amendments and applicable Informed Consent Form revisions must promptly be submitted to the IEC for review and approval prior to implementation of the change(s), except when necessary to eliminate an immediate hazard to the trial subjects.

The IEC is responsible for continuous review of the trial. At least once a year, the investigator will provide the IEC with a progress report to allow review of the trial. Additional progress reports should be provided if required by the IEC.

These requests and (re)approvals, if applicable, should be documented in writing.

10.3 ICH-GCP Guidelines

This trial will be conducted in accordance with the current ICH-GCP Guidelines.

Good Clinical Practice (GCP) is an international ethical and scientific quality standard for designing, conducting, recording, and reporting trials that involve the participation of human subjects. Compliance with this standard provides public assurance that the rights, safety and well being of trial subjects are protected, consistent with the principles that have their origin in the Declaration of Helsinki, and that the clinical trial data are credible.

10.4 Subject Information and Informed Consent

Prior to entry in the trial, the investigator or a person designated by the investigator must explain to potential subjects the trial and the implications of participation. Subjects will be informed that their participation is voluntary and that they may withdraw from the trial at any time. Finally, they will be told that their records may be accessed by the IEC, regulatory authorities and authorized representatives of the sponsor without violating the confidentiality of the subject, to the extent permitted by the applicable law(s) and/or regulations. By signing the Informed Consent Form, the subject is authorizing such access.

The subject will be given sufficient time to read the Informed Consent Form and to ask additional questions. After this explanation and before entry in the trial, consent should be appropriately recorded by means of the subject’s personally dated signature. After having obtained the consent, a copy of the signed and dated informed consent must be given to the subject.

Any information relevant to the subject’s willingness to participate in the trial will be provided to the subject in a timely manner by means of an updated Informed Consent Form. This amended Informed Consent Form will be signed and dated by the subject and the investigator to document the willingness of the subject to continue with the trial.

This signed and dated amended version will be filed together with the initial signed and dated Informed Consent Form.

10.5 Privacy of Personal Data

The processing of personal data in pursuit of this trial will be limited to those data that are reasonably necessary to investigate the efficacy, safety, quality, and utility of the investigational product(s) used in this trial. These data will be processed with adequate precautions to ensure confidentiality.

The sponsor ensures that the personal data are:

- Collected for a specified and legitimate purpose;
- Processed fairly and lawfully;
- Credible.

Explicit consent for the processing of personal data will be obtained from the participating subjects prior to any processing of personal data.

The sponsor or its representatives whose responsibilities require access to personal data are obliged to keep the identity of trial subjects confidential. This confidentiality will be maintained throughout the

complete data processing. Trial subjects will be entitled to request confirmation of the existence of personal data held by the sponsor and will have the right to rectify erroneous or inaccurate data.

11 Financing and Insurance

11.1 Financial Disclosure

The disclosed financial interest of the investigator must be collected before enrolment of the first subject, following center completion, and 1 year following trial completion. The investigator should promptly update this information if any relevant changes occur during this period.

Disclosable financial interests will be recorded on the Investigator Financial Disclosure Form.

Any investigator(s) added as investigational staff must complete the Investigator Financial Disclosure Form at the beginning of their participation in the trial. For any investigator(s) leaving the site prior to trial completion, an Investigator Financial Disclosure Form should be obtained at the end of their participation.

11.2 Indemnification

The sponsor undertakes to indemnify and hold harmless the investigator and his/her medical staff from any claim, demand or cost arising from the activities to be carried out in compliance with the protocol.

11.3 Insurance

Sponsor ensures that appropriate liability insurance is available covering injuries arising from the participation of the trial subject in this trial, as further specified in the Informed Consent and the Clinical Trial Agreement.

12 Data Quality Control/Assurance

The trial will be monitored according to the current Standard Operating Procedure for the monitoring of clinical trials.

Shortly before the trial starts, the monitor will meet with the investigator and all staff involved to review the procedures regarding trial conduct and recording the data on the CRF. During the trial, the investigator shall permit the monitor to verify the progress of the trial at the center as frequently as necessary. The investigator shall make the CRFs available, provide missing or corrected data and sign the CRFs. Key data transcribed onto the CRFs, such as the subject’s sex, date of birth, assessment dates, test results, etc., will be reviewed against source documents. Personal information will be treated as strictly confidential and will not be made publicly available. Any inconsistency between source data and data recorded on the CRF will be corrected.

Kinesis Pharma BV will ensure that appropriate Quality Control (QC) steps are included into the different clinical processes to guarantee adequate protection of the trial subjects and quality of the data.

An independent Quality Assurance (QA) department, regulatory authorities and/or IECs may review this trial. This implies that auditors/inspectors will have the right to inspect the clinical unit(s) at any time during and/or after completion of the trial and will have access to source documents, including the subject’s file. By participating in this trial, investigators agree to this requirement.

For any data transfer, measures will be undertaken to protect subject data handed over against disclosure to unauthorized third parties and subject confidentiality will be maintained at all times.

13 References

1. Zolla-Pazner S. Indentifying epitopes of HIV-1 that induce protective antibodies. Nature Rev 2004;4: 199-210.
2. Zwick, MB. The membrane-proximal external region of HIV-1 gp41: a vaccine target worth exploring. AIDS 2005;19: 1725-1737.
3. Huckriede A, Bungener L, Stegmann T, Daemen T, Medema J, Palache AM, Wilschut J. The virosome concept for influenza vaccines. Vaccine. 2005 Jul 8;23 Suppl 1:S26-38.
4. Moser C, Amacker M, Kammer AR, Rasi S, Westerfeld N, Zurbriggen R. Influenza virosomes as a combined vaccine carrier and adjuvant system for prophylactic and therapeutic immunizations. Expert Rev Vaccines. 2007 Oct;6(5):711-21.
5. De Bruijn IA, Nauta J, Gerez L, Palache AM. Virosomal influenza vaccine: a safe and effective influenza vaccine with high efficacy in elderly and subjects with low pre-vaccination antibody titers.Virus Res. 2004 Jul;103(1-2):139-45.
6. D'Acremont V, Herzog C, Genton B. Immunogenicity and safety of a virosomal hepatitis A vaccine (Epaxal) in the elderly. J Travel Med. 2006 Mar-Apr;13(2):78-83.
7. Poovorawan Y, Theamboonlers A, Chumdermpadetsuk S, Glück R, Cryz SJ Jr. Safety, immunogenicity, and kinetics of the immune response to a single dose of virosome-formulated hepatitis A vaccine in Thais. Vaccine. 1995 Jul;13(10):891-3.
8. Holzer BR, Hatz C, Schmidt-Sissolak D, Glück R, Althaus B, Egger M. Immunogenicity and adverse effects of inactivated virosome versus alum-adsorbed hepatitis A vaccine: a randomized controlled trial. Vaccine. 1996 Jul;14(10):982-6.
9. Genton B, Pluschke G, Degen L, Kammer AR, Westerfeld N, Okitsu SL, Schroller S, Vounatsou P, Mueller MM, Tanner M, Zurbriggen R. A randomized placebo-controlled phase Ia malaria vaccine trial of two virosome-formulated synthetic peptides in healthy adult volunteers. PLoS ONE. 2007 Oct 10;2(10):e1018.
10. Fleury S. Preclinical study MyMVP1: A six month vaccination program on monkeys (species Macaca mullata) for investigating the immunogenicity of the P1 petides grafted onto virosomes. Mymetics Internal Preclinical Report 2006.
11. Fleury S. Preclinical study MyMVP1/rgp41. A six month vaccination program on monkeys (species Macaca mullata) for investigating the *in vivo* protection induced by the P1 and rgp41 antigens. Mymetics Internal Preclinical Report 2008.
12. Leoni A-L. Eight-week Repeated Dose Toxicity Study in the Rat with MYM-V101 and IRIV (control virosomes) after repeated intramuscular administration including a 4-week recovery period. BSL Scientific Laboratories GmbH, Planegg, Germany. Study 080803 A. Preliminary Results 24 October 2008 and Draft Histopathology Report 07 September 2008.
13. Leoni A-L. Ten-week Repeated Dose Toxicity Study in the Rat with MYM-V101 and IRIV (control virosomes) after repeated intramuscular and i.n. administration including a 5-week recovery period. BSL Scientific Laboratories GmbH, Planegg, Germany. Study 080803 B. Preliminary Results 22 October 2008 and Draft Histopathology Report 08 October 2008.

14. Leoni A-L. Local Tolerance Study in Rabbits with MYM-V101 and IRIV (control virosomes) after repeated intramuscular injection one a week during 7 weeks. BSL Scientific Laboratories GmbH, Planegg, Germany. Study 080804, Draft report.

15. Glück U, Gebbers JO, Glück R. Phase 1 evaluation of intranasal virosomal influenza vaccine with and without Escherichia coli heat-labile toxin in adult volunteers. J Virol. 1999 Sep;73(9):7780-6.

16. Kozlowski PA, Lynch RM, Patterson RR, Cu-Uvin S, Flanigan TP, Neutra MR. Modified Wick Method Using Weck-Cel® Sponges for Collection of Human Rectal Secretions and Analysis of Mucosal HIV Antibody. JAIDS 24:297-309, 2000.

17. Adler M, Wacker R, Niemeyer CM. Sensitivity by combination: immuno-PCR and related technologies. Analyst 2008;133:702 - 718, DOI: 10.1039/b718587c.

18. Crotty S, Aubert RD, Glidewell J, Ahmed R. Tracking human antigen-specific memory B cells: a sensitive and generalized ELISPOT system. J Immunol Methods 2004;286:111-122.

19. Quiding-Jarbrink M, Lakew M, Nordstrom I, Banchereau J, Butcher E, Holmgren J, Czerkinsky C. Human circulating specific antibody-forming cells after systemic and mucosal immunizations: differential homing commitments and cell surface differentiation markers. Eur J Immunol 1995;25:322-327.

20. Bomsel M. Transcytosis of infectious human immunodeficiency virus across a tight human epithelial cell line barrier. Nat. Med 1997;3:42-47.

21. Alfsen A, Iniguez P, Bouguyon E, Bomsel M. Secretory IgA specific for a conserved epitope on gp41 envelope glycoprotein inhibits epithelial transcytosis of HIV-1. J Immunol 2001;166:6257-6265.

22. Alfsen A, Yu H, Magerus-Chatinet A, Schmitt A, Bomsel M. HIV-1-infected blood mononuclear cells form an integrin- and agrin-dependent viral synapse to induce efficient HIV-1 transcytosis across epithelial cell monolayer. Mol Biol Cell 2005;16:4267-4279.

23. Li M, Gao F, Mascola JR, Stamatatos L, Polonis VR, Koutsoukos M, Voss G, Goepfert P, Gilbert P, Greene KM, Bilska M, Kothe DL, Salazar-Gonzalez JF, Wei X, Decker JM, Hahn BH, Montefiori DC. Human immunodeficiency virus type 1 env clones from acute and early subtype B infections for standardized assessments of vaccine-elicited neutralizing antibodies. J Virol 2005;79:10108-10112.

24. Naranjo CA, Busto U, Sellers EM, Sandor P, Ruiz I, Roberts EA, Janecek E, Domecq C, Greenblatt DJ. A method for estimating the probability of adverse drug reactions. Clin Pharmacol Ther. 1981 Aug;30(2):239-45.

14 Addenda

14.1 Addendum 1: Cardiovascular Safety – Abnormalities

ECG

All important abnormalities from the ECG readings will be reported by the investigator on an individual basis.

Vital Signs[a]

The following abnormalities will be defined for vital signs:

Pulse (beats per minute):

- abnormally high: ≥ 120 bpm;
- abnormally low: ≤ 50 bpm;

DBP (mmHg):

- abnormally high: Grade 1 or mild: > 90 to < 100 mmHg;
  Grade 2 or moderate: ≥ 100 to < 110 mmHg;
  Grade 3 or severe: ≥ 110 mmHg;
- abnormally low: ≤ 50 mmHg;

SBP (mmHg):

- abnormally high: Grade 1 or mild: > 140 to < 160 mmHg;
  Grade 2 or moderate: ≥ 160 to < 180 mmHg;
  Grade 3 or severe: ≥ 180 mmHg;
- abnormally low: ≤ 90 mmHg.

[a] The classification of AEs related to hypotension and hypertension will be done according to the CTCAE grading scale.

14.2 Addendum S2: Vaccination Proposition during Menstrual Cycle.

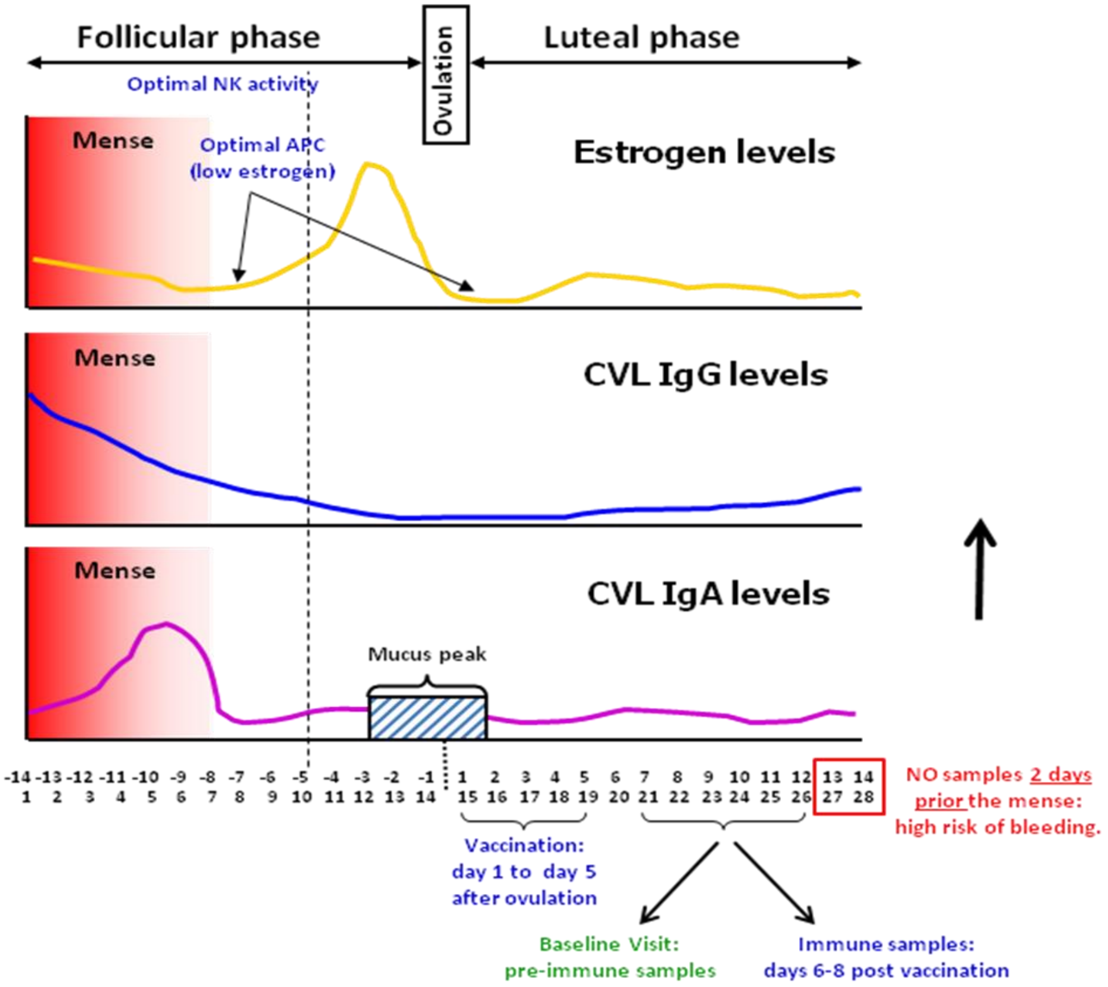

Important Note!

- Avoid sampling during the bleeding period to avoid contamination of mucosal secretions by antibodies that originate from the blood.
- Avoid sampling during the early mucus peak that traps antibodies.
- The ideal specific mucosal antibody peak should be between day 7-10 post-vaccination.
- The ideal PBL sample for monitoring mucosal B cells is between day 6-8 after vaccination.
